# Global and local meteoric water lines for *δ*^17^O/*δ*^18^O and the spatiotemporal distribution of *Δ*′^17^O in Earth’s precipitation

**DOI:** 10.1038/s41598-023-45920-8

**Published:** 2023-11-04

**Authors:** Stefan Terzer-Wassmuth, Luis J. Araguás-Araguás, Leonard I. Wassenaar, Christine Stumpp

**Affiliations:** 1 International Atomic Energy Agency, Department of Nuclear Sciences and Applications, Division of Physical and Chemical Sciences, Isotope Hydrology Section, Vienna International Centre, PO Box 100, 1400 Vienna, Austria; 2 Department of Water, Atmosphere and Environment, Institute of Soil Physics and Rural Water Management, University of Natural Resources and Life Sciences, Vienna, Muthgasse 18, 1190 Vienna, Austria; 3 Present Address: WasserCluster Lunz Biologische Station GmbH, Dr. Carl Kupelwieser Promenade 5, 3293 Lunz Am See, Austria

**Keywords:** Geochemistry, Hydrology

## Abstract

Recently, *δ*^17^O and its excess (*Δ*′^17^O) have become increasingly significant “triple-oxygen-isotope” indicators of distinctive hydrological processes in hydrology and climatology. This situation mirrors the research regarding *δ*^18^O and *δ*^2^H in the 1960s towards a solid theoretical base and a surge in application examples and field studies worldwide. Currently, systematic global measurements for *δ*^17^O in precipitation are still lacking. As a result, attempts have been made to define a Global *δ*^17^O/*δ*^18^O Meteoric Water Line (GMWL), often by using regional or local datasets of varying systematicity. Different definitions of the global reference slope (*λ*_ref_) for determining *Δ*′^17^O values have been proposed, by ongoing debate around a proposed consensus value of 0.528. This study used worldwide samples archived in the IAEA Global Network of Isotopes in Precipitation (GNIP) to (a) derive a *δ*^17^O/*δ*^18^O GMWL based on four-year monthly records from 66 GNIP stations, (b) formulate local *δ*^17^O/*δ*^18^O meteoric water lines (LMWL) for these stations’ areas, and (c) evaluate regional and seasonal variations of *Δ*′^17^O in precipitation. The GMWL for *δ*^17^O/*δ*^18^O was determined to be *δ*′^17^O = 0.5280 ± 0.0002 *δ*′^18^O + 0.0153 ± 0.0013, in keeping with the consensus value. Furthermore, our results suggested that using a line-conditioned ^17^O-excess is a viable alternative over the global *λ*_ref_ in the context of regional hydrology and paleoclimatology interpretations; however, without challenging the global *λ*_ref_ as such.

## Introduction

### Background

After more than 65 years of research focusing on the more abundant heavy isotopes of the water molecule (^18^O and ^2^H) in diverse hydrological and paleoclimatic applications, recent years have seen new interest in ^17^O, the rarest naturally occurring stable oxygen isotope^1–4^. The so-called “triple-oxygen-isotope” applications (^18^O/^17^O/^16^O) focus on the relationship between the ^17^O and ^18^O isotopes (^17^O-excess^5^). The ^17^O-excess is anticipated to provide novel insights into hydrological investigations (e.g., closing lake water balances^6,7^) and ice-core-based climatological research^8^ where the relative insensitivity of the ^17^O-excess to temperature-controlled isotope fractionation effects makes it suitable as an independent tracer of (paleo-) humidity conditions and other processes. However, one critical gap hampering environmental interpretations of ^17^O-excess in the hydrological sciences is the absence of robust local or global *δ*^17^O/*δ*^18^O meteoric water lines for Earth’s precipitation, the source of all water impacting terrestrial hydrological and climatological systems^2^. Currently, ^17^O-excess applications in hydrology suffer from an “emerging tracer dilemma”, a two-fold problem of missing systematic global baseline *δ*^17^O data in precipitation for making sound scientific interpretations and hypotheses coupled with ongoing analytical challenges, including the absence of certified *δ*^17^O values for the primary reference waters. In the absence of robust baseline datasets for *δ*^17^O in precipitation, global and local meteoric reference lines for *δ*^17^O are incomplete, unsystematic, or need to be inferred using proxies. In this study, we present the first global baseline for *δ*^17^O and ^17^O-excess values in Earth’s precipitation based on multi-year archived monthly precipitation samples (n = 3441) from 66 stations in the IAEA’s Global Network of Isotopes in Precipitation (GNIP)^9^, with the aim to:

(i) Establish a robust *δ*^17^O/*δ*^18^O Global Meteoric Water Line (GMWL) based solely on precipitation and amount-weighted isotope values obtained from multi-year GNIP station collections, aiming to align with existing definitions,
(ii) Construct *δ*^17^O/*δ*^18^O Local Meteoric Water Lines (LMWL) for a wide range of geographical locations with differing spatial and hydro-climatological characteristics, investigating their potential connections with spatial, temporal, and climatic factors,
(iii) Examine the spatial and seasonal distributions and variations of ^17^O-excess in global precipitation exploring its potential relationships with δ18O values and climatic factors, and
(iv) Introduce and demonstrate the concept of “line-conditioned ^17^O-excess” for use in local and regional hydrological applications and interpretations.

Our treatment of *δ*^17^O and ^17^O-excess data leverages data processing practices commonly applied for precipitation *δ*^18^O and *δ*^2^H, including the definition of GMWL^10^, isotope “excess”^11^, precipitation-amount weighted GMWL^12^ and LMWL^13,14^ and the minimum sampling periods required for determining a robust LMWL^15^. The concept of “line-conditioned ^17^O-excess” follows the recommendations for *δ*^18^O/*δ*^2^H data^16^. Unless stated otherwise, all terminology and equations are found in the supplementary material S1.

### The λ_ref_ coefficient and ^17^O/^18^O slope

The tiny changes in ^17^O relative to ^18^O through hydrological processes for Earth’s natural waters are described by the ^17^O-excess (*Δ*′^17^O) equation:

1 $$\Delta^{^{\prime}17} O = \delta^{^{\prime}17} O - \lambda_{ref} \delta^{^{\prime}18} O$$

The prime term δ′ is defined as:

2 $$\delta^{\prime } = {\text{ ln}}\left( {\delta /{1}000 + {1}} \right) \, \times { 1}000$$

The *λ*_ref_ value in Eq. (1) is considered a fixed isotope fractionation factor associated with the common mass-dependent hydrological processes (e.g., vapor-to-liquid phase, ice formation, etc.). This equation also implies that most water phase transformations result in *Δ*′^17^O values close to zero. Still, some processes like evaporation, molecular diffusion, or non-mass dependent effects differentially change the ^17^O/^18^O ratio and can lead to small but important changes in *Δ*′^17^O. The *λ*_ref_ coefficient value has a complicated history as detailed below and in reference^1^. Moreover, distinguishing between the ratio *θ* (of ^17^O and ^18^O isotope fractionation factors), and different regression line slopes *λ*_ref_, *λ*_(g)mwl,_ and *λ*_obs_ is also critical for interpreting the isotopic composition of Earth’s meteoric waters despite their close similarity^2^. These slope considerations are essential because *λ*_(g)mwl_ incorporates all atmospheric processes affecting *θ*, whereas the *λ*_obs_ slope includes that and any subsequent post-precipitation hydrological processes altering precipitation’s *λ*_(g)mwl_ signal. The *λ*_ref_ used for *Δ′*^17^O determinations is generally assumed to be constant and independent of these other processes.

Early estimates of *θ* for ^17^O and ^18^O were 0.5^17^, but did not consider alterations due to other mass-dependent and -mass-independent oxygen isotope fractionation processes. Kinetic (e.g., evaporative) and equilibrium isotope fractionations (e.g., Rayleigh distillation) can induce offsets of *δ*^17^O compared to *δ*^18^O, thereby leading to a small “^17^O-excess”^5^. The first proposed “^17^O-excess” value was based on an oxygen isotope fractionation factor of *θ* = 0.52^18^. This *θ* value was later redefined to 0.5281 by incorporating a comprehensive set of terrestrial meteoric waters^19^, including surface, groundwater, polar snow, and ice core samples. The global meteoric “reference line” (including the *λ*_ref_ value for *Δ′*^17^O and ordinate intercept *γ*) of *δ*′^17^O = 0.528 *δ*′^18^O + 0.033^20^ is nowadays the most widely used GMWL definition. Notably, this global reference line definition was based on a relatively geographically limited set of terrestrial freshwater samples from diverse sources including precipitation, ground and surface waters, and ice with significant variability in the reported *Δ′*^17^O values.

More recently, concerns have been expressed about whether ultra ^17^O/^18^O-depleted polar snow and Vostok ice core samples^8,21^ untowardly biased the proposed consensus *λ*_ref_ value of 0.5281. After removing Antarctic data points, a new *λ*_ref_ value of < 0.528 was discussed as more accurately representing temperate and tropical precipitation^19–21^. The first GMWL excluding polar waters (with a lower *δ*^18^O cut-off at -20 ‰) resulted in the equation *δ*′^17^O = 0.5265 *δ*′^18^O + 0.014^22^. Recently, a *λ*_ref_ (for *Δ′*^17^O) was proposed based on a regression of an extensive compilation of meteoric water *δ*^18^O and *δ*^17^O values and a GMWL of *δ′*^17^O = 0.5268 *δ′*^18^O + 0.015 was obtained^2^. However, this dataset was biased substantially to (partially screened) surface waters; hence, the lower slope that was obtained could be an artifact of surface water evaporation. The authors highlighted the lack of spatiotemporal data for *δ*^17^O and *Δ′*^17^O in global precipitation as a significant concern and questioned whether correlations with *Δ′*^17^O and *d*-excess exist. The most recent *λ*_ref_ proposal^23^ fitted a regression line of *δ′*^17^O = 0.5272 *δ′*^18^O + 0.020 to a compilation of *Δ′*^17^O and *d*-excess in precipitation; however, also acknowledging the sampling- and analytical heterogeneity of their dataset.

### Available *δ*^17^O/*δ*^18^O LMWLs and precipitation *Δ*′^17^O data

To date, relatively few precipitation-based *δ*^17^O/*δ*^18^O LMWLs have been reported in the literature. The considerable heterogeneity of outcomes likely reflects the spatial variabilities (tropical to polar study sites), precipitation sampling methods (e.g., event- vs. monthly composite samples), laboratory analytical methods, and reference materials used for normalization. The *δ*^17^O/*δ*^18^O LMWL regression equations are sometimes reported with or without an intercept (i.e., forced through the origin). A summary of the literature LMWL *δ*^17^O/* δ*^18^O results is tabulated in Supplementary Materials S3. Many of these LMWLs’ *λ* values for tropical and temperate climates cluster around the reported consensus value of 0.528; however, considerable exceptions go in both directions. For several reported LMWLs (Table S4), crucial details of LMWL calculation methods (amount weighted yes/no, intercept yes/no) are unreported. Seasonal MWLs for four precipitation source regions/pathways^24^ and regional MWLs for multiple stations^25^ are also summarized.

Following the publication^20^ of the first “global” mean *Δ′*^17^O value for meteoric waters (+ 0.033 ‰), various efforts have studied its spatiotemporal distribution and relationship with air temperature and relative humidity. Spatial patterns of *Δ′*^17^O for a cross-section of USA tap (surface) waters revealed a distinctive latitudinal gradient with a mean *Δ′*^17^O value of + 0.015 ‰^26^. The spatiotemporal distribution of *Δ*′^17^O from tap water in China showed a latitudinal gradient but limited seasonal variability (*Δ′*^17^O of + 0.026 to + 0.047 ‰)^27^. No *Δ′*^17^O seasonal patterns were observed in a 2-year record of rain and snow in the continental USA^28^, but patterns were found in Switzerland^29^ and Spain^30^. In the tropics, the seasonality of *Δ′*^17^O in precipitation for Okinawa, Japan, showed a mixing of continental winter (*Δ′*^17^O of + 0.025 to + 0.050 ‰) with tropical monsoon air masses (*Δ′*^17^O of + 0.005 to + 0.025 ‰)^31^. Significant *Δ′*^17^O periodicities of three, six and 30 months were observed in a five-year *Δ′*^17^O precipitation dataset for Singapore^32^. In polar regions, *Δ′*^17^O was found to be correlated to air temperature at Vostok in Antarctica^33^, and *Δ′*^17^O seasonality correlated with *δ*^18^O in NEEM camp, Greenland^34^.

The relationship between *Δ′*^17^O and *d*-excess has been synthesized^2^ but *d*-excess is not always available (if *δ*^17^O and *δ*^18^O are measured by isotope-ratio mass spectrometry, a separate analysis is required to obtain *δ*^2^H). In this review, meteoric waters clustered around + 0.000 to + 0.040 ‰ *Δ′*^17^O and 0 to + 20 ‰ *d*-excess but without any discernible spatial or temporal patterns, nor relationships between the two “excesses”. Only by including substantially different endmembers (e.g., plotting precipitation against lake waters or plant waters), did distinct patterns emerge.

### Introducing the line-conditioned ^17^O-excess

Notably, most of the regional studies reported above adopted a *λ*_ref_ value of 0.528; yet some discuss the applicability of a *λ*_mwl_ as an alternative to *λ*_ref_ definitions. To avoid confusing *λ*_ref_ definitions, a line-conditioned excess (*lc*-excess^16^) could serve to “normalize the excess to a LMWL”, analogous to that of the *δ*^18^O/*δ*^2^H GMWL and *d*-excess. The aim of *lc*-excess in *δ*^18^O/*δ*^2^H applications is to better and quantitatively compare the isotopic enrichments of different surface waters in catchments having different precipitation isotopic input functions^16^, as expressed by mean *d*-excess, LMWL slope and intercept. The *lc* technique for ^17^O-excess may allow for improved data interpretation within a regional hydrological framework and may be better suited than applying a global concept (the *d*-excess^11^), without however questioning the validity of a consensus *λ*_ref_ as such.

For triple oxygen isotope application, the definition for line-conditioned ^17^O-excess (*Δ′*^17^O_lc_) is:

3 $$\Delta^{{\prime {17}}} {\text{O}}_{{{\text{lc}}}} = \delta^{{\prime {17}}} {\text{O }}{-}\lambda_{{{\text{mwl}}}} \times \delta^{{\prime {18}}} {\text{O }}{-}\gamma_{{{\text{mwl}}}}$$

This definition is appropriate to handle any local isotopic regression line (*λ*_mwl_ and *γ*_mwl_ could also be replaced by *λ*_obs_ and *γ*_obs_).

## Results

### The first precipitation-based *δ*^17^O/*δ*^18^O global meteoric water line

Based on the weighted mean *δ′*^17^O/*δ′*^18^O of 66 GNIP stations which met our minimum criteria for reproducible monthly integrated precipitation records (see also supplementary table S5), we obtained a weighted GMWL of:

4 $$\delta^{{\prime {17}}} {\text{O}} = 0.{528}0 \pm 0.000{2}\delta^{{\prime {18}}} {\text{O}} + 0.0{153} \pm 0.00{13}\quad ({\text{R}}^{{2}} { = 1},{\text{ p - value}} < 0.00{1},{\text{ n}}_{{{\text{Stat}}}} = {66},{\text{ n}}_{{{\text{Data}}}} = {2683),}$$

**Figure 1:**
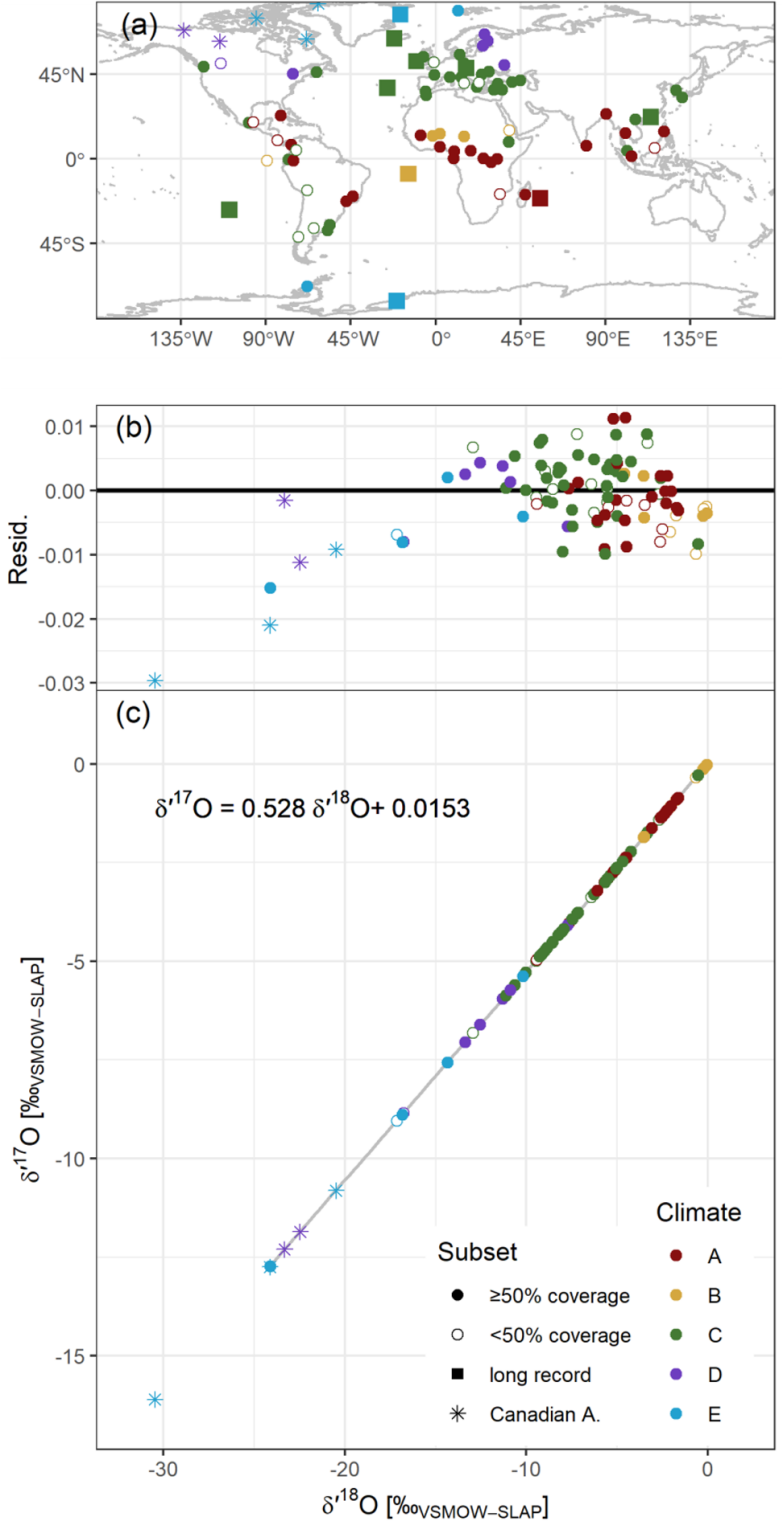
Map of GNIP stations analyzed (**a**). *δ*′^17^O / *δ*′^18^ GMWL by climatic zones (**c**) and *δ*′^17^O residuals (**b**).

Alternative approaches for deriving a GMWL were tested, including unweighted OLS (ordinary least squares) and reduced major axis (RMA) techniques, optionally forcing zero intercepts. These alternative approaches are summarized in Table 1. A cross plot and regression (Eq. 4) of these combined precipitation datasets by climatic zone and their *Δ*′^17^O residuals are shown in Fig. 1.

**Table 1:** Results of various methodological weighted and un-weighted approaches to determine GMWL definition using GNIP δ′^17^O/δ′^18^O values. OLS = ordinary least squares regression, RMA = reduced major axis.

Method	Stations with data:	n	Definition	R^2^	*p*-value
Weighted OLS	> 50% reproducible	66	*δ*′^17^O = 0.5280 *δ*′^18^O + 0.0153	1	< 0.001
Weighted OLS zeroed	> 50% reproducible	66	*δ*′^17^O = 0.5260 *δ*′^18^O	1	< 0.001
Unweighted OLS	> 50% reproducible	66	*δ*′^17^O = 0.5282 *δ*′^18^O + 0.0164	1	< 0.001
Unweighted RMA	> 50% reproducible	66	*δ*′^17^O = 0.5282 *δ*′^18^O + 0.0143	1	< 0.001
Weighted OLS	All excl. Canadian Arctic	83	*δ*′^17^O = 0.5279 *δ*′^18^O + 0.0143	1	< 0.001
Weighted OLS	All incl. Canadian Arctic	88	*δ*′^17^O = 0.5280 *δ*′^18^O + 0.0152	1	< 0.001

### Local meteoric water lines for 66 GNIP stations worldwide

**Figure 2:**
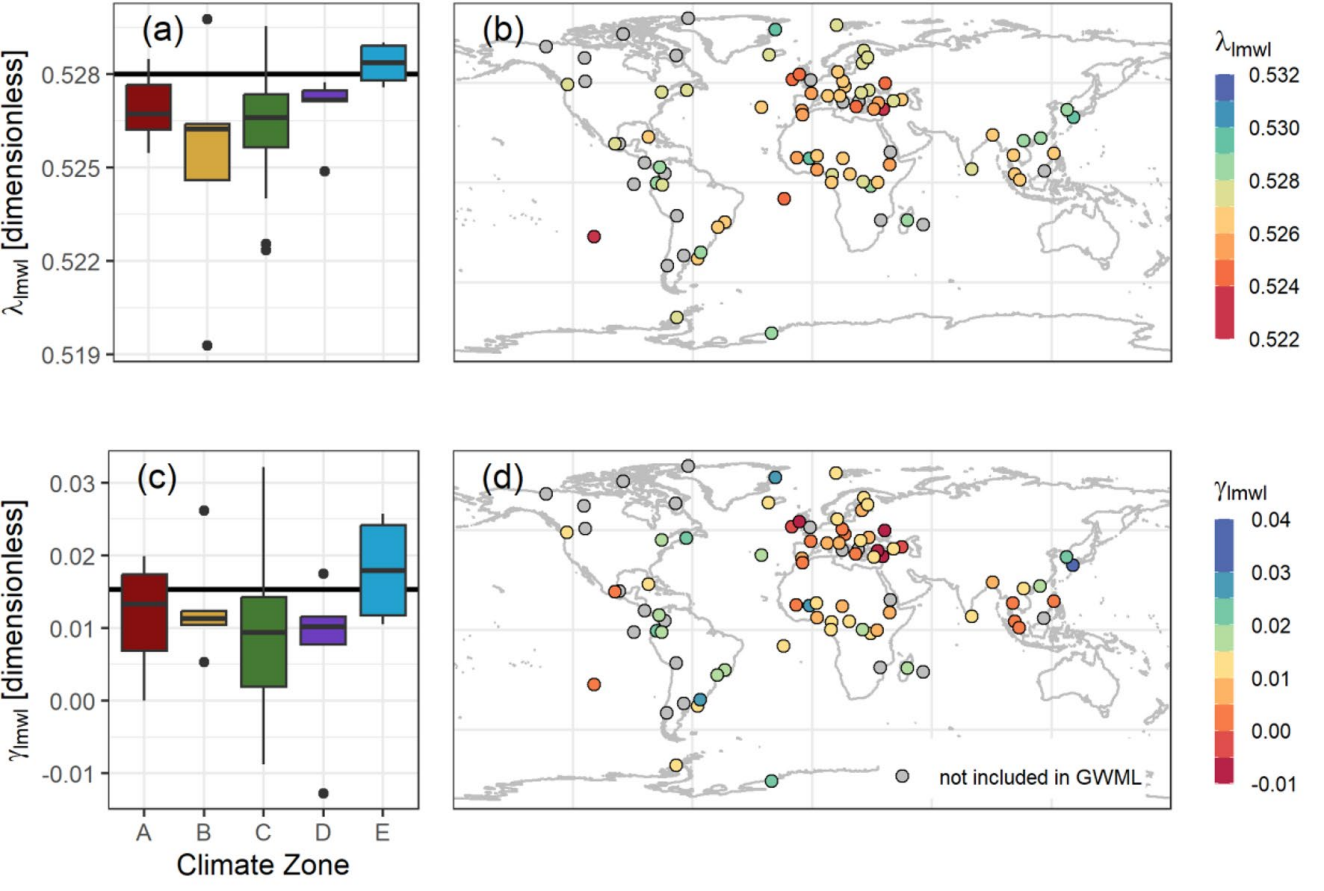
(**a**) LMWL slopes (*λ*_lmwl_) grouped by climatic zone, (**b**) *λ*_lmwl_ map, (**c**) LMWL intercepts (*γ*_lmwl_) by climate zone, (**d**) *γ*_lmwl_ map.

Our dataset allowed us to construct *δ*^17^O/*δ*^18^O local MWLs for the 66 GNIP stations spanning tropical to polar climates. Figure 2 depicts the spatial and climatic distribution of the *δ*^17^O/*δ*^18^O slopes (*λ*_lmwl_) and their intercepts (*γ*_lmwl_). A complete tabulation of these LMWLs is found in Supplementary material S6.

By grouping the LMWL slopes of the GNIP stations by their synoptic climatology (Köppen-Geiger classification^35^), we observed a tendency of lower *δ*^17^O/*δ*^18^O slopes and intercepts going from polar to dry (sub-) tropical climates (E to Bxh, supplementary table S5). However, the tropical stations (A climates) yielded higher *δ*^17^O/*δ*^18^O slopes and intercepts. For the A and C climate zones, where higher data density permitted parsing out the “wettest” fraction (mean monthly precipitation > 200 mm and > 100 mm, respectively), the *δ*^17^O/*δ*^18^O slopes and intercepts tended to be higher than for the overall dataset (see table S7).

**Figure 3:**
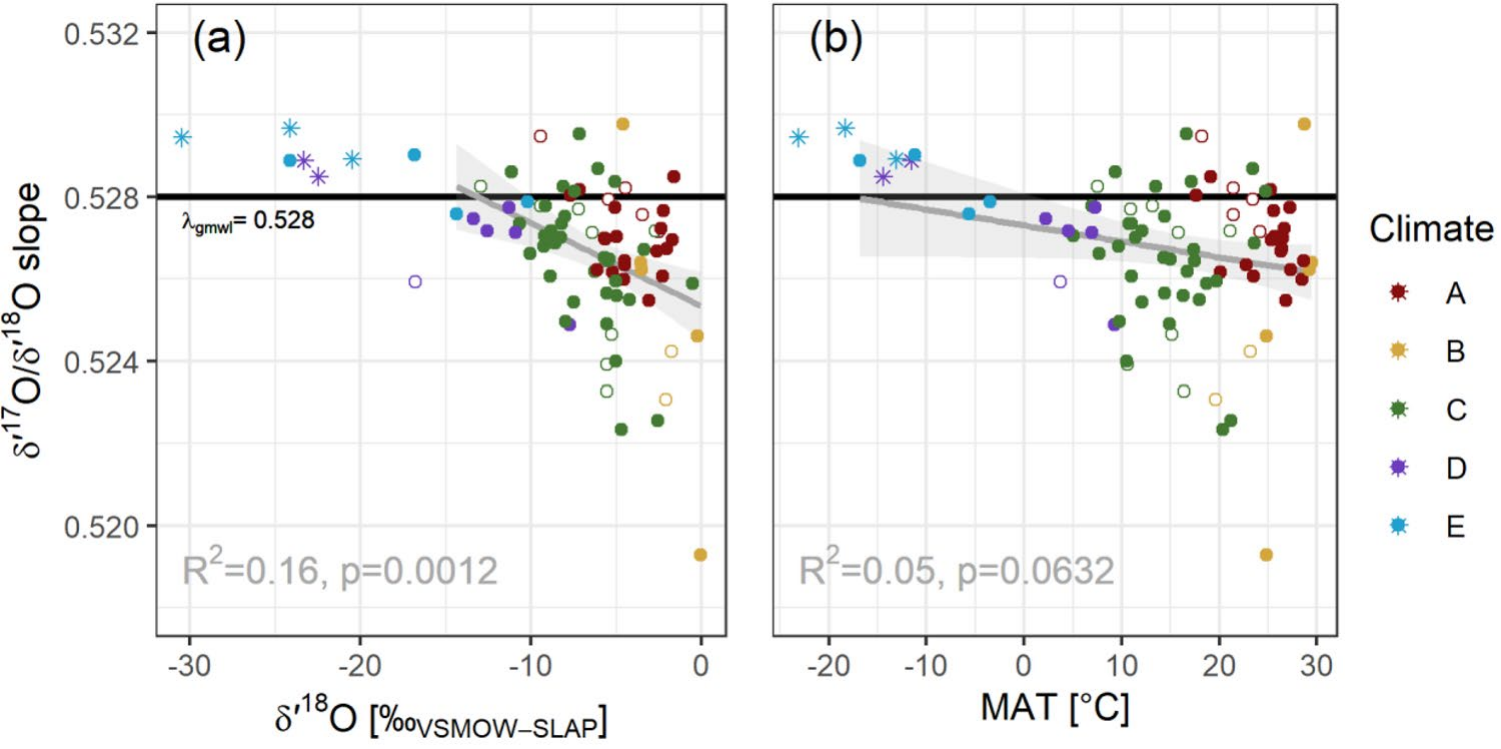
*δ*′^17^O/*δ*′^18^O slope against (**a**) *δ*^18^O and (**b**) mean annual temperature (MAT).

Screening the data for meteorological drivers of the *δ*^17^O/*δ*^18^O slopes beyond synoptic climatology (Fig. 3), we examined the correlations between the weighted MWL *δ*^17^O/*δ*^18^O slopes with stations’ mean annual (a) *δ*^18^O in precipitation and (b) air temperature. A significant relationship with mean annual air temperature (MAT) was observed only for stations with between 20 mm < mean PPT < 80 mm and MAT < 30 °C (R^2^ = 0.63; after removal of tropical islands and Hong Kong). Too few data points compromised our attempts to determine air temperature relationships for the polar climate MWLs with *δ*^17^O/*δ*^18^O slopes (MAT < 10 °C).

Similarly, by regressing the *λ*_lmwl_ values against *δ*^18^O values, a fairly low R^2^ of 0.30 was determined for extratropical stations (Köppen-Geiger types B to E). This finding was surprising because relating the mean *δ*^18^O or *δ*^17^O values to air temperature for this set of stations yielded an R^2^ = 0.88 (R^2^ = 0.65 without removing the rainiest sites having > 120 mm mean monthly precipitation). This suggested that other influential factors occur in the interplay of *δ*^17^O and *δ*^18^O in precipitation that cannot be detected when considering both oxygen isotopes independently. We could not identify any significant relationship between the MWL *δ*^17^O/*δ*^18^O slope and relative humidity (RH); however, this could be due to the precipitation amount weighting assigning a higher importance to the rainier (and usually more humid) months. Further information and Figures describing the relationship between *λ*_mwl_ and meteorological parameters are available in SM7.

### Global distribution of *Δ*′^17^O in earth’s precipitation

**Figure 4:**
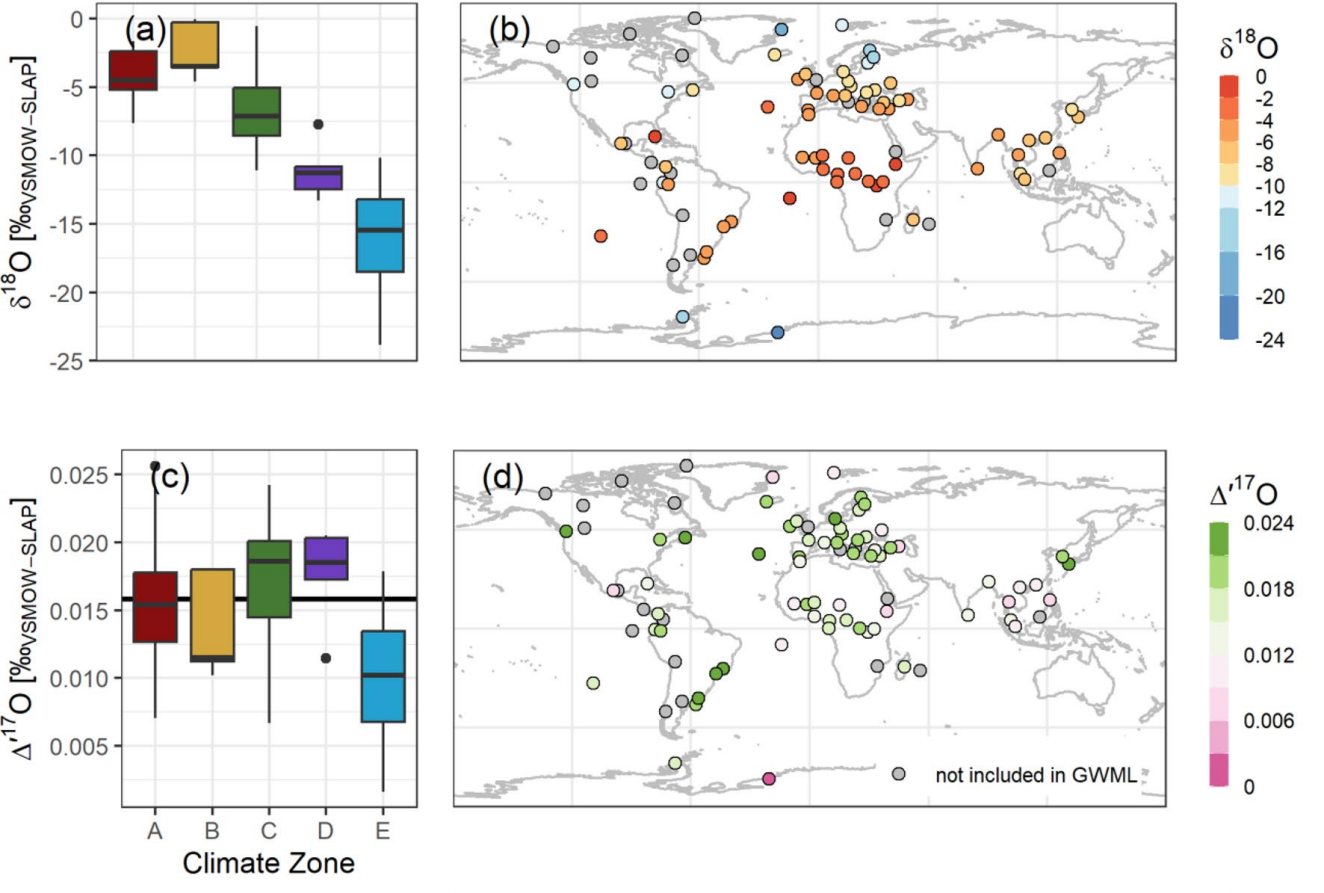
(**a**) and (**c**) Station mean *δ*^18^O and *Δ*′^17^O by climatic zone; (**b**) and (**d**) Spatial distribution of *δ*^18^O and *Δ*′^17^O.

The weighted mean *Δ′*^17^O for precipitation of the GNIP sites analyzed is shown in Fig. 4 (red circles are sites with < 50% isotopic coverage). The global weighted mean *Δ′*^17^O value was determined to be + 0.016 ‰, which agreed with the *γ*_gmwl_ of our GMWL definition. Unlike for *δ*^18^O, no significant global spatial patterns for *Δ′*^17^O were discernible. The mean *Δ′*^17^O values tended to be above the global average only in the temperate climatic zones.

**Figure 5:**
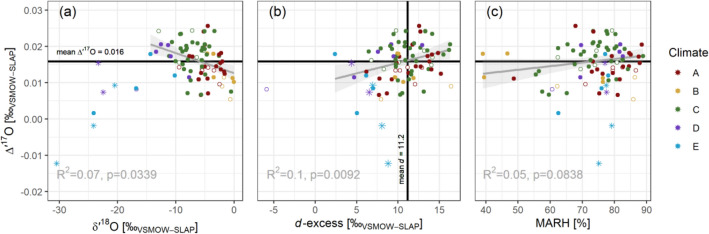
Weighted mean *Δ*′^17^O against (**a**) *δ*′^18^O, (**b**) *d*-excess and (**c**) MARH (mean annual RH).

Going beyond the synoptic climatology, we explored the relationships between *Δ′*^17^O, *δ*^18^O and *d*-excess values (Fig. 5). These were like the precipitation data in earlier reviews^2^; however, our dataset included only two cold climate locations with annual mean *δ*^18^O < -15 ‰. A correlation analysis did not show a significant correlation between *Δ′*^17^O and *d*-excess values (R^2^ = 0.09, *p* = 0.0043), and all data points clustered around a global mean *Δ′*^17^O =  + 0.016 ‰ and a global mean *d*-excess =  + 11.2 ‰. Our finding was consistent with earlier studies that also included more samples from cold climates^2^.

We investigated whether *Δ′*^17^O reflects large-scale hydroclimatic patterns, akin to *δ*^18^O, whose temperature correlation has been historically and implicitly been used as a climatic proxy^8,10–13,20,33,33^. A strong correlation of station mean *Δ′*^17^O with MAT would have suggested a similar temperature control for *Δ′*^17^O; yet the poor correlation suggested different, possibly more local or regional hydrological processes, corroborating atmospheric circulation modelling efforts^36^. In our dataset, the mean *Δ′*^17^O of the GNIP stations correlated poorly with mean air temperature, mean annual RH, mean weighted RH, and precipitation amount (Fig. 5 (c) and SM Figure S5). The potential influence of meteorological processes on the *Δ′*^17^O at short(er) spatiotemporal scales^24,30,31^, which is smoothed out in the monthly sampling regime, remains speculative based on the monthly composite samples analyzed in this work.

### Seasonal *Δ*′^17^O patterns and LMWLs

We reveal the first regional-seasonal assessment of *Δ′*^17^O based on multi-year GNIP precipitation isotope datasets. For 12 of the 66 GNIP stations with more extensive time-series, we created a selection of seasonal MWLs (warmest/coldest or wettest/driest trimesters), to explore their potential as baselines where a distinct isotopic response to large-scale atmospheric circulation patterns is known to occur seasonally. An overview table and plots are presented in supplementary materials table S8 and figure S3. Where a temperature correlation with *δ*^17^O and *δ*^18^O was found for a station, the summer *δ*^17^O/*δ*^18^O MWLs tended to have lower slopes and intercepts than the winter ones (e.g., Reykjavik, Valentia, Vienna, Danmarkshavn and Halley Bay). These stations are just a few examples showing a robust relationship between mean air temperature and *δ*^17^O/*δ*^18^O with the MWL slopes. Below we discuss the regional/seasonal patterns for the Mediterranean basin and (south-)east Asia and several oceanic island locations. Figures, tables, and brief comments for six further regions are available from SM S8.4–8.9.

For Mediterranean stations (for figures and tables see supplementary panel S8.1), the winter season (DJF) showed an elevated *Δ′*^17^O (around or >  + 0.020 ‰) but no longitudinal gradient, unlike for *d*-excess values. On the contrary, the spring samples (MAM) showed a higher to lower W-E *Δ′*^17^O gradient opposite to that of *d*-excess. Summer (JJA) followed similar patterns, but *d*-excess and *Δ′*^17^O values were the lowest among all seasons and some stations had no summer precipitation at all, e.g., Gibraltar. The fall season (SON) showed a W-E spatial gradient with higher values for *Δ′*^17^O and *d*-excess; however, neither of the above patterns proved to be significant. Data for Ancona at the Adriatic Sea coast presented an anomaly with higher *Δ′*^17^O values than the other sites; probably due to the Adriatic being a sub-basin inside the closed Mediterranean basin.

Our analysis of the *δ*^17^O and *Δ′*^17^O patterns in Southeast and East Asia (supplementary panel S8.2) differentiated between stations located in tropical climates (e.g., Bangkok, Diliman Quezon City, Johor Bahru and Cameron Highlands) and those with more temperate climates (hot: Hanoi, Hong Kong; warm: Kumamoto and Cheongju). During the winter period (JJA), *Δ′*^17^O values tended to be above the average for all stations but stood out in the northern two stations. During the summer (JJA), all station *Δ′*^17^O values clustered between + 0.005 ‰ and + 0.015 ‰ irrespective of their *d*-excess values. A latitudinal spatial gradient of increasing *Δ*′^17^O values from Bangkok/Diliman Quezon City over Hanoi/Hong Kong and towards Kumamoto/Cheongju was observed for all seasons (yet without statistical significance, probably due to the small number of data points). However, significant correlations with MAT were observed. We interpreted the lower *Δ′*^17^O value during summertime as signaling the advancing monsoon, in good agreement with Okinawa^28^ and reflecting the *δ*^18^O and *d*-excess patterns found earlier^37^. Notably, the retreating monsoon during the fall season gave the lowest of *Δ*′^17^O values for Bangkok. The only tropical GNIP station in Asia whose *Δ*′^17^O stood out was Cameron Highlands; it was the site at a higher altitude of 1440 m.a.s.l.

For small oceanic islands (supplementary panel S8.3), the lowest *λ*_lmwl_ values were observed on Ascension Island (including Travellers Hill), but also for the Galápagos and Réunion islands in the dry season, with *λ*_lmwl_ < 0.525. The *γ*_lmwl_ and seasonal *Δ′*^17^O values were mainly below the global average for these island sites and across all seasons. The tropical islands of Réunion and Ascension showed similar *Δ′*^17^O behaviors by clearly distinguishing the wettest and driest trimester, respectively. Though Réunion is classified as wet tropical, and Ascension is arid, the difference of seasonal MWLs was clear. The GNIP site at Travellers Hill (415 m.a.s.l.) on Ascension Island showed an inverse pattern for dry and wet seasons. No significant differences between the warmest and coldest trimesters were observed for the subtropical island stations (e.g., Pta. Delgada, Azores and Isla de Pascua, Pacific Ocean). Their *Δ′*^17^O values were also above the global mean value. The pattern of *λ*_lmwl_ slopes showed more scatter, though it was generally higher for wetter climates (> 0.527 for annual *λ*_lmwl_ in Havana and Diliman and all seasons in Pta. Delgada). In general, seasonal mean *Δ′*^17^O tended to be clustered with few outliers; the latter mainly in those island stations with strong precipitation seasonality. Also, the mean *Δ′*^17^O for all island sites was similar (mean of island stations =  + 0.013 ‰) and slightly below the global mean, supporting the idea that these stations were capturing an immediate oceanic-to-precipitation signal with little advective contributions.

### The line-conditioned ^17^O-excess (*Δ*′^17^O_lc_)

**Figure 6:**
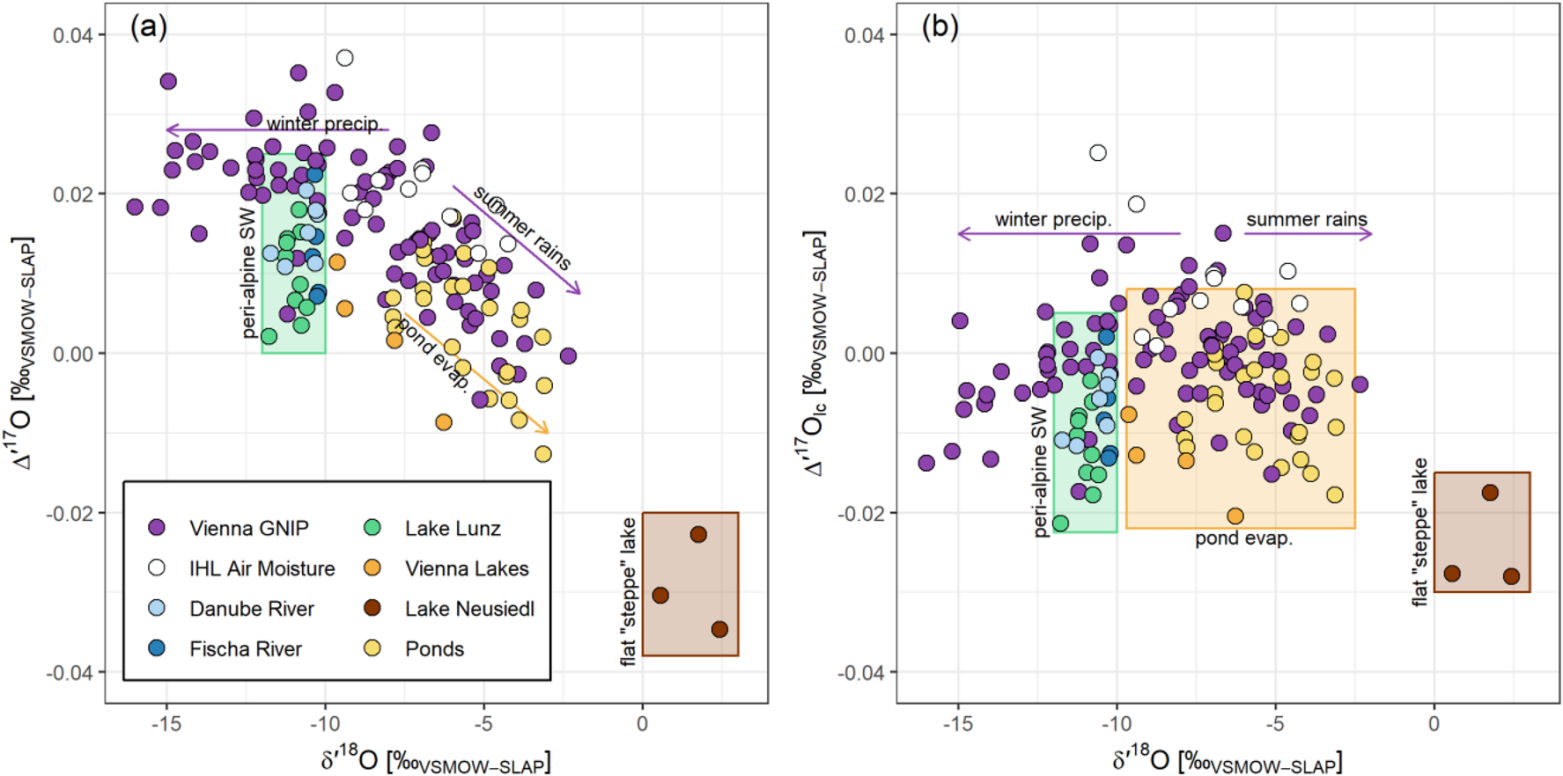
*Δ*′^17^O (**a**) and *Δ*′^17^O_lc_ (**b**) of surface waters compared to the Vienna precipitation dataset.

Adopting a line-conditioned ^17^O-excess (*Δ′*^17^O_lc_) was helpful to de-trend individual data points from the LMWL, e.g., lake waters. Figure 6 compares the *δ*^18^O/*δ*^17^O LMWL for the Vienna region (Austria) with surface waters sampled within a 100-km radius of the GNIP station, and laboratory air moisture condensates in terms of their (a) *Δ′*^17^O and (b) *Δ′*^17^O_lc_ values based on the Vienna LMWL. A description and location map of the samples is available in supplementary material S9. The use of *Δ′*^17^O_lc_ clearly aids in removing global precipitation patterns and helped to “normalize” regionally relevant hydrological conditions to a local precipitation station, as seen for these regional waters in Fig. 6. Theoretically, *Δ′*^17^O_lc_ values could also identify stratospheric intrusion; however, such occurrences remain debatable^20^ (see below). In any case, adopting the *Δ′*^17^O_lc_ concept eliminates aim for “*λ*_ref_-adjusted” *Δ′*^17^O definitions through a dedicated and specified local/regional framework equation. The spatial domain of an LMWL, and how to fall back onto the GMWL in absence of an LMWL, have been described earlier for *δ*^*18*^O/*δ*^2^H ^13,16^.

## Discussion

Our precipitation-based *δ*^17^O/*δ*^18^O Global Meteoric Water Line and dataset have provided new insights into global and local *δ*^17^O and *Δ′*^17^O spatiotemporal patterns. This GMWL definition agreed with the initially proposed *λ*^19,20^; but contrasts with other’s proposed GMWL *δ*^17^O/*δ*^18^O definitions^2,22,23^,. Nevertheless, our *γ*_gmwl_ was in good agreement with some studies ^2,22,23^ but not with others ^20^. Moreover, a *γ*_gmwl_ intercept equaling + 0.0153 ‰ was almost identical to the global weighted mean *Δ′*^17^O for all GNIP stations sampled (+ 0.016 ‰). The *γ* intercept can be explained by a slight isotopic shift during the evaporation of water from the ocean; however, our analysis found it to be of a lower magnitude than that previously proposed (+ 0.035 ‰^2^). Our GMWL definition is the first to be based exclusively on robust multi-year cumulative precipitation sample collections with substantial global geographical coverage, and did not include any potentially evaporated surface waters, ice or groundwater samples as in prior efforts. Our analysis also revealed the importance of (a) the calculation and regression method used (weighted vs. unweighted, intercept vs. zero-forced), (b) the level of sample aggregation (based on station mean values vs. raw data) and (c) the data curation used to construct each MWL. This is analogous to the well-documented *δ*^2^H/*δ*^18^O GMWL related discussions of the past^13,14^.

The number of cold climate stations sampled was small and excluded East Antarctica, where much of the data originates whose influence on earlier GMWLs was considered to be disproportional^22^. We acknowledge this to be a shortcoming in our global dataset; however, whilst polar areas cover a large land mass and exercise an important role in global atmospheric circulation, their precipitation contribution is below the global average and therefore, we are confident that their absence in a precipitation-weighted GMWL regression calculation is unlikely to cause major biases for most of the planet. The inclusion of additional data points from the Arctic did not substantially alter our GMWL definition (see Table 1); however, the representativeness of those data points came with a number of caveats (see SM 4.3 and 10).

To assess conditions under which a *λ*_gmwl_ < 0.528 could occur, we limited our assessment to GNIP stations with a mean isotopic composition of *δ*^18^O > -10 ‰, and the *λ*_gmwl_ was lowered to 0.5277. Only when constraining the GNIP dataset to those stations with mean *δ*^18^O values > -5 ‰, could a *λ*_gmwl_ of 0.5266 could be, which is clearly unrepresentative of a global MWL. Due to the few cold-climate stations with a mean *δ*^18^O < -20 ‰, it was impossible to calculate a robust polar MWL ^20^. We acknowledge the conventional logic that “a GMWL is the weighted mean of an infinite number of LMWLs”^13^ (such a thought experiment would result in a GMWL of *δ′*^17^O = 0.5268 *δ′*^18^O + 0.0085 based on our dataset) did not match the GMWL reported above and could be due to the uneven spatial distribution of GNIP sites, with more sites representing middle and lower latitudes.

The construction of Local Meteoric Water Lines for *δ*^17^O/*δ*^18^O based on multi-year GNIP station datasets is an alternative to competing GMWL definitions by providing a comprehensive reference-line framework. We consider this effort a first step towards a broader availability of a much-needed consensus *λ*_mwl_^2^. Our proposed LMWLs were in line with previous literature values (see Table 2, considering the differences in sampling approaches, laboratory analytics, data normalization and MWL calculation methods), except for the Southeast Asia Kolkata/Dhaka LMWLs^38^.

**Table 2:** Comparison of our GNIP-based LMWLs with those previously published in similar regions. Literature LMWLs with * were recalculated using data provided in supplementary materials, see Table S4.

Location	Literature LMWL	Closest in this dataset	LMWL, this work
Oozato, Okinawa^31^	*δ*′^17^O = 0.5304 *δ*′^18^O + 0.0377 *	Hong Kong	*δ*′^17^O = 0.5298 *δ*′^18^O + 0.0235
		Hanoi	*δ*′^17^O = 0.5281 *δ*′^18^O + 0.0116
		Kumamoto	*δ*′^17^O = 0.5295 *δ*′^18^O + 0.0322
Singapore^32^	*δ*′^17^O = 0.5271 *δ*′^18^O + 0.0154	Johor Bahru	*δ*′^17^O = 0.5262 *δ*′^18^O + 0
		Cameron Highlands	*δ*′^17^O = 0.5269 *δ*′^18^O + 0.004
Southern Peru^2^	*δ*′^17^O = 0.5275 *δ*′^18^O + 0.031 *	Viacha	*δ*′^17^O = 0.5282 *δ*′^18^O + 0.0273
Switzerland^25,29^	*δ*′^17^O = 0.5258 *δ*′^18^O to *δ*′^17^O = 0.5273 *δ*′^18^O	Vienna	*δ*′^17^O = 0.5259 *δ*′^18^O – 0.0015
Kolkata ^38^	*δ*′^17^O = 0.522 *δ*′^18^O + 0.015	Dhaka	*δ*′^17^O = 0.5270 *δ*′^18^O + 0.0069
Las Güixas cave, Pyrenees ^30^	*δ*′^17^O = 0.5246 *δ*′^18^O—0.0020	Cestas-Pierroton	*δ*′^17^O = 0.5256 *δ*′^18^O + 0.0031

The observed gradients of *λ*_lmwl_ outside of the tropics indicate an apparent increase pattern towards the poles, which observationally related to mean annual air temperature and mean annual *δ*^17^O or *δ*^18^O values, with *λ*_lmwl_ mainly falling between 0.526–0.528. We were unable to quantify this as significant and acknowledge that some stations classified as extratropical based on their long-term climatological information did have higher MAT values during the observation period than expected by their climate classification. In the polar regions, *γ*_lmwl_ was higher, contrasting with below average *Δ′*^17^O values (on a few observations). The relationship between *γ*_lmwl_ and MAT seemed to be tighter; however, given the large uncertainties associated with the samples analyzed from the Arctic regions, this question remains unanswered.

For the tropics, with *λ*_lmwl_ mainly ranging from 0.524–0.528, the search for a clear explanation remains as challenging as it is for tropical precipitation *δ*^18^O and *δ*^2^H values^39–41^ (see also SM table S9 and figure S4; few correlations were found). Though our dataset represents a significant number of year-round, monsoonal, and seasonally wet tropical GNIP stations, it remains to be seen whether improved temporal resolution of *Δ′*^17^O data in these areas could provide further or new insights.

Searching for non-advective MWLs—defining such as MWLs of sites and seasons where locally evaporated sources prevail, as opposed to air masses advected from a greater distance—we observed the lowest MWL *δ*^17^O/*δ*^18^O slopes (0.518–0.522) on the tropical islands during the dry season (Réunion, Ascension Island, or Galápagos). The closeness of these *λ*_mwl_ to the isotope fractionation factor for diffusive processes (*θ*_diff_ = 0.518)^20^ could be explained by two processes. First, these relatively high *δ-*values suggested that tropical island precipitation is sourced locally, i.e., from the sea immediately surrounding those locations with little overprinting by larger-scale hydroclimatic advective movements, like the Inter-tropical Convergence Zone (ITCZ) and related weather patterns. Second, lower overall seasonal RH could cause some sub-cloud evaporation. However, none of the usual indicators, like lowered *d*-excess were detected, and the *Δ′*^17^O values were not substantially lower. We acknowledge that sampling at tropical island locations are sometimes biased to the rainy season to capture the bulk of the precipitation isotopic signature, and hence fewer samples are obtained from the drier seasons. Also, monthly composite sampling resolution may be inadequate to disentangle locally vs distantly sourced precipitation at such island locations. One commonality is that the two sites with the lowest *λ*_mwl_ values are near upwelling zfones of cold ocean currents (Humboldt and Benguela); however, we lack any coherent explanation other than relatively stable atmospheric conditions. Higher resolution time-series information (e.g., event, daily) from tropical island locations are likely needed to address this question.

Our analysis left some open knowledge gaps. Our dataset had only six stations at altitudes greater than 2000 m.a.s.l. (four in the Americas and two in Africa). Only one of them (Quito/INAMHI, Ecuador, 2850 m.a.s.l.) had a sufficiently long precipitation record for detailed interpretation. The high-altitude GNIP station data generally showed a low mean *Δ*′^17^O and *γ*_mwl_ for the African stations, whereas the American stations followed a gradient opposite to *δ*′^18^O, as was expected from global distributions. Any *Δ*′^17^O evidence for a stratospheric-tropospheric exchange (STE^42^) and encroachment of stratospheric water vapor at higher altitudes thus remains speculative; for example, one STE hotspot over southern Greenland and Iceland corresponded with above-average *Δ′*^17^O values for Reykjavik from winter to spring. Similarly, winter *Δ′*^17^O in Kumamoto (Japan) was elevated; however, it was not commensurably elevated in Cheongju (Rep. of Korea), and competing processes could exist, such as marine evaporation under conditions of low RH under the influence of the Siberian high-pressure systems. Other STE hotspots over Argentina and Türkiye did not show *Δ′*^17^O excursions, possibly due to dry-season evaporative conditions overprinting that isotope signature.

As certified *δ*^17^O values for VSMOW2 and SLAP2 reference materials are lacking (see e.g., references ^2,20,22^); we urge caution that findings of this and prior works may be subject to revision or re-interpretation once the metrological aspects of *δ*^17^O are settled. Notwithstanding, we are confident that our baseline GNIP data and GMWL are internally consistent and will stimulate new *δ*^17^O and *Δ′*^17^O research and applications in hydrologic and climatic research across the globe. Additional data points in some targeted geographical areas will aid in improving our understanding of the role and behavior of *δ*^17^O and *Δ′*^17^O in the hydrological cycle. This recommendation applies particularly to (a) tropical island locations also in the dry/drier seasons, (b) high-latitude sites and (c) high-altitude locations. We call to access properly archived precipitation samples and to re-analyze them for *δ*^17^O and *Δ′*^17^O and to carefully preserve precipitation samples collected for *δ*^2^H, *δ*^18^O or ^3^H (i.e., evaporation prevention during sampling and storage) so that future *δ*^17^O and *Δ′*^17^O analyses may be feasible.

To conclude, our analysis of precipitation samples reaffirmed a GMWL *λ*_ref_ value of 0.528. Nonetheless, the line-conditioned *Δ*′^17^O_lc_ approach is a viable alternative if a nearby LMWL *λ* value is deemed more appropriate for local interpretations in hydrological and paleoclimatic studies.

## Methods

### Archived sample material and evaluation

Our samples consisted of n = 3484 GNIP precipitation samples archived at the IAEA Isotope Hydrology Laboratory and obtained from 88 stations worldwide, with 66 stations meeting allowing to characterize > 50% of the precipitation between 2015–2018 (of which 12 stations had 7–8 years record length; the remaining 22 stations had records shorter in duration or were beset with other limitations). We chose a four-year period following recent recommendations^15^, encompassing the GNIP samples collected between 2015 and 2018. The choice of GNIP stations for archived sample reanalysis was governed by data completeness, sample availability and sampling consistency, and by representativeness of the spatial and climatic distributions of stations. Eleven GNIP stations were analyzed for a longer extended period from 2015–2021 to test the appropriateness of the four-year criteria used in our approach. One station (Halley Bay, Antarctica) was an 8-year dataset spanning 2009–2016. This approach implied that archived samples were re-analyzed several years after their collection. A sample location map, colored according to the Köppen-Geiger synoptic climate classification^35^ is shown in Fig. 1. A table of the spatial and climatic characteristics of all GNIP sites is available in supplementary table S5. For five stations from the Canadian Arctic, which followed partially different pathways for sample collection, curation and analysis, these aspects are detailed in SM10. As a trade-off between that and the data paucity from polar regions, data from these samples were not used in the data analysis through they are shown, indicatively, on most of the figures.

Most archived samples had been stored in 30 mL brown glass bottles with polypropylene (PP) screw caps with conical inner liners (Etivera GmbH, St. Margarethen/Raab, Austria). The ability of these bottles to preserve the isotopic integrity of the samples over long periods has already been verified^43^. For several sites, samples were stored in high-density polyethylene (HDPE) or other plastic bottles, and the storage integrity was generally assessed positively but inferior to glass bottles (see supplementary materials, table S5 and figure S1); a finding in line with earlier research^44^. A minor fraction of the samples was archived in 1.5 mL screw-neck vials. Irrespective of the archiving bottle used, we conducted at least one assay for all samples identified as candidates (some analyses were abandoned if archived samples were isotopically compromised by evaporation based on *d*-excess or unacceptable enrichments in *δ*^18^O and *δ*^2^H values). The reproducibility criterion was the combined uncertainty of the present analysis, and a lumped long-term uncertainty of the control used a metric in the initial measurement of the sample (± 0.15 ‰ *δ*^18^O and ± 1.2‰ *δ*^2^H).

### Sample laboratory analysis

A detailed description of the analytical method for *δ*^17^O and *δ*^18^O analyses was published recently^45^; hence only a summary is provided. We used cavity ring-down spectroscopy (two 2140i CRDS analyzers, Picarro Inc.) with robotic autosamplers to measure up to 80 samples per week. Each GNIP sample was analyzed at least three times using six injections each, whereby the first injection was discarded from post-processing. Protocol design, memory and drift corrections were applied ^46,47^. All samples were normalized to the VSMOW-SLAP scale with the understanding that SLAP2 does not have a certified *δ*^17^O nor *Δ′*^17^O reference value, so we used the current consensus value ^48^. As daily-use reference materials, we used USGS48 and USGS46 with their *δ*^17^O and *Δ′*^17^O definitions ^49^. Data reduction used a Microsoft Access “Add-on” software^45^ for LIMS for Lasers^50^, which managed import, memory and drift corrections, and normalization to the VSMOW-SLAP scale using a two-point least-squares regression for *δ*^18^O, *δ*^17^O and *δ*^2^H simultaneously. The software computed the *d*-excess and *Δ′*^17^O with uncertainty propagation^45,51^. The calculation for the *Δ′*^17^O uncertainty is depicted in SM S2. Samples were repeated thrice or more if the standard deviation of repeated analyses was > 0.03 (*δ*^17^O), 0.06 (*δ*^18^O) or 0.02 ‰ (*Δ′*^17^O). The final value was calculated as the uncertainty-weighted mean of all accepted analyses; as the final uncertainty, the propagated uncertainty, or the standard deviation were reported, whichever of the two was higher. The long-term precision, expressed as the standard deviation of the control RM USGS45, was 0.050 ‰ (*δ*^18^O), 0.028 ‰ (*δ*^17^O), 0.009 ‰ (*Δ′*^17^O) and 0.3 ‰ for both *δ*^2^H and *d*-excess.

### Data treatment

We used the Köppen-Geiger ecozone primary classes (A—tropical, B—arid, C—temperate, D—cool, E—polar)^35^ to group the GNIP station datasets based on their mean temperatures and precipitation climatology. For all selected GNIP station datasets meeting our inclusion criteria (precipitation fraction covered with reproducible isotopic information > 50%, n = 66 stations) we calculated the weighted 2015–2018 mean *δ* and *Δ′* values as given in Eq. (2) in S1. The calculation of “weighted mean annual RH” follows the same schema. We then used the weighted station mean *δ*′^17^O and *δ*′^18^O values to derive a precipitation-based weighted *δ*′^17^O/*δ*′^18^O GMWL^52^, but also tested alternative approaches of deriving the GMWL (unweighted OLS and RMA techniques, forcing a zero intercept, or including sites that had failed the inclusion criterion). Correlation analysis and regression modelling were performed in R 4.3.0^53^, and all figures were created using the package “ggplot2”^54^.

We used our *δ*^17^O/*δ*^18^O dataset to calculate weighted LMWLs^14^ for the 66 locations across the globe based on four-year contiguous records for each. This record length followed recent expert recommendations on minimal LMWL sampling period lengths^15^ but was also constrained by available archival samples (going back to 2015) and the unavailability of more recent samples at the beginning of the measurement campaign. Our dataset allowed us to devise seasonal MWLs (DJF, MAM, JJA, SON) for those sample stations with extended 7–8 years of records. For those with a winter/summer seasonality, we chose the warmest and coldest trimesters and for sites whose seasonality is defined by rainy or dry periods, the driest and wettest trimesters.

### Sensitivity to sampling period length

We used the extended datasets (7–8 years, box symbols in Fig. 1) as a benchmark for the sensitivity of LMWL slope and intercept and weighted mean *δ*^17^O and *Δ′*^17^O against the record length. For every n = {1→7} years of record, we subsampled (n_max_-n + 1) consecutive intervals and computed their MWL and isotopic mean values. We related the average of results for each n to the range of results for n = 1, which represented the shortest possible annual records. Sensitivity tests for *δ*^17^O and *Δ′*^17^O, and also *λ*_mwl_ and *γ*_mwl_ are available from supplementary figure S2 and accompanying text. A summary assessment of the sample integrity is presented in figure S1; for individual stations the benchmark numbers are listed in table S5.

### Extraction of meteorological reanalysis data

We used both ERA-5^55^ and GPCC^56^ monthly resolved reanalysis data to complement missing observed precipitation data in the GNIP^9^ dataset. Where observed data were partially available for a given GNIP station, we tested them against both reanalysis datasets: If one of them correlated with the observed data with ≥ 75% variability explained, it was exclusively used to fill in the gaps. If either dataset explained < 75%, an ensemble of both was used (inverse weighted by correlation fit). A globally-mean weighted ensemble was used if there was no observed precipitation data. The final precipitation depth data were used to derive the weighted means and weighted meteoric water lines.

### Supplementary Information

Supplementary Information.

## Data Availability

All numerical data presented is available online from the IAEA WISER data portal https://nucleus.iaea.org/wiser and/or the IAEA Isotope Hydrology Collaboration Site https://nucleus.iaea.org/sites/ihn/Pages/Homepage.aspx.
