# Supplementary material for: Global and local meteoric water lines for δ17O/δ18O and the spatiotemporal distribution of Δ′17O in Earth’s precipitation
Source: Sci Rep. 2023 Nov 4;13:19056. doi: 10.1038/s41598-023-45920-8 (PMC10625609; doi:10.1038/s41598-023-45920-8)
Supplement: Supplementary file 1 — Supplementary Information. [file 41598_2023_45920_MOESM1_ESM.pdf]

# Global and local meteoric water lines for $\delta^{17}O/\delta^{18}O$ and the spatiotemporal distribution of $\Delta'^{17}O$ in Earth's precipitation

## Supplementary Materials

Stefan Terzer-Wassmuth[1]*, Luis J. Araguás-Araguás[1], Leonard I. Wassenaar[1,2], Christine Stumpp[3]

# S1 Relevant terms and equations

*Table S1: Glossary of relevant terms*

| **Term** | **Description** |
|---|---|
| $\delta$ | Ratio of rare over abundant isotope compared to reference standard, e.g., $\delta^{18}$O, $\delta^{17}$O, $\delta^{2}$H, see equation (1) |
| $\delta'$ | Delta prime: Log-normalized isotope ratio, e.g., $\delta'$17O or $\delta'$18O, see equation (7) |
| $\Delta'^{17}$O | $^{17}$O-excess ("cap delta $^{17}$O"), see equation (8) |
| $\Delta'^{17}O_{lc}$ | Line-conditioned $^{17}$O-excess, see main text for discussion. |
| $\lambda$ | Slope of a reference line, suffixes possible, see equation (6) and explanations |
| $\gamma$ | Intercept of a reference line, suffixes possible, see equation (6) and explanations |
| $\theta$ | Ratio of two fractionation factors, suffixes possible, see equation (9) and explanations |
| *d* | Deuterium excess; excess deuterium compared to a regression crossing through the origin, see equation (4) |
| | |
| MWL | Meteoric Water Line; regression of $\delta^{2}$H/$\delta^{18}$O or $\delta'^{17}$O/$\delta'^{18}$O. Global (GMWL), local (LMWL), and seasonal MWLs are used; so are weighted/unweighted. |
| OLS | Ordinary least squares; a regression technique |
| RMA | Reduced major axis; a regression technique |
| | |
| DJF | December-January-February; meteorological winter (temperate northern latitudes) |
| MAM | March-April-May; meteorological spring (temperate northern latitudes) |
| JJA | June-July-August; meteorological summer (temperate northern latitudes) |
| SON | September-October-November; meteorological fall (temperate northern latitudes) |
| ITCZ | Inner-tropical convergence zone, major atmospheric cycle governing rainy and dry seasons in the tropics |
| | |
| GNIP | Global Network of Isotopes in Precipitation |
| ERA-5 | Meteorological reanalysis dataset by ECMWF (European Centre for Medium-Range Weather Forecasts) |
| GPCC | Global Precipitation Climatology Centre, gridded precipitation dataset produced by the German Weather Service (DWD) |
| | |
| MAT | Mean annual temperature |
| MAP | Mean annual precipitation |
| MARH | Mean annual RH (relative humidity); weighted MARH in analogy to equation (2). |
| PPT | Precipitation (generic) |
| | |
| IRMS | Isotope ratio mass spectrometry |
| CRDS | Cavity ring-down spectroscopy (proprietary technology by Picarro Inc.) |
| OA-ICOS | Off-Axis Integrated Cavity Output Spectroscopy (proprietary technology by LGR Inc., now ABB Ltd.) |
| WVISS | Water Vapor Isotope Standard Source, water nebulization inlet accessory for OA-ICOS systems |

| Term | Description |
|---|---|
| RM | Reference material (used to normalize isotopic measurements to an international reference scale) |
| wRM | Working reference material (an “in-house standard” for normalizing isotopic measurements). |
| VSMOW2 | Vienna Standard Mean Ocean Water 2, certified primary high RM issued by IAEA. Note that there is only an assumed 0 value for $\delta^{17}$O. |
| SLAP2 | Standard Light Antarctic Precipitation 2, certified primary low RM issued by IAEA. Note that there are only literature values for $\delta^{17}$O. |
| VSMOW-SLAP scale | Reference scale to which $H_2O$ isotope measurements are normalized. Current implementation by VSMOW2 and SLAP2 (or other traceable RM). |
| USGS | United States Geological Survey. Issuer of certified RM USGS45, USGS46 and USGS48 used in this study. |

The natural abundance of $^{17}$O compared to $^{16}$O (0.0373 % in seawater) is commonly expressed as *δ* value in ‰ against the "zero water" VSMOW:

$$\delta = \frac{R_{sample}}{R_{reference}} - 1 \qquad \text{(Equation 1)}$$

where $R_{sample}$ is the sample’s $^{17}$O/$^{16}$O ratio, and $R_{reference}$ that of the RM[1].

The precipitation-weighted mean isotopic composition at a given location[2] is calculated as

$$\overline{\delta_w} = \frac{\sum \delta P}{\sum P} \qquad \text{(Equation 2)}$$

Where $\overline{\delta_w}$ represents the weighted mean, *δ* the delta value of an individual data point at a sampling station, and *P* its associated precipitation amount.

The MWL[3] is derived by (weighted) ordinary least squares regression of station data and is expressed as

$$\delta^2H = a_{mwl}\, \delta^{18}O + b_{mwl} \qquad \text{(Equation 3)}$$

where $a_{mwl}$ is the regression slope and $b_{mwl}$ the ordinate intercept. For the GMWL, weighted means of stations are used[2]. The deuterium excess[4] *d* is defined as

$$d = \delta^2H - a_{ref}\, \delta^{18}O \qquad \text{(Equation 4)}$$

where $a_{ref}$ = 8 is the $\delta^{18}$O/$\delta^2$H reference slope[3]. More modern definitions of the $\delta^{18}$O/$\delta^2$H GMWL exist with $a_{gmwl}$=8.2 and $b_{gmwl}$=11.2[2]; however, without replacing the initial $a_{ref}$ definition.

The so-called "line-conditioned *d*-excess" was developed primarily as a metric to normalize the deviations of d-excess in data from rivers to applicable LMWLs[5]:

$$lc\text{-excess} = \delta^2H - a_{lmwl} * \delta^{18}O - b_{lmwl}, \qquad \text{(Equation 5)}$$

where $a_{lmwl}$ is the slope of the LMWL and $b_{lmwl}$ is its intercept.

Similar to the above, the $\delta^{17}O/\delta^{18}O$ MWL[6] can be derived from the $\delta'$:

$$\delta'^{17}O = \lambda_{mwl}\, \delta'^{18}O + \gamma_{mwl} \qquad \text{(Equation 6)}$$

(Variants are regression lines using the $\lambda_{ref}$ slope and $\gamma_{ref}$ intercept; in analogy, $\lambda_{gmwl}$ and $\gamma_{gmwl}$ and $\lambda_{lmwl}$ and $\gamma_{lmwl}$ denote the slopes and intercepts of GMWL and LMWL, and $\lambda_{obs}$ and $\gamma_{obs}$ for any regression line fitted through a set of observed data[7].)

In those equations, the $\delta'$ ("delta prime")[8] is used, which is calculated as

$$\delta' = \ln(\delta+1) \times 1000 \qquad \text{(Equation 7)}$$

The "$^{17}O$ excess" is commonly expressed as[8]:

$$\Delta'^{17}O = \delta'^{17}O - \lambda_{ref} \times \delta'^{18}O \qquad \text{(Equation 8)}$$

The history of the $\lambda_{ref}$ reference slope is presented in chapter 1.2 of the main manuscript.

$\theta$ defines the relationship of the fractionation factors $^{17}\alpha$ and $^{18}\alpha$ for $^{17}O$ and $^{18}O$, respectively, and is calculated as[6]:

$$\theta = \ln(^{17}\alpha)/\ln(^{18}\alpha) \qquad \text{(Equation 9)}$$

A suffix denotes the process for which the αs are defined, e.g., $\theta_{diff}$ for diffusion processes[6].

## S2 Uncertainty of the $\Delta'^{17}O$

To calculate the $\Delta'^{17}O$ uncertainty, it was necessary to derive first the uncertainties of $\delta^{17}O$ and $\delta^{18}O$. Here, we considered that the main components of the overall uncertainty budget[9] of an isotopic measurement $u(\delta_s)$ are (i) the uncertainty of the measured sample $u(\delta_{sm})$, (ii) the uncertainty of the measured RM $u(\delta_{hm})$ and $u(\delta_{lm})$ for high and low RM respectively and (iii) the assigned uncertainty of the RM $u(\delta_h)$ and $u(\delta_l)$ for high and low RM respectively, hence uncertainties introduced by memory and drift correction were ignored. The uncertainty calculation for $\delta^{17}O$ and $\delta^{18}O$ followed that in SICalib[10,11] which is the source of the equations below.

$$u(\delta_s) = \sqrt{\left(\frac{\partial f}{\partial \delta_h}\right)^2 \cdot u(\delta_h)^2 + \left(\frac{\partial f}{\partial \delta_l}\right)^2 \cdot u(\delta_l)^2 + \left(\frac{\partial f}{\partial \delta_{hm}}\right)^2 \cdot u(\delta_{hm})^2 + \left(\frac{\partial f}{\partial \delta_{lm}}\right)^2 \cdot u(\delta_{lm})^2 + \left(\frac{\partial f}{\partial \delta_{sm}}\right)^2 \cdot u(\delta_{sm})^2}$$

(Equation 10)

$u(\delta_h)$ is the assigned uncertainty of the high RM. The sensitivity factor for $\delta_h$ is:

$$\left(\frac{\partial f}{\partial \delta_h}\right) = 1 - (\delta_{sm} - \delta_{hm})/(\delta_{lm} - \delta_{hm}) \qquad \text{(Equation 11)}$$

$u(\delta_l)$ is the assigned uncertainty of the low RM. The sensitivity factor for $\delta_l$ is:

$$\left(\frac{\partial f}{\partial \delta_l}\right) = 1 - (\delta_{sm} - \delta_{hm})/(\delta_{lm} - \delta_{hm}) \qquad \text{(Equation 12)}$$

$u(\delta_{hm})$ is the variance from uncertainty of the high standard, expressed as the standard error of the mean of 10 memory-corrected injections. The sensitivity factor for $\delta_{hm}$ is:

$$\left(\frac{\partial f}{\partial \delta_{hm}}\right) = (\delta_l - \delta_h) \cdot (\delta_{sm} - \delta_{hm})/(\delta_{lm} - \delta_{hm})^2 - (\delta_l - \delta_h)/(\delta_{lm} - \delta_{hm}) \qquad \text{(Eq. 13)}$$

$u(\delta_{lm})$ is the variance from uncertainty of the low standard, expressed as the standard error of the mean of 10 memory-corrected injections. The sensitivity factor for $\delta_{lm}$ is:

$$\left(\frac{\partial f}{\partial \delta_{lm}}\right) = -(\delta_l - \delta_h) \cdot (\delta_{sm} - \delta_{hm})/(\delta_{lm} - \delta_{hm})^2 \qquad \text{(Equation 14)}$$

$u(\delta_{sm})$ is the variance from uncertainty of the sample, expressed as the standard error of the mean of 5 memory-corrected injections. The sensitivity factor for $\delta_{sm}$ is:

$$\left(\frac{\partial f}{\partial \delta_{sm}}\right) = (\delta_l - \delta_h)/(\delta_{lm} - \delta_{hm}) \qquad \text{(Equation 15)}$$

The resulting $\delta_{hm}$, $\delta_{lm}$ and $\delta_{sm}$ are then penalized for the small number of observations by multiplication with Student's t (a table of factors is given in reference [12]).

Having established the $u(\delta_s)$ for both $\delta^{17}O$ and $\delta^{18}O$, the $u(\Delta'^{17}O)$ computes as follows (in per meg):

$$u(\Delta'^{17}O) = 1000 \cdot \sqrt{\left(\frac{\delta^{17}O}{1000} + 1\right)^{-2} \cdot u(\delta^{17}O)^2 + \left(\log\left(\frac{\delta^{18}O}{1000} + 1\right)\right)^2 \cdot 10^6 \cdot u(\lambda_{ref})^2 + \lambda_{ref}{}^2 \cdot \left(\frac{\delta^{18}O}{1000} + 1\right)^2 \cdot u(\delta^{18}O)^2}$$

(Equation 16)

Equation 16 uses the $\lambda_{ref}$ of 0.5279±0.0001 [6].

This computes the $\delta^{18}$O, $\delta^{17}$O and $\Delta'^{17}$O of a single analysis. Multiple analyses $\delta_{s1\rightarrow n}$ and their uncertainties u($\delta_{s1\rightarrow n}$) are then combined as uncertainty-weighted mean ($\delta_c$) and uncertainty-weighted standard deviation u($\delta_c$):

$$\delta_c = \frac{\sum_{i=1\rightarrow n} \delta_i/u(\delta_i)^2}{\sum_{i=1\rightarrow n} 1/u(\delta_i)^2} \quad \text{(Equation 17)}$$

$$u(\delta_c) = \sqrt{1/\sum_{i=1\rightarrow n} 1/u(\delta_i)^2} \quad \text{(Equation 18)}$$

The $\Delta'^{17}$O in this context is treated like a $\delta$ value. To account for the external precision, we furthermore compute the standard deviation of the n analyses and report that or u($\delta_c$), whichever is higher.

As an example, we present three measurements of USGS45 (known unknown) against USGS48 (high standard) and USGS46 (low standard). $\delta^{18}$O values are from the USGS RM certificates, $\delta^{17}$O and $\Delta'^{17}$O values from reference[13].

*Table S2: RMs used in this example:*

| RM | $\delta^{18}$O (‰) | $\delta^{17}$O (‰) | $\Delta'^{17}$O (‰) |
|---|---|---|---|
| USGS45 | -2.238 ± 0.011 | -1.19 ± 0.03 | 0.012 ± 0.001 |
| USGS46 | -29.80 ± 0.02 | -15.85 ± 0.02 | 0.020 ± 0.002 |
| USGS48 | -2.224 ± 0.012 | -1.15 ± 0.01 | 0.026 ± 0.003 |

*Table S3: Example of three analyses of USGS45:*

| | Sample | $\delta^{17}$O | u($\delta^{17}$O) | $\delta^{18}$O | u($\delta^{18}$O) | $\Delta'^{17}$O | u($\Delta'^{17}$O) |
|---|---|---|---|---|---|---|---|
| Analysis 1 | USGS48 | -1.151 | 0.022 | -2.225 | 0.032 | | |
| | USGS46 | -15.850 | 0.010 | -29.800 | 0.011 | | |
| | USGS45 | -1.175 | 0.025 | -2.257 | 0.037 | 0.017 | 0.032 |
| Analysis 2 | USGS48 | -1.151 | 0.022 | -2.225 | 0.032 | | |
| | USGS46 | -15.850 | 0.007 | -29.800 | 0.008 | | |
| | USGS45 | -1.178 | 0.025 | -2.258 | 0.037 | 0.014 | 0.032 |
| Analysis 3 | USGS48 | -1.150 | 0.022 | -2.224 | 0.032 | | |
| | USGS46 | -15.850 | 0.011 | -29.800 | 0.013 | | |
| | USGS45 | -1.192 | 0.025 | -2.270 | 0.037 | 0.007 | 0.032 |
| **$\delta_c$ and u($\delta_c$)** | **USGS45** | **-1.182** | **0.015** | **-2.262** | **0.021** | **0.013** | **0.018** |
| **SD** | | | **0.009** | | **0.007** | | **0.005** |

Our approach resulted in a long-term $\Delta'^{17}$O precision (expressed as standard deviation of the control samples) of 0.09 ‰ for both controls (USGS45 and an in-house RM); hence we are certain that our uncertainty estimation is reasonable. As for the fitness for purpose we refer the reader to our earlier work[14]; the typical uncertainty for triplicate analysis of a sample was 0.014 ‰; when there was a need for ≤ 0.01 ‰, up to six analyses had to be performed. (We wish to mention that the example in table S13 was analyzed on an older instrument; hence the higher uncertainty. This device was not substantially involved in the creation of the data presented.)

Alternative approaches – equation 26 in reference [15] but with $\delta'$ equations 31 therein, taking the SD of accepted memory-corrected CRDS injections for $\delta^{17}O$ and $\delta^{18}O$ as σ to estimate u($\delta_i$) for each analysis in Table S3 and calculating the u($\delta_c$) as given in Eq. 17 and 18 – would have resulted in a u($\delta_c$) of 0.010, 0.010 and 0.013 ‰ for $\delta^{17}O$, $\delta^{18}O$ and $\Delta'^{17}O$ respectively. However, this method made extensive use of $^{16}O$ isotope ratios which can be calculated from the those of the rare isotopes' $\delta$ values (the default CRDS output); yet the quantification of uncertainty is challenging and/or beset with assumptions. Also, the method is based on a single primary RM (which is not actually measured but two secondary ones). Finally, the method uses a different definition of the $\delta'$ than that established earlier.[7]

The most simplistic approach, calculating the u($\Delta'^{17}O$) by simple error propagation on Eq. 8, would result in values of 0.010 or 0.019 ‰, depending on whether the u($\delta_c$) or SD values for $\delta^{18}O$ and $\delta^{17}O$ from Table S3 were used. However, taking into consideration that the SD does not incorporate the assigned and measured uncertainties of the RMs, we feel that both SD-based approaches (the 0.010 ‰ as well as the 0.013 ‰ following the former method[15]) are rather ambitious estimates (not to mention using the SD of the three analyses, which would be as little as 0.005 ‰!), albeit in line with the long-term precision we reported earlier[14].

In this sense, the u($\Delta'^{17}O$) values stated by us (0.014 ‰ for triplicate analysis of a sample or greater than that, as in the example of Table S3) represent a more conservative approach and we consider our data fit for the purpose of a first assessment of this kind, even though – contrasting $\delta^{18}O$ and $\delta^{2}H$ variabilities, where precipitation shows the greatest variability of all elements of the water cycle – $\Delta'^{17}O$ seems to be of rather minor variability.

## S3 LMWLs from the literature

*Table S4: LMWLs from the literature*

| Location | Samples | Analyzer | Standards | Mean $\Delta'^{17}O$ | $\lambda_{mwl}$ | $\gamma_{mwl}$ | Comment |
|---|---|---|---|---|---|---|---|
| Zionsville, IN[16] | 235 daily | OA-ICOS with WVISS | LGR | 0.031 | 0.5273 | 0.0252 | |
| Windhoek [17] | 109 events | OA-ICOS with WVISS | LGR | 0.009 | 0.51 - 0.525 | n/a | Multiple yearly/ source MWLs |
| | | | | 0.014 | 0.5261 | 0.0036 | Recalculated from supplement (weighted) |
| Oozato, Okinawa[18] | 95 events | CRDS | wRM | 0.022 | 0.5304 | 0.0377 | Calculated from supplement (weighted) |
| China[19] | Tap waters | OA-ICOS with WVISS | LGR | 0.039 | 0.5281 - 0.5292 | 0.0485 | Seasonal/regional MWLs |
| Singapore[20] | Monthly 5y | CRDS | wRM | 0.022 | 0.5271 | 0.0154 | |
| Chicago[21] | 27 samples, 3y | IRMS | n/a | n/a | 0.529 | 0.071 | |
| Kolkata[22] | 61 events | OA-ICOS | LGR | 0.049 | 0.522 | 0.015 | Large $\Delta'^{17}O$ scatter (SD=0.171) |
| Southern Peru[7] | 10 sites, selected events | IRMS | VSMOW2, SLAP2 | 0.036 | 0.527 | n/a | Calculated from supplement |
| | | | | | 0.5275 | 0.031 | Calculated from supplement (weighted w/intercept) |
| Switzerland[23] | 7 sites, 300-3600 m.a.s.l. | CRDS | wRM | 0.0065-0.0167 | 0.5258 - 0.5273 | n/a | |
| Antarctic Surface Snow[24] | Vostok station | IRMS | n/a | 0.045 | 0.528 | 0.047 | |
| Mormont, Switzerland[25] | 274 daily, 2y | CRDS | wRM | 0.018 | 0.527 | 0.005 | |
| Las Güixas, Pyrenees[26] | 210 daily, 2 y | CRDS | wRM | 0.023 | 0.5246 | -0.0020 | |

# S4 Additional tables

*Table S5: List of GNIP stations analyzed in this study*

| Station [1)] | Country | Altitude | Latitude | Longitude | Precipitation | MAT | RH | Climate | PPT Src. | Sampling | n | Storage | analyzed | useful |
|---|---|---|---|---|---|---|---|---|---|---|---|---|---|---|
| | ISO-2 | m.a.s.l. | Decimal Degrees | | mm/mo. | °C | % | Köppen | [2)] | Period | | [3)] | % of precipitation | |
| **Halley Bay** | **AQ** | **30** | **-75.583** | **-20.567** | **40.0** | **-16.8** | **62.5** | **E** | **ERA5** | **2009-16** | **95** | **Glass** | **99.9%** | **89.6%** |
| Rothera Point | AQ | 5 | -67.570 | -68.130 | 36.7 | -5.7 | 77.1 | E | OBS | 2015-18 | 47 | Glass | 98.1% | 94.6% |
| Mar de Plata | AR | 10 | -38.000 | -57.550 | 86.7 | 14.9 | 77.6 | C | OBS | 2015-18 | 32 | Plastic* | 75.3% | 69.3% |
| *Santa Rosa* | *AR* | *180* | *-36.620* | *-64.600* | *70.2* | *15.8* | *62.4* | *C* | *GPCC* | *2015-18* | *30* | *Plastic** | *57.6%* | *48.0%* |
| **Vienna** | **AT** | **198** | **48.210** | **16.200** | **53.6** | **11.0** | **70.2** | **C** | **OBS** | **2015-21** | **82** | **Glass** | **97.8%** | **85.3%** |
| Dhaka | BD | 14 | 23.953 | 90.279 | 162.0 | 25.8 | 79.5 | A | OBS | 2015-18 | 42 | Glass | 100.0% | 94.9% |
| Ouagadougou | BF | 298 | 12.379 | -1.503 | 73.9 | 28.8 | 46.7 | B | OBS | 2015-18 | 16 | Glass | 72.0% | 58.5% |
| Cotonou | BJ | 14 | 6.417 | 2.329 | 96.5 | 26.8 | 84.5 | A | GLOB | 2015-18 | 41 | Glass | 98.7% | 90.6% |
| *Viacha* | *BO* | *3635* | *-16.499* | *-68.123* | *95.8* | *7.5* | *73.4* | *C* | *GLOB* | *2015-18* | *26* | *Plastic* | *58.4%* | *34.5%* |
| Belo Horizonte | BR | 857 | -19.871 | -43.967 | 111.8 | 20.1 | 73.5 | A | OBS | 2015-18 | 37 | Glass | 95.5% | 80.6% |
| Rio Claro | BR | 614 | -22.399 | -47.544 | 113.4 | 22.7 | 67.8 | A | ENS | 2015-18 | 40 | Glass | 95.0% | 71.5% |
| *Calgary* | *CA* | *1049* | *51.017* | *-114.017* | *36.8* | *4.6* | *61.1* | *D* | *OBS* | *2018-21* | *29* | *Plastic* | *60.0%* | *20.4%* |
| Charlottetown | CA | 49 | 46.289 | -63.119 | 97.3 | 6.7 | 77.6 | C | OBS | 2018-21 | 37 | Plastic* | 75.4% | 51.0% |
| Ottawa | CA | 114 | 45.320 | -75.670 | 79.5 | 7.1 | 70.1 | D | OBS | 2018-21 | 35 | Plastic* | 68.7% | 59.5% |
| Vancouver | CA | 4 | 49.195 | -123.017 | 103.4 | 11.5 | 75.8 | C | OBS | 2018-21 | 38 | Plastic* | 88.5% | 85.7% |
| Goma | CD | 1535 | -1.682 | 29.227 | 114.6 | 19.1 | 76.3 | A | ENS | 2015-18 | 27 | Glass | 62.6% | 54.7% |
| Kisangani | CD | 418 | 0.512 | 25.206 | 128.0 | 25.5 | 84.6 | A | OBS | 2015-18 | 47 | Glass | 99.9% | 84.9% |
| Bangui Univ. | CF | 363 | 4.377 | 18.562 | 106.8 | 26.4 | 73.3 | A | OBS | 2015-18 | 42 | Glass | 99.1% | 71.1% |
| **Isla de Pascua** | **CL** | **41** | **-27.170** | **-109.430** | **72.5** | **21.1** | **73.9** | **C** | **OBS** | **2015-21** | **77** | **Plastic*** | **87.3%** | **75.2%** |
| *Puerto Montt* | *CL* | *13* | *-41.470* | *-72.930* | *117.1* | *10.6* | *82.4* | *C* | *OBS* | *2015-18* | *45* | *Plastic** | *96.6%* | *49.9%* |
| Douala | CM | 18 | 4.037 | 9.734 | 289.3 | 26.6 | 87.9 | A | OBS | 2015-18 | 41 | Glass | 99.8% | 95.3% |
| **Hong Kong** | **CN** | **66** | **22.317** | **114.167** | **198.3** | **23.6** | **80.3** | **C** | **OBS** | **2015-21** | **81** | **Glass** | **100.0%** | **72.8%** |
| *Bogota* | *CO* | *2547* | *4.700* | *-74.130* | *76.7* | *13.2* | *80.8* | *C* | *ENS* | *2015-18* | *16* | *Glass* | *34.9%* | *26.1%* |
| Tulenapa | CO | 30 | 7.774 | -76.666 | 213.9 | 25.3 | 87.6 | A | OBS | 2015-18 | 38 | Glass | 85.8% | 77.0% |

| | | | | | | | | | | | | | | |
|---|---|---|---|---|---|---|---|---|---|---|---|---|---|---|
| *Heredia* | *CR* | *1150* | *9.930* | *-84.100* | *153.7* | *18.2* | *82.5* | *A* | *OBS* | *2015-18* | *19* | *Plastic** | *51.0%* | *30.2%* |
| Havana (CPHR) | CU | 137 | 23.050 | -82.217 | 132.1 | 25.3 | 73.0 | A | OBS | 2015-18 | 43 | Glass | 98.7% | 89.1% |
| Uhlirska | CZ | 823 | 50.833 | 15.148 | 88.1 | 7.7 | 79.0 | C | OBS | 2015-18 | 47 | Glass | 100.0% | 82.5% |
| Copenhagen | DK | 10 | 55.677 | 12.560 | 54.9 | 9.7 | 80.3 | C | GLOB | 2015-18 | 42 | Glass | 88.1% | 83.3% |
| ***Galapagos*** | ***EC*** | ***15*** | ***-0.897*** | ***-89.609*** | ***63.7*** | ***23.1*** | ***86.4*** | ***B*** | ***OBS*** | ***2015-21*** | ***25*** | ***Glass*** | ***41.9%*** | ***28.7%*** |
| Nueva Rocafuerte | EC | 185 | -0.933 | -75.400 | 260.8 | 25.6 | 88.6 | A | GPCC | 2015-18 | 45 | Glass | 93.3% | 86.7% |
| Quito (INAMHI) | EC | 2850 | -0.233 | -71.167 | 215.0 | 25.2 | 89.1 | C | ENS | 2015-18 | 44 | Glass | 82.6% | 72.4% |
| *Asmara* | *ER* | *2345* | *15.334* | *38.929* | *56.0* | *19.6* | *62.2* | *B* | *GLOB* | *2015-18* | *14* | *Glass* | *48.5%* | *28.6%* |
| Addis Ababa | ET | 2360 | 9.000 | 38.730 | 92.6 | 18.7 | 57.5 | C | ERA5 | 2015-18 | 25 | Glass | 57.1% | 50.8% |
| Espoo | FI | 30 | 60.180 | 24.833 | 54.6 | 6.9 | 82.6 | D | OBS | 2015-18 | 46 | Plastic* | 98.4% | 70.6% |
| Kuopio | FI | 116 | 62.892 | 27.625 | 57.7 | 4.6 | 81.8 | D | OBS | 2015-18 | 46 | Plastic* | 98.2% | 82.0% |
| Rovaniemi | FI | 107 | 66.497 | 25.755 | 48.8 | 2.2 | 81.6 | D | OBS | 2015-18 | 41 | Plastic* | 94.3% | 75.2% |
| Cestas-Pierroton | FR | 59 | 44.738 | -0.775 | 77.1 | 14.4 | 72.6 | C | OBS | 2015-18 | 44 | Glass | 94.4% | 67.7% |
| Libreville | GA | 69 | 0.315 | 9.442 | 235.3 | 26.4 | 85.1 | A | GLOB | 2015-18 | 41 | Glass | 98.4% | 69.9% |
| Tbilisi | GE | 427 | 41.750 | 44.767 | 40.3 | 14.3 | 59.6 | C | GPCC | 2015-18 | 41 | Glass | 94.7% | 81.5% |
| Gibraltar | GI | 5 | 36.150 | -5.350 | 55.3 | 18.0 | 80.6 | C | GPCC | 2015-18 | 25 | Glass | 79.1% | 59.1% |
| **Danmarkshavn** | **GL** | **12** | **76.767** | **-18.667** | **23.0** | **-11.1** | **76.2** | **E** | **GPCC** | **2015-21** | **70** | **Glass** | **89.2%** | **78.0%** |
| Patras | GR | 100 | 38.280 | 21.790 | 66.5 | 14.9 | 67.1 | C | OBS | 2015-18 | 40 | Glass | 92.9% | 80.9% |
| *Thessaloniki* | *GR* | *200* | *40.631* | *22.957* | *46.2* | *16.4* | *69.5* | *C* | *ENS* | *2015-18* | *23* | *Glass* | *45.0%* | *38.1%* |
| **Valentia** | **IE** | **9** | **51.930** | **-10.250** | **142.0** | **10.6** | **83.1** | **C** | **OBS** | **2015-21** | **84** | **Glass** | **100.0%** | **85.8%** |
| **Reykjavik** | **IS** | **14** | **64.130** | **-21.930** | **76.0** | **4.8** | **80.2** | **C** | **OBS** | **2015-21** | **84** | **Glass** | **100.0%** | **91.2%** |
| Ancona | IT | 170 | 43.587 | 13.515 | 74.6 | 16.7 | 73.2 | C | OBS | 2015-18 | 41 | Glass | 91.9% | 72.5% |
| *Piano Marra* | *IT* | *83* | *40.269* | *15.147* | *82.5* | *15.1* | *74.4* | *C* | *ENS* | *2015-18* | *26* | *Glass* | *53.2%* | *37.9%* |
| Kumamoto | JP | 10 | 32.803 | 130.708 | 166.5 | 16.6 | 73.5 | C | OBS | 2015-18 | 45 | Glass | 96.5% | 78.9% |
| Cheongju | KR | 62 | 36.620 | 127.460 | 88.4 | 13.5 | 62.5 | C | GPCC | 2015-18 | 40 | Glass | 87.3% | 84.1% |
| Wellampitiya | LK | 5 | 6.951 | 79.880 | 210.6 | 27.2 | 82.7 | A | GPCC | 2015-18 | 48 | Glass | 100.0% | 93.8% |
| Fes Saïss | MA | 571 | 33.967 | -4.983 | 46.5 | 16.3 | 55.6 | C | GPCC | 2015-18 | 40 | Glass | 93.4% | 87.8% |
| Monaco | MC | 2 | 43.732 | 7.424 | 46.6 | 17.5 | 72.0 | C | ENS | 2015-18 | 45 | Glass | 99.9% | 92.0% |
| Leova | MD | 156 | 46.497 | 28.300 | 40.3 | 12.1 | 67.7 | C | ENS | 2015-18 | 47 | Glass | 92.3% | 68.2% |
| Antananarivo | MG | 1300 | -18.900 | 47.530 | 102.5 | 17.6 | 79.2 | A | OBS | 2015-18 | 31 | Glass | 100.0% | 80.0% |
| Bamako | ML | 381 | 12.693 | -7.995 | 78.5 | 28.5 | 48.7 | A | GPCC | 2015-18 | 14 | Glass | 79.8% | 53.2% |

| | | | | | | | | | | | | | | |
|---|---|---|---|---|---|---|---|---|---|---|---|---|---|---|
| Mexico City | MX | 2700 | 19.433 | -99.133 | 106.6 | 14.4 | 65.2 | C | GLOB | 2015-18 | 38 | Plastic | 94.7% | 57.5% |
| *Xalapa* | *MX* | *1390* | *19.530* | *-96.910* | *131.6* | *21.4* | *79.5* | *A* | *GPCC* | *2015-18* | *36* | *Plastic* | *75.9%* | *45.9%* |
| Cameron Highlands | MY | 1430 | 4.467 | 101.383 | 222.1 | 23.6 | 84.2 | C | ENS | 2015-18 | 48 | Plastic | 100.0% | 59.2% |
| Johor Baharu | MY | 32 | 1.456 | 103.761 | 198.3 | 27.3 | 81.7 | A | ENS | 2015-18 | 44 | Plastic | 89.7% | 63.6% |
| *Kota Kinabalu* | *MY* | *9* | *5.930* | *116.060* | *222.3* | *23.4* | *86.7* | *A* | *ENS* | *2015-18* | *46* | *Plastic* | *100.0%* | *48.4%* |
| *Gorongosa* | *MZ* | *350* | *-18.679* | *34.071* | *84.3* | *24.2* | *71.0* | *A* | *GLOB* | *2015-18* | *22* | *Glass* | *45.8%* | *40.1%* |
| Niamey | NE | 220 | 13.520 | 2.090 | 47.7 | 29.5 | 39.3 | B | GPCC | 2015-18 | 25 | Glass | 99.5% | 95.2% |
| Diliman Quezon City | PH | 42 | 14.640 | 121.040 | 232.7 | 26.5 | 81.0 | A | OBS | 2015-18 | 38 | Glass | 99.8% | 72.8% |
| **Ponta Delgada** | **PT** | **175** | **37.770** | **-25.650** | **67.9** | **17.5** | **77.7** | **C** | **OBS** | **2015-21** | **83** | **Glass** | **99.9%** | **90.5%** |
| **Réunion** | **RE** | **70** | **-20.901** | **55.484** | **77.5** | **21.3** | **79.5** | **A** | **ENS** | **2015-21** | **32** | **Glass** | **66.4%** | **52.1%** |
| Ramnicu Valcea | RO | 237 | 45.035 | 24.284 | 59.3 | 10.8 | 75.0 | C | OBS | 2015-18 | 47 | Glass | 100.0% | 86.4% |
| Ny Ålesund | SB | 7 | 78.917 | 11.933 | 54.6 | -3.5 | 78.1 | E | OBS | 2015-18 | 47 | Glass | 98.9% | 89.4% |
| **Ascension** | **SH** | **15** | **-7.920** | **-14.420** | **13.1** | **25.0** | **76.2** | **B** | **OBS** | **2015-21** | **80** | **Glass** | **99.2%** | **75.5%** |
| **Travellers Hill** | **SH** | **400** | **-7.940** | **-14.373** | **38.8** | **25.0** | **76.3** | **B** | **OBS** | **2015-21** | **83** | **Glass** | **99.7%** | **84.9%** |
| N'Djamena | TD | 300 | 12.130 | 15.030 | 48.5 | 29.2 | 39.4 | B | OBS | 2015-18 | 21 | Glass | 91.8% | 85.3% |
| Bangkok | TH | 2 | 13.730 | 100.500 | 177.5 | 28.7 | 75.3 | A | GPCC | 2015-18 | 25 | Glass | 62.4% | 54.2% |
| Adana | TR | 73 | 36.980 | 35.300 | 58.5 | 20.4 | 69.6 | C | GPCC | 2015-18 | 36 | Glass | 94.0% | 74.8% |
| Ankara | TR | 902 | 39.950 | 32.880 | 38.7 | 12.1 | 56.5 | C | GPCC | 2015-18 | 47 | Glass | 100.0% | 77.4% |
| Antalya | TR | 49 | 36.880 | 30.700 | 67.3 | 19.7 | 65.4 | C | GPCC | 2015-18 | 41 | Glass | 100.0% | 78.0% |
| Rize | TR | 136 | 41.024 | 40.520 | 202.7 | 11.5 | 78.0 | C | GPCC | 2015-18 | 48 | Glass | 100.0% | 96.0% |
| Kharkiv | UA | 148 | 49.933 | 36.283 | 45.3 | 9.2 | 69.4 | D | GPCC | 2015-18 | 33 | Glass | 68.7% | 58.1% |
| Entebbe | UG | 1155 | 0.050 | 32.450 | 138.3 | 23.5 | 74.7 | A | OBS | 2015-18 | 43 | Glass | 88.8% | 72.0% |
| Armagh | UK | 64 | 54.353 | -6.648 | 72.5 | 9.8 | 82.6 | C | GPCC | 2015-18 | 43 | Glass | 91.3% | 82.7% |
| *Wallingford* | *UK* | *48* | *51.600* | *-1.100* | *48.4* | *10.9* | *79.3* | *C* | *OBS* | *2015-18* | *12* | *Glass* | *24.7%* | *22.3%* |
| Montevideo | UY | 37 | -34.904 | -56.167 | 83.2 | 17.1 | 77.6 | C | GLOB | 2015-18 | 42 | Glass | 79.0% | 65.7% |
| Hanoi | VN | 11 | 21.030 | 105.840 | 138.3 | 24.8 | 78.2 | C | OBS | 2015-18 | 46 | Glass | 99.7% | 85.6% |

1) Bold = analyzed for extended period, italics = not included in global calculations
2) Precipitation data source: OBS – Observed, ERA5 – ERA-5 reanalysis, GPCC – Global Precipitation Climatology Center, GLOB – ERA-5/GPCC globally weighted, ENS – Gaps in observed data filled by one of the above models.
3) Stations marked with * had aliquots taken from HDPE bottles initially submitted for tritium analysis

*Table S6: Summary of the isotopic characteristics of the GNIP stations chosen.*

| Station [1)] | Country | Altitude | Latitude | Longitude | $\delta^{17}O$ | $\delta^{18}O$ | $\delta^{2}H$ | d-excess | $\Delta'^{17}O$ | $\lambda_{lmwl}$ | $\gamma_{lmwl}$ | $\lambda_{lmwl}$ |
|---|---|---|---|---|---|---|---|---|---|---|---|---|
| | ISO-2 | m.a.s.l. | Decimal Degrees | | Weighted mean in ‰$_{VSMOW-SLAP}$ | | | | | weighted | | Wt., $\gamma$=0 |
| **Halley Bay** | **AQ** | **30** | **-75.583** | **-20.567** | **-12.655** | **-23.832** | **-185.6** | **5.0** | **0.002** | **0.5289** | **0.0236** | **0.5279** |
| Rothera Point | AQ | 5 | -67.570 | -68.130 | -7.530 | -14.246 | -111.6 | 2.4 | 0.018 | 0.5276 | 0.0121 | 0.5268 |
| Mar de Plata | AR | 10 | -38.000 | -57.550 | -2.832 | -5.394 | -30.4 | 12.7 | 0.020 | 0.5265 | 0.0114 | 0.5245 |
| *Santa Rosa* | *AR* | *180* | *-36.620* | *-64.600* | *-3.367* | *-6.397* | *-37.0* | *14.2* | *0.017* | *0.5271* | *0.0114* | *0.5256* |
| **Vienna** | **AT** | **198** | **48.210** | **16.200** | **-4.485** | **-8.506** | **-61.8** | **6.3** | **0.017** | **0.5259** | **-0.0015** | **0.5260** |
| Dhaka | BD | 14 | 23.953 | 90.279 | -2.976 | -5.651 | -34.0 | 11.3 | 0.013 | 0.5270 | 0.0069 | 0.5260 |
| Ouagadougou | BF | 298 | 12.379 | -1.503 | -2.409 | -4.592 | -26.8 | 9.9 | 0.018 | 0.5298 | 0.0262 | 0.5243 |
| Cotonou | BJ | 14 | 6.417 | 2.329 | -1.614 | -3.081 | -12.5 | 12.2 | 0.015 | 0.5255 | 0.0067 | 0.5236 |
| *Viacha* | *BO* | *3635* | *-16.499* | *-68.123* | *-6.788* | *-12.859* | *-89.6* | *13.3* | *0.024* | *0.5282* | *0.0273* | *0.5263* |
| Belo Horizonte | BR | 857 | -19.871 | -43.967 | -2.723 | -5.201 | -28.9 | 12.7 | 0.024 | 0.5262 | 0.0176 | 0.5234 |
| Rio Claro | BR | 614 | -22.399 | -47.544 | -2.363 | -4.521 | -23.6 | 12.6 | 0.026 | 0.5263 | 0.0198 | 0.5230 |
| *Calgary* | *CA* | *1049* | *51.017* | *-114.017* | *-8.811* | *-16.636* | *-139.0* | *-5.9* | *0.008* | *0.5259* | *-0.0265* | *0.5274* |
| Charlottetown | CA | 49 | 46.289 | -63.119 | -4.789 | -9.0942 | -58.6 | 14.1 | 0.023 | 0.5278 | 0.0215 | 0.5255 |
| Ottawa | CA | 114 | 45.320 | -75.670 | -5.935 | -11.247 | -77.0 | 12.9 | 0.020 | 0.5277 | 0.0174 | 0.5263 |
| Vancouver | CA | 4 | 49.195 | -123.017 | -5.591 | -10.601 | -74.7 | 10.1 | 0.021 | 0.5274 | 0.0142 | 0.5260 |
| Goma | CD | 1535 | -1.682 | 29.227 | -0.854 | -1.640 | 2.1 | 15.2 | 0.012 | 0.5285 | 0.0133 | 0.5240 |
| Kisangani | CD | 418 | 0.512 | 25.206 | -1.167 | -2.242 | -3.1 | 14.9 | 0.018 | 0.5277 | 0.0174 | 0.5235 |
| Bangui Univ. | CF | 363 | 4.377 | 18.562 | -1.059 | -2.034 | -2.5 | 13.8 | 0.016 | 0.5267 | 0.0132 | 0.5238 |
| **Isla de Pascua** | **CL** | **41** | **-27.170** | **-109.430** | **-1.371** | **-2.626** | **-8.2** | **12.8** | **0.016** | **0.5240** | **0.0058** | **0.5221** |
| *Puerto Montt* | *CL* | *13* | *-41.470* | *-72.930* | *-2.903* | *-5.520* | *-37.4* | *6.8* | *0.016* | *0.5239* | *-0.0064* | *0.5249* |
| Douala | CM | 18 | 4.037 | 9.734 | -1.238 | -2.372 | -9.1 | 9.8 | 0.015 | 0.5272 | 0.0136 | 0.5227 |
| **Hong Kong** | **CN** | **66** | **22.317** | **114.167** | **-3.299** | **-6.261** | **-38.6** | **11.5** | **0.012** | **0.5298** | **0.0235** | **0.5264** |
| *Bogota* | *CO* | *2547* | *4.700* | *-74.130* | *-4.969* | *-9.417* | *-65.3* | *10.1* | *0.016* | *0.5278* | *0.0135* | *0.5265* |
| Tulenapa | CO | 30 | 7.774 | -76.666 | -3.756 | -7.133 | -46.9 | 10.1 | 0.017 | 0.5282 | 0.0185 | 0.5259 |
| *Heredia* | *CR* | *1150* | *9.930* | *-84.100* | *-4.948* | *-9.376* | *-63.8* | *11.2* | *0.014* | *0.5295* | *0.0278* | *0.5267* |
| Havana (CPHR) | CU | 137 | 23.050 | -82.217 | -0.900 | -1.728 | -7.7 | 6.2 | 0.013 | 0.5269 | 0.0112 | 0.5238 |
| Uhlirska | CZ | 823 | 50.833 | 15.148 | -5.272 | -9.990 | -67.7 | 12.2 | 0.016 | 0.5266 | 0.0019 | 0.5264 |

| | | | | | | | | | | | | |
|---|---|---|---|---|---|---|---|---|---|---|---|---|
| Copenhagen | DK | 10 | 55.677 | 12.560 | -4.862 | -9.230 | -64.4 | 9.5 | 0.023 | 0.5268 | 0.0123 | 0.5256 |
| *Galapagos* | *EC* | *15* | *-0.897* | *-89.609* | *-0.537* | *-1.029* | *-0.4* | *7.8* | *0.006* | *0.5230* | *0.0009* | *0.5226* |
| Nueva Rocafuerte | EC | 185 | -0.933 | -75.400 | -2.621 | -4.995 | -28.2 | 11.7 | 0.020 | 0.5270 | 0.0156 | 0.5246 |
| Quito (INAMHI) | EC | 2850 | -0.233 | -71.167 | -5.898 | -11.170 | -79.6 | 9.8 | 0.017 | 0.5285 | 0.0233 | 0.5267 |
| *Asmara* | *ER* | *2345* | *15.334* | *38.929* | *-1.088* | *-2.077* | *-0.2* | *16.4* | *0.009* | *0.5231* | *-0.0013* | *0.5235* |
| Addis Ababa | ET | 2360 | 9.000 | 38.730 | -0.283 | -0.550 | 8.7 | 13.1 | 0.008 | 0.5259 | 0.0063 | 0.5251 |
| Espoo | FI | 30 | 60.180 | 24.833 | -5.716 | -10.829 | -77.2 | 9.5 | 0.017 | 0.5271 | 0.0077 | 0.5264 |
| Kuopio | FI | 116 | 62.892 | 27.625 | -6.583 | -12.468 | -90.6 | 9.2 | 0.021 | 0.5272 | 0.0102 | 0.5264 |
| Rovaniemi | FI | 107 | 66.497 | 25.755 | -6.861 | -12.989 | -96.1 | 7.8 | 0.019 | 0.5277 | 0.0143 | 0.5266 |
| Cestas-Pierroton | FR | 59 | 44.738 | -0.775 | -2.922 | -5.558 | -33.6 | 10.9 | 0.016 | 0.5256 | 0.0031 | 0.5251 |
| Libreville | GA | 69 | 0.315 | 9.442 | -1.355 | -2.598 | -6.7 | 14.1 | 0.018 | 0.5267 | 0.0143 | 0.5221 |
| Tbilisi | GE | 427 | 41.750 | 44.767 | -2.986 | -5.658 | -35.0 | 10.3 | 0.007 | 0.5265 | -0.0018 | 0.5267 |
| Gibraltar | GI | 5 | 36.150 | -5.350 | -2.217 | -4.232 | -23.1 | 10.8 | 0.020 | 0.5255 | 0.0094 | 0.5235 |
| **Danmarkshavn** | **GL** | **12** | **76.767** | **-18.667** | **-9.001** | **-16.994** | **-128.1** | **7.9** | **0.009** | **0.5289** | **0.0243** | **0.5275** |
| Patras | GR | 100 | 38.280 | 21.790 | -2.913 | -5.545 | -30.8 | 13.6 | 0.019 | 0.5249 | 0.0017 | 0.5246 |
| *Thessaloniki* | *GR* | *200* | *40.631* | *22.957* | *-2.906* | *-5.521* | *-35.9* | *8.3* | *0.014* | *0.5233* | *-0.0125* | *0.5253* |
| **Valentia** | **IE** | **9** | **51.930** | **-10.250** | **-2.689** | **-5.122** | **-31.7** | **9.3** | **0.020** | **0.5244** | **0.0006** | **0.5243** |
| **Reykjavik** | **IS** | **14** | **64.130** | **-21.930** | **-4.713** | **-8.941** | **-63.4** | **8.1** | **0.019** | **0.5272** | **0.0113** | **0.5260** |
| Ancona | IT | 170 | 43.587 | 13.515 | -3.284 | -6.249 | -38.1 | 11.9 | 0.021 | 0.5262 | 0.0095 | 0.5249 |
| *Piano Marra* | *IT* | *83* | *40.269* | *15.147* | *-2.750* | *-5.236* | *-28.7* | *13.2* | *0.019* | *0.5246* | *0.0012* | *0.5244* |
| Kumamoto | JP | 10 | 32.803 | 130.708 | -3.755 | -7.138 | -46.2 | 10.9 | 0.021 | 0.5295 | 0.0322 | 0.5253 |
| Cheongju | KR | 62 | 36.620 | 127.460 | -4.253 | -8.074 | -53.2 | 11.4 | 0.019 | 0.5283 | 0.0216 | 0.5259 |
| Wellampitiya | LK | 5 | 6.951 | 79.880 | -2.655 | -5.049 | -27.7 | 12.7 | 0.014 | 0.5277 | 0.0131 | 0.5256 |
| Fes Saïss | MA | 571 | 33.967 | -4.983 | -2.618 | -4.974 | -28.2 | 11.6 | 0.012 | 0.5256 | 0.0002 | 0.5256 |
| Monaco | MC | 2 | 43.732 | 7.424 | -2.895 | -5.503 | -34.3 | 9.7 | 0.014 | 0.5264 | 0.0058 | 0.5255 |
| Leova | MD | 156 | 46.497 | 28.300 | -4.646 | -8.807 | -60.7 | 9.7 | 0.015 | 0.5272 | 0.0078 | 0.5264 |
| Antananarivo | MG | 1300 | -18.900 | 47.530 | -4.028 | -7.644 | -47.6 | 13.6 | 0.017 | 0.5280 | 0.0176 | 0.5262 |
| Bamako | ML | 381 | 12.693 | -7.995 | -2.399 | -4.560 | -28.7 | 7.8 | 0.011 | 0.5260 | 0.0019 | 0.5256 |
| Mexico City | MX | 2700 | 19.433 | -99.133 | -4.210 | -7.970 | -53.2 | 10.5 | 0.007 | 0.5275 | 0.0033 | 0.5272 |
| *Xalapa* | *MX* | *1390* | *19.530* | *-96.910* | *-2.347* | *-4.466* | *-22.8* | *12.9* | *0.014* | *0.5282* | *0.0151* | *0.5254* |
| Cameron Highlands | MY | 1430 | 4.467 | 101.383 | -4.513 | -8.556 | -54.7 | 13.7 | 0.014 | 0.5269 | 0.0040 | 0.5264 |

| | | | | | | | | | | | | |
|---|---|---|---|---|---|---|---|---|---|---|---|---|
| Johor Baharu | MY | 32 | 1.456 | 103.761 | -3.205 | -6.082 | -39.4 | 9.2 | 0.011 | 0.5262 | 0.0001 | 0.5262 |
| *Kota Kinabalu* | *MY* | *9* | *5.930* | *116.060* | *-2.878* | *-5.468* | *-34.0* | *9.7* | *0.013* | *0.5279* | *0.0130* | *0.5259* |
| *Gorongosa* | *MZ* | *350* | *-18.679* | *34.071* | *-1.324* | *-2.524* | *-8.1* | *12.1* | *0.010* | *0.5271* | *0.0074* | *0.5251* |
| Niamey | NE | 220 | 13.520 | 2.090 | -1.786 | -3.412 | -17.3 | 10.0 | 0.018 | 0.5261 | 0.0110 | 0.5236 |
| Diliman Quezon City | PH | 42 | 14.640 | 121.040 | -2.998 | -5.682 | -37.8 | 7.7 | 0.007 | 0.5270 | 0.0009 | 0.5269 |
| **Ponta Delgada** | **PT** | **175** | **37.770** | **-25.650** | **-1.723** | **-3.304** | **-15.0** | **11.4** | **0.023** | **0.5269** | **0.0191** | **0.5217** |
| **Réunion** | **RE** | **70** | **-20.901** | **55.484** | **-1.388** | **-2.640** | **-11.2** | **9.9** | **0.008** | **0.5268** | **0.0045** | **0.5256** |
| Ramnicu Valcea | RO | 237 | 45.035 | 24.284 | -4.314 | -8.190 | -56.4 | 9.1 | 0.020 | 0.5273 | 0.0147 | 0.5258 |
| Ny Ålesund | SB | 7 | 78.917 | 11.933 | -5.361 | -10.150 | -75.0 | 6.2 | 0.012 | 0.5279 | 0.0105 | 0.5269 |
| **Ascension** | **SH** | **15** | **-7.920** | **-14.420** | **-0.028** | **-0.077** | **10.2** | **10.8** | **0.012** | **0.5216** | **0.0123** | **0.5140** |
| **Travellers Hill** | **SH** | **400** | **-7.940** | **-14.373** | **-0.117** | **-0.245** | **9.8** | **11.7** | **0.012** | **0.5232** | **0.0113** | **0.5075** |
| N'Djamena | TD | 300 | 12.130 | 15.030 | -1.835 | -3.494 | -18.3 | 9.7 | 0.011 | 0.5262 | 0.0053 | 0.5250 |
| Bangkok | TH | 2 | 13.730 | 100.500 | -2.366 | -4.488 | -27.2 | 8.7 | 0.007 | 0.5264 | 0.0000 | 0.5264 |
| Adana | TR | 73 | 36.980 | 35.300 | -2.459 | -4.686 | -22.0 | 15.5 | 0.018 | 0.5223 | -0.0088 | 0.5240 |
| Ankara | TR | 902 | 39.950 | 32.880 | -3.929 | -7.451 | -47.9 | 11.7 | 0.013 | 0.5254 | -0.0061 | 0.5261 |
| Antalya | TR | 49 | 36.880 | 30.700 | -2.638 | -5.029 | -23.9 | 16.3 | 0.021 | 0.5259 | 0.0102 | 0.5242 |
| Rize | TR | 136 | 41.024 | 40.520 | -4.329 | -8.217 | -49.5 | 16.2 | 0.019 | 0.5270 | 0.0109 | 0.5258 |
| Kharkiv | UA | 148 | 49.933 | 36.283 | -4.072 | -7.716 | -57.0 | 4.7 | 0.011 | 0.5249 | -0.0127 | 0.5262 |
| Entebbe | UG | 1155 | 0.050 | 32.450 | -1.198 | -2.294 | -4.3 | 14.1 | 0.014 | 0.5261 | 0.0092 | 0.5234 |
| Armagh | UK | 64 | 54.353 | -6.648 | -4.174 | -7.922 | -53.7 | 9.7 | 0.016 | 0.5250 | -0.0079 | 0.5259 |
| *Wallingford* | *UK* | *48* | *51.600* | *-1.100* | *-3.780* | *-7.192* | *-47.8* | *9.8* | *0.024* | *0.5277* | *0.0222* | *0.5247* |
| Montevideo | UY | 37 | -34.904 | -56.167 | -2.652 | -5.062 | -27.2 | 13.3 | 0.024 | 0.5284 | 0.0260 | 0.5235 |
| Hanoi | VN | 11 | 21.030 | 105.840 | -3.926 | -7.441 | -49.5 | 10.0 | 0.011 | 0.5281 | 0.0116 | 0.5268 |

1) Bold = analyzed for extended period, italics = not included in global calculations.

# S5 Sensitivity and reproducibility analysis plots

Our assessment of the reproducibility of an archived sample's isotopic composition (as specified in the "Methods" section) resulted in a "fraction of useful data" (i.e., the fraction of precipitation during the observation period covered by reproducible information based on $\delta^{18}$O and $\delta^{2}$H; see Table S5). It is worth noting that this benchmark relates the reproducible isotopic information (in mm precipitation) to the total precipitation during the observation period and the unavailability of sample material (due to sampling gaps or rare occurrences of samples lost after the initial analysis) induced an *a priori* reduction of this value.

Figure S1 summarizes the fraction of reproducible isotopic values for the samples available, aggregated per station and grouped into those using brown glass bottles and those who, wholly or in part, used plastic bottles.

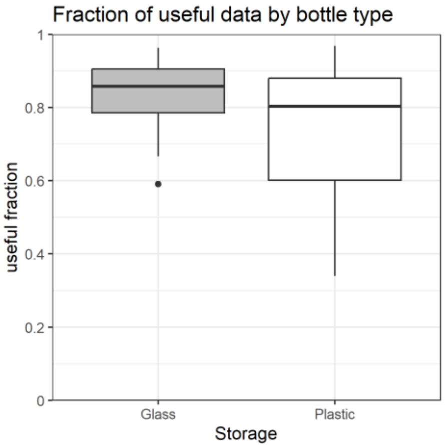


*Figure S1: Median fraction of reproducible data per station, grouped by usage of glass and plastic sample bottles*

Our sensitivity simulations assessed the minimum time-series criteria for establishing robust MWLs (see figure S2). Using the results of $\delta^{17}$O/$\delta^{18}$O measurements for the nine GNIP stations with an extended seven-year data period, we determined that for the LMWL slope ($\lambda_{lmwl}$), intercept ($\gamma_{mwl}$) and mean $\delta^{18}$O, an observational period of at least four years resulted in a minimized residual span of values of on average ca. 25% (spanning 15-50%) compared to a 1-year simulation. The minimized residual span of the mean $\Delta'^{17}$O were slightly higher (spanning 25-60%). This analysis suggested a minimum of four years of precipitation data is suitable for most locations. However, six years may be desirable for more robust LMWL intercepts and mean $\Delta'^{17}$O, especially for stations with a narrow range of $\delta$ values in precipitation, like Ascension Island (ca. 3 ‰ $\delta^{17}$O). The finding of a minimum of 4-6 years for robust foundational LMWL datasets is consistent with similar recommendations and observations[27] proposed previously for $\delta^{18}$O and $\delta^{2}$H.

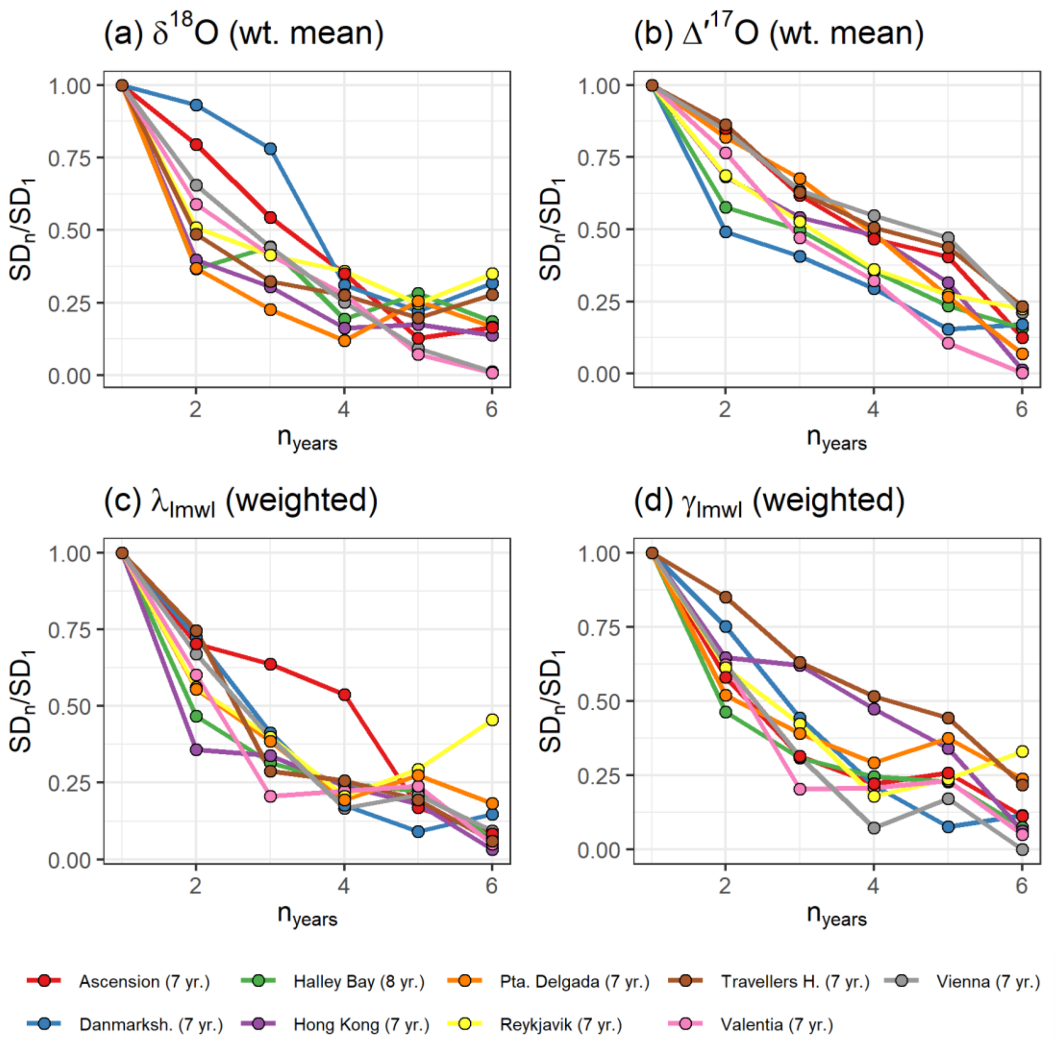


*Figure S2: Sensitivity tests of GNIP stations LMWLs comparing 1 to 6-year dataset collections as a function of the ratio of the summed yearly standard deviations for $\delta^{18}O$, $\Delta'^{17}O$, slopes ($\lambda_{lmwl}$) and intercepts ($\gamma_{lmwl}$).*

# S6 Additional MWL analyses

*Table S7: mean $\lambda_{lmwl}$ and $\Delta'^{17}O$ per Köppen-Geiger climatic zone. ($P_m$ = mean precipitation depth per month, MAT – mean annual temperature in °C; MARH – mean annual relative humidity [unweighted and weighted] in %). Selection of data points followed reproducibility criteria for inclusion in the GMWL, except where "all data" are stated.*

| Zone | Subset | n | Slope | Intercept | MAT °C | Mean $\delta^{18}O$ | Mean $\Delta'^{17}O$ | MARH / wt. MARH % |
|---|---|---|---|---|---|---|---|---|
| A | | 19 | 0.5269 | 0.0111 | +25.0 | -4.16 | 0.0152 | 78.2 / 81.5 |
| A | > 200 mm $P_m$ | 6 | 0.5273 | 0.0127 | +26.3 | -4.64 | 0.0153 | 85.5 / 87.0 |
| A | ≤ 200 mm $P_m$ | 13 | 0.5267 | 0.0104 | +24.4 | -3.94 | 0.0151 | 74.8 / 78.9 |
| B | | 5 | 0.5253 | 0.0131 | +27.4 | -2.39 | 0.0138 | 55.6 / 72.0 |
| C | | 33 | 0.5265 | 0.0087 | +14.7 | -6.76 | 0.0171 | 73.3 / 75.6 |
| C | > 100 mm $P_m$ | 8 | 0.5275 | 0.0124 | +16.8 | -7.61 | 0.0149 | 79.0 / 81.4 |
| C | ≤ 100 mm $P_m$ | 25 | 0.5262 | 0.0075 | +14.0 | -6.68 | 0.0179 | 71.5 / 73.7 |
| D | | 5 | 0.5269 | 0.0068 | +6.0 | -11.11 | 0.0176 | 77.0 / 77.7 |
| D | all data | 8 | 0.5272 | 0.0078 | +1.0 | -14.68 | 0.0149 | 75.0 / 75.9 |
| E | | 4 | 0.5283 | 0.0180 | -9.3 | -16.23 | 0.0100 | 73.4 / 73.4 |
| E | all data | 7 | 0.5288 | 0.0242 | -13.1 | -19.87 | 0.0050 | 75.1 / 75.5 |

*Table S8: Seasonal MWLs for selected stations*

| Clim. | Station | Period | Season | $\delta^{18}O$ Wt. mean | $\Delta'^{17}O$ Wt. mean | $\lambda_{mwl}$ | $\gamma_{mwl}$ | AT °C | RH % | PPT mm |
|---|---|---|---|---|---|---|---|---|---|---|
| A | Réunion | DJF | Rainy | -3.93 | 0.0056 | 0.5255 | -0.0043 | 23.8 | 80.6 | 167.0 |
| | | JJA | Dry | +1.17 | -0.0010 | 0.5203 | 0.0079 | 18.7 | 77.8 | 56.2 |
| B | Ascension | DJF | Dry | -0.12 | 0.0101 | 0.5210 | 0.0101 | 25.2 | 79.1 | 16.1 |
| | | MAM | Rainy | -1.07 | 0.0126 | 0.5258 | 0.0104 | 26.6 | 74.9 | 17.7 |
| B | Travellers Hill | DJF | Dry | -0.34 | 0.0102 | 0.5246 | 0.0096 | 25.1 | 79.0 | 32.2 |
| | | MAM | Rainy | -0.50 | 0.0116 | 0.5226 | 0.0084 | 26.6 | 75.2 | 46.1 |
| C | Hong Kong | DJF | Dry | -3.53 | 0.0146 | 0.5291 | 0.0184 | 17.6 | 75.5 | 42.8 |
| | | JJA | Rainy | -7.58 | 0.0118 | 0.5292 | 0.0206 | 28.5 | 85.0 | 409.2 |
| C | Isla de Pascua | DJF | Warmer | -2.33 | 0.0099 | 0.5244 | 0.0015 | 23.1 | 77.0 | 61.7 |
| | | JJA | Cooler | -2.84 | 0.0190 | 0.5252 | 0.0109 | 19.4 | 71.9 | 67.7 |
| C | Pta. Delgada | DJF | Winter | -4.05 | 0.0238 | 0.5283 | 0.0251 | 14.9 | 79.3 | 91.8 |
| | | JJA | Summer | -2.51 | 0.0167 | 0.5278 | 0.0161 | 20.7 | 76.8 | 35.5 |
| C | Reykjavik | DJF | Winter | -9.85 | 0.0215 | 0.5279 | 0.0210 | 0.0 | 81.7 | 89.8 |
| | | JJA | Summer | -8.40 | 0.0147 | 0.5266 | 0.0032 | 10.6 | 78.9 | 46.0 |
| C | Valentia | DJF | Winter | -5.59 | 0.0229 | 0.5261 | 0.0119 | 7.6 | 84.1 | 195.2 |
| | | JJA | Summer | -4.18 | 0.0106 | 0.5236 | -0.0083 | 14.4 | 83.7 | 106.2 |
| C | Vienna | DJF | Winter | -11.83 | 0.0224 | 0.5274 | 0.0148 | 2.1 | 77.5 | 43.6 |
| | | JJA | Summer | -5.44 | 0.0077 | 0.5242 | -0.0134 | 20.7 | 63.0 | 64.8 |
| E | Danmarks-havn | DJF | Winter | -17.01 | 0.0099 | 0.5295 | 0.0358 | -20.3 | 71.0 | 33.1 |
| | | JJA | Summer | -14.37 | 0.0096 | 0.5282 | 0.0120 | 1.1 | 86.3 | 17.9 |
| E | Halley Bay | JJA | Winter | -27.74 | -0.0004 | 0.5297 | 0.0475 | -23.4 | 56.8 | 38.5 |
| | | DJF | Summer | -16.81 | 0.0078 | 0.5287 | 0.0197 | -8.7 | 69.3 | 21.8 |

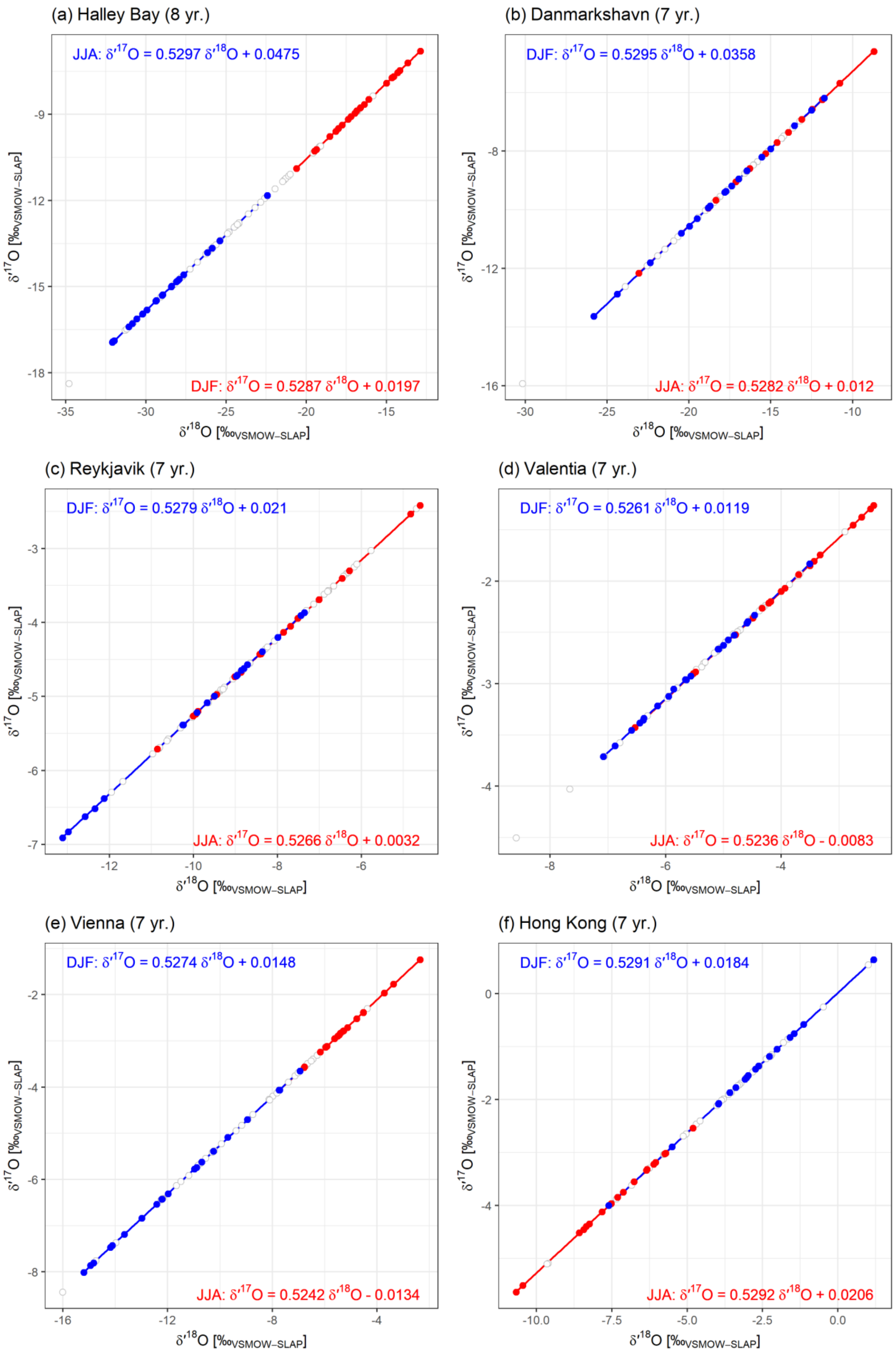
(a) Halley Bay (8 yr.)
JJA: δ'17O = 0.5297 δ'18O + 0.0475
DJF: δ'17O = 0.5287 δ'18O + 0.0197
(b) Danmarkshavn (7 yr.)
DJF: δ'17O = 0.5295 δ'18O + 0.0358
JJA: δ'17O = 0.5282 δ'18O + 0.012
(c) Reykjavik (7 yr.)
DJF: δ'17O = 0.5279 δ'18O + 0.021
JJA: δ'17O = 0.5266 δ'18O + 0.0032
(d) Valentia (7 yr.)
DJF: δ'17O = 0.5261 δ'18O + 0.0119
JJA: δ'17O = 0.5236 δ'18O - 0.0083
(e) Vienna (7 yr.)
DJF: δ'17O = 0.5274 δ'18O + 0.0148
JJA: δ'17O = 0.5242 δ'18O - 0.0134
(f) Hong Kong (7 yr.)
DJF: δ'17O = 0.5291 δ'18O + 0.0184
JJA: δ'17O = 0.5292 δ'18O + 0.0206
δ'18O [‰VSMOW–SLAP]
δ'17O [‰VSMOW–SLAP]

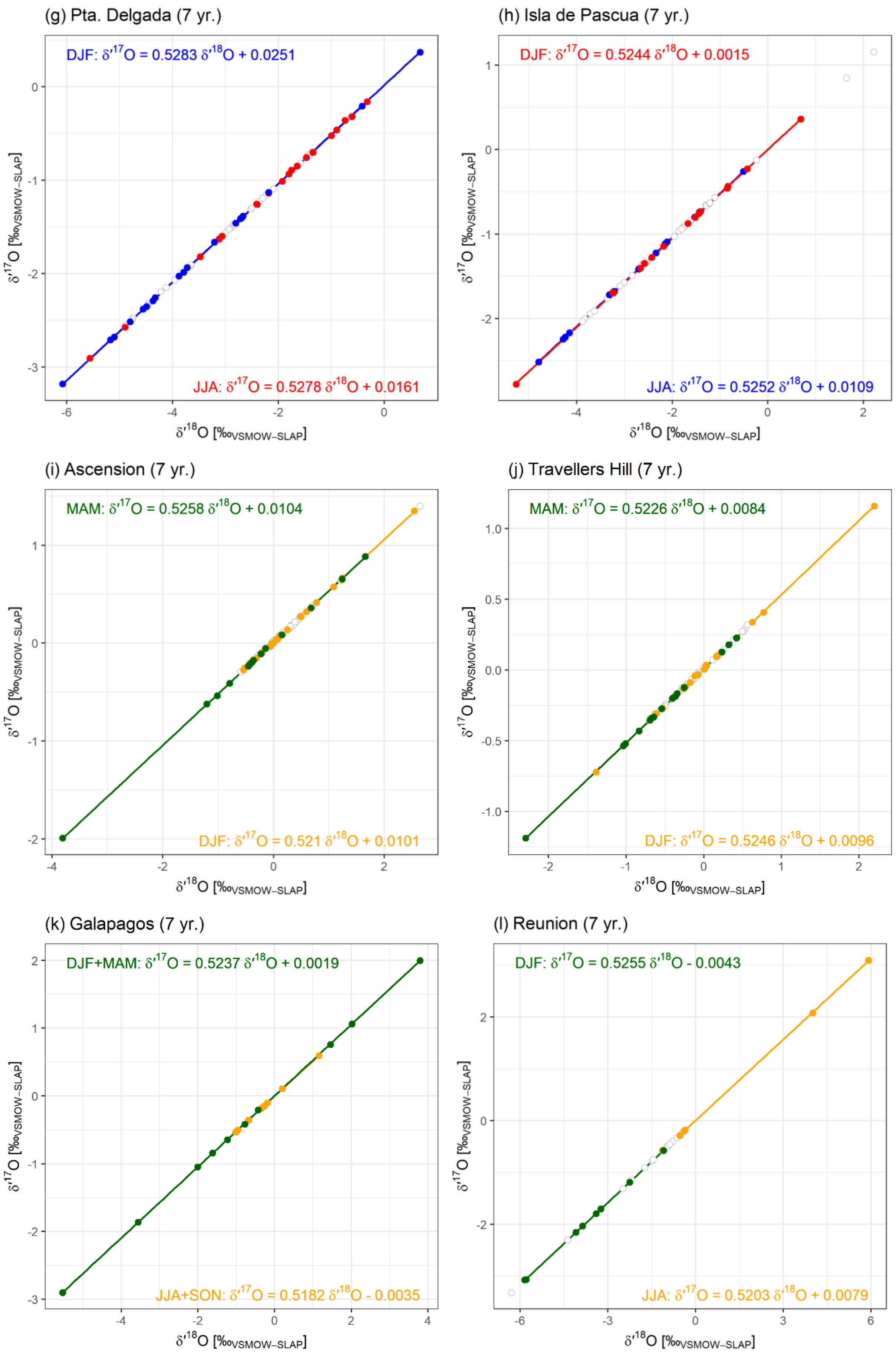


*Figure S3: Seasonal MWLs for 12 sites.*

## S7 Correlations of $\lambda_{lmwl}$ and $\Delta'^{17}O$ with meteorological parameters

*Table S9: Correlations of $\lambda_{lmwl}$ with meteorological parameters for wet tropical (Köppen type A) and other climate types. Only those sites were included in the correlation analysis which also were chosen for the GMWL calculation.*

| ERA5 parameter | Abbre-viation | Wet Tropical (A) | | Other (B, C, D, E) | |
|---|---|---|---|---|---|
| | | $R^2$ | Correlation | $R^2$ | Correlation |
| Wind speed at 10m height | si10 | 0.38 | Negative | 0.08 | Negative |
| Total precipitation | tp | 0.31 | Positive | 0.10 | Positive |
| Mean total precipitation rate | mtpr | 0.31 | Positive | 0.10 | Positive |
| Convective precipitation amount | cp | 0.30 | Positive | 0.02 | Positive |
| Mean convective precipitation rate | mcpr | 0.30 | Positive | 0.02 | Positive |
| Convective inhibition | cin | 0.29 | Negative | 0.06 | Negative |
| Total column liquid water content | tclw | 0.23 | Positive | 0.03 | Positive |
| Convective rain rate | crr | 0.22 | Positive | 0.02 | Positive |
| Cloud base height | cbh | 0.21 | Negative | 0.01 | Positive |
| Large-scale precipitation rate | lsrr | 0.18 | Positive | 0.09 | Positive |
| Large-scale precipitation amount | lsp | 0.19 | Positive | 0.16 | Positive |
| Air temperature at 2m height | t2m | 0.16 | Negative | 0.11 | Negative |
| Surface-level pressure | sp | 0.14 | Negative | 0.03 | Negative |
| Total column rainwater | tcrw | 0.10 | Positive | 0.12 | Positive |

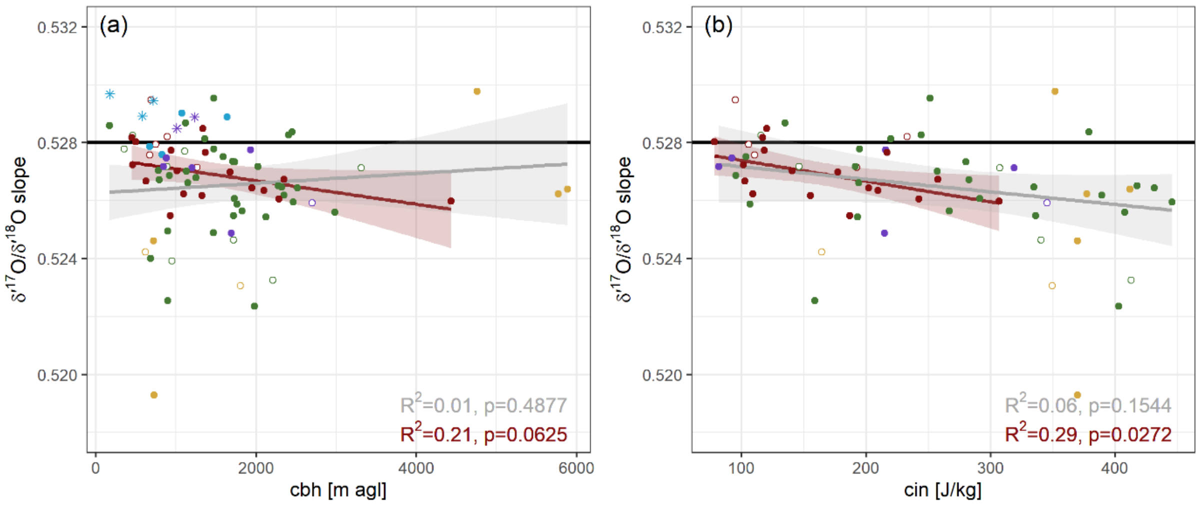

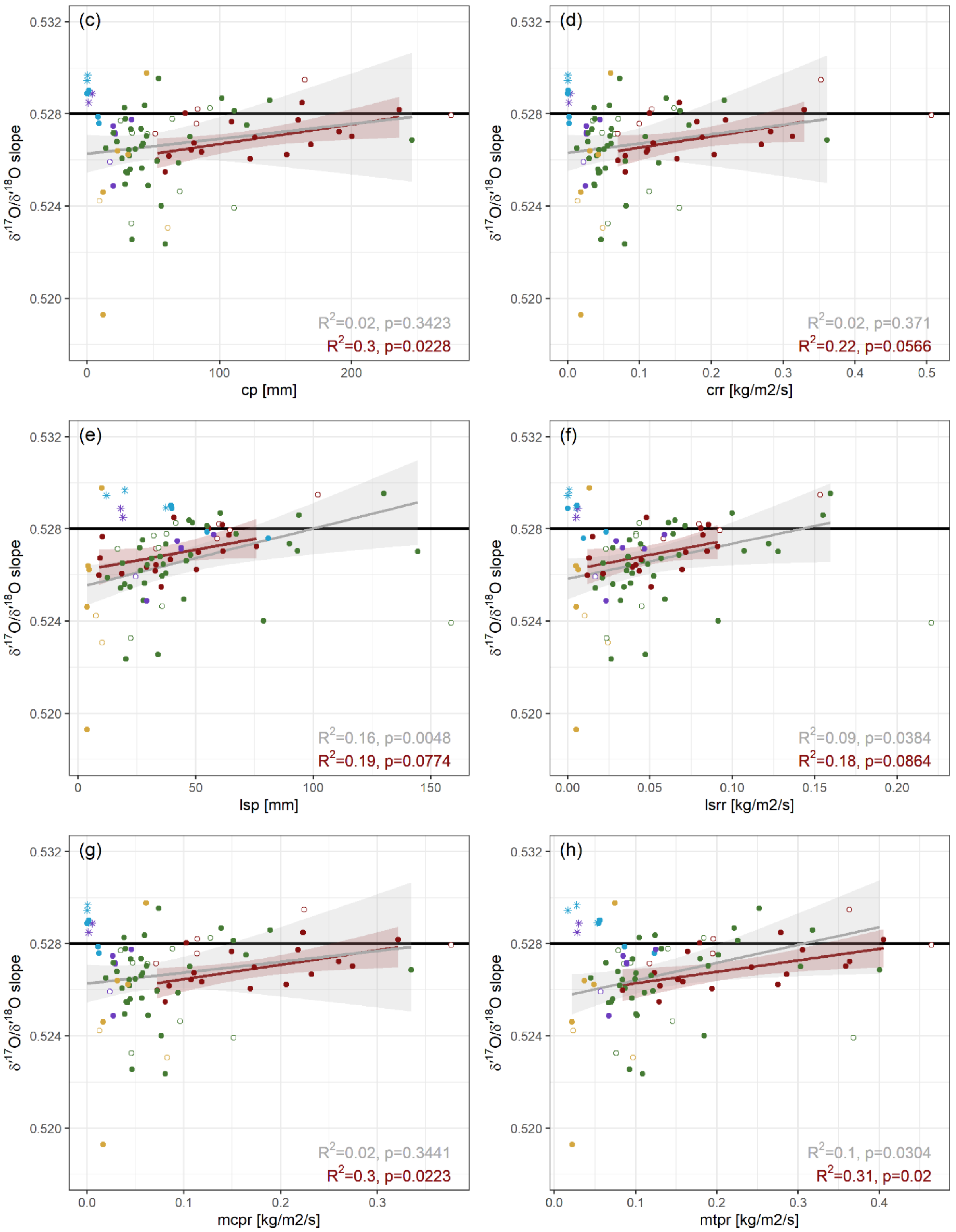

(c)
0.532
0.528
0.524
0.520
δ'17O/δ'18O slope
0
100
200
cp [mm]
R2=0.02, p=0.3423
R2=0.3, p=0.0228
(d)
0.0
0.1
0.2
0.3
0.4
0.5
crr [kg/m2/s]
R2=0.02, p=0.371
R2=0.22, p=0.0566
(e)
0
50
100
150
lsp [mm]
R2=0.16, p=0.0048
R2=0.19, p=0.0774
(f)
0.00
0.05
0.10
0.15
0.20
lsrr [kg/m2/s]
R2=0.09, p=0.0384
R2=0.18, p=0.0864
(g)
0.0
0.1
0.2
0.3
mcpr [kg/m2/s]
R2=0.02, p=0.3441
R2=0.3, p=0.0223
(h)
0.0
0.1
0.2
0.3
0.4
mtpr [kg/m2/s]
R2=0.1, p=0.0304
R2=0.31, p=0.02

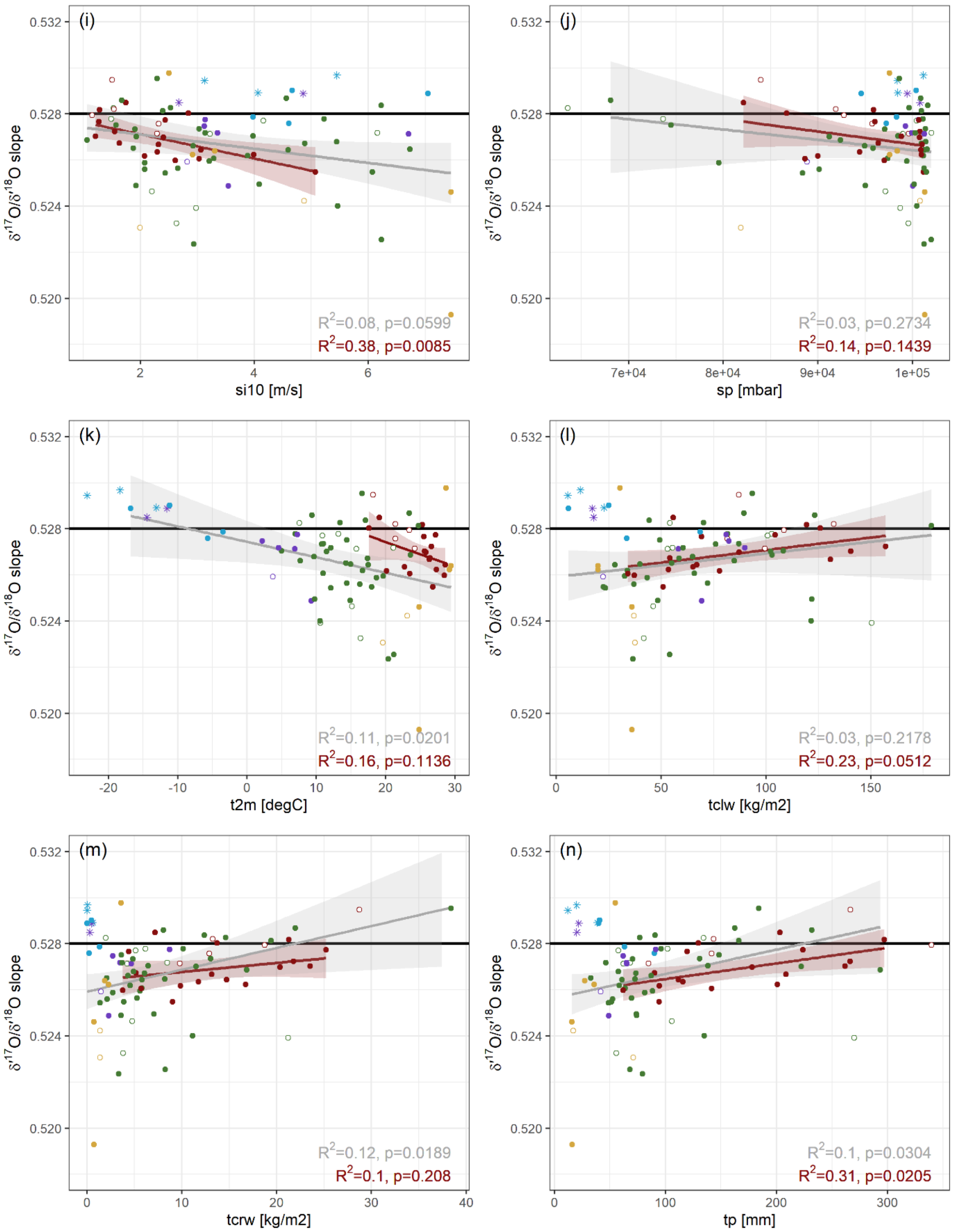


*Figure S4: Relationship of the $\delta'^{17}O/\delta'^{18}O$ slope with meteorological parameters. Red line is the regression of A climates with the 95% confidence interval shaded, the grey line for extratropical sites. Full circles are stations included in the GMWL calculation and correlation testing; empty circles were omitted. Asterisks denote stations in the Canadian Arctic.*

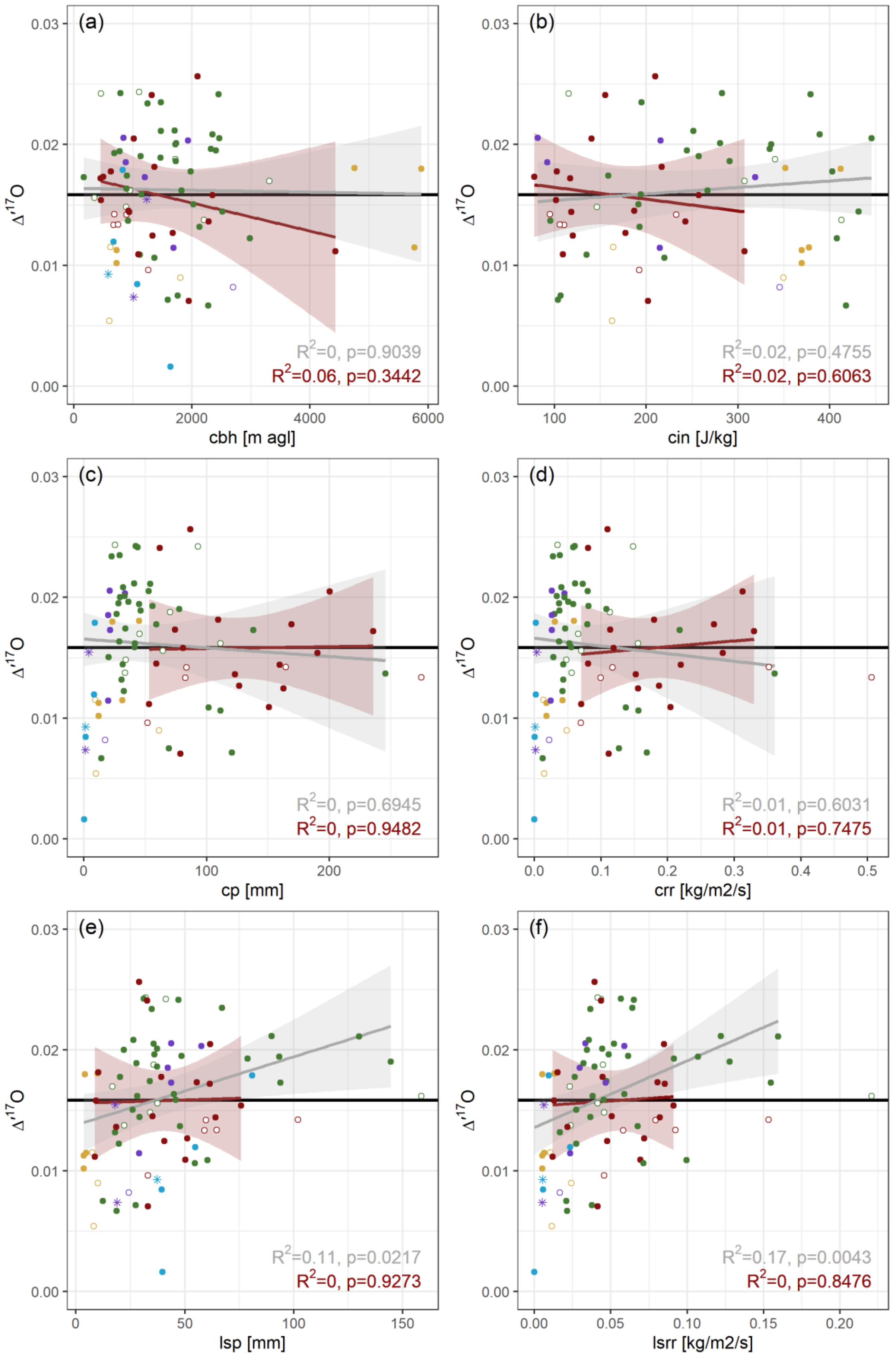
(a)
R^2=0, p=0.9039
R^2=0.06, p=0.3442
cbh [m agl]
(b)
R^2=0.02, p=0.4755
R^2=0.02, p=0.6063
cin [J/kg]
(c)
R^2=0, p=0.6945
R^2=0, p=0.9482
cp [mm]
(d)
R^2=0.01, p=0.6031
R^2=0.01, p=0.7475
crr [kg/m2/s]
(e)
R^2=0.11, p=0.0217
R^2=0, p=0.9273
lsp [mm]
(f)
R^2=0.17, p=0.0043
R^2=0, p=0.8476
lsrr [kg/m2/s]
Δ'17O

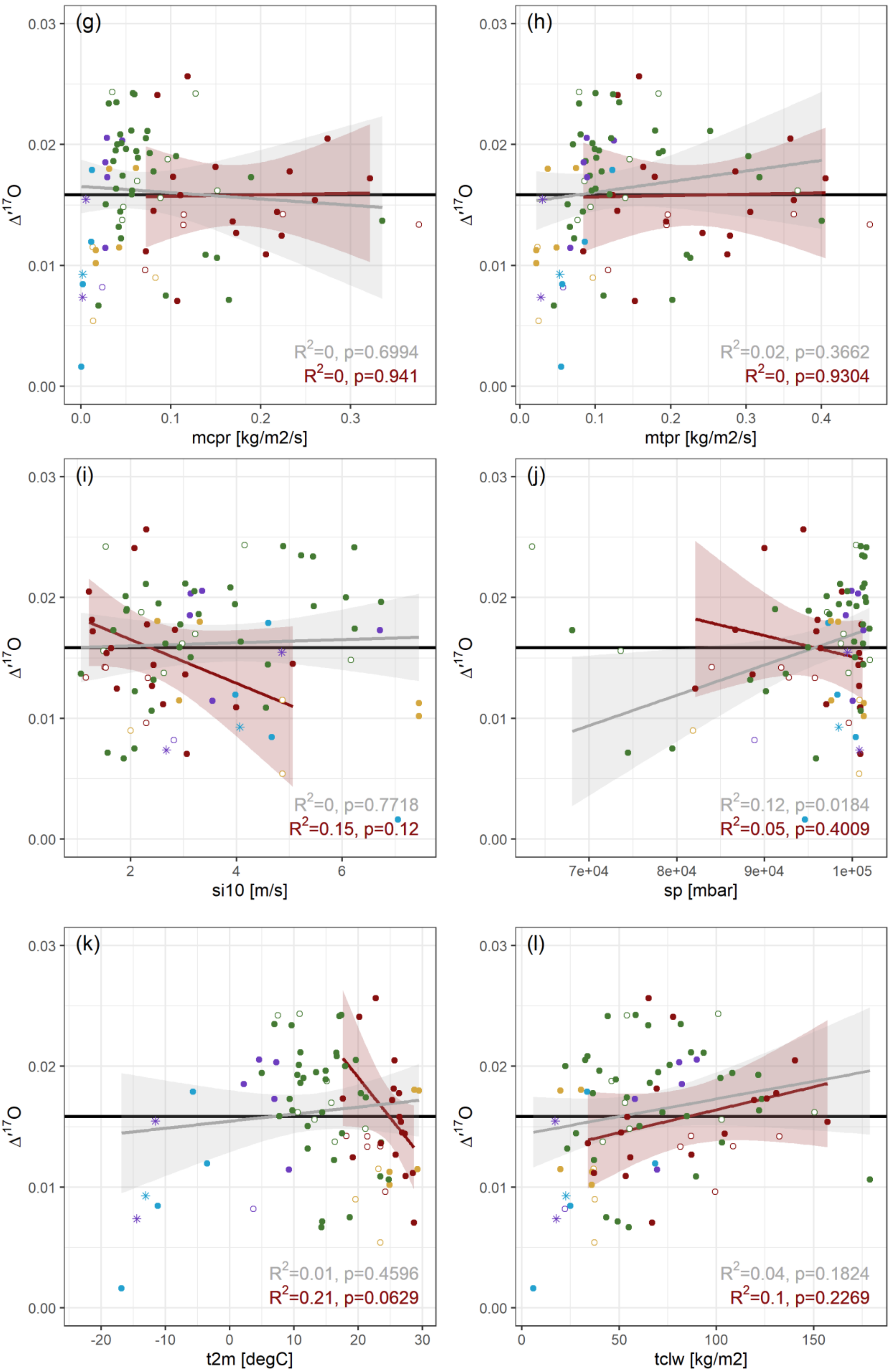
(g)
R²=0, p=0.6994
R²=0, p=0.941
mcpr [kg/m2/s]
(h)
R²=0.02, p=0.3662
R²=0, p=0.9304
mtpr [kg/m2/s]
(i)
R²=0, p=0.7718
R²=0.15, p=0.12
si10 [m/s]
(j)
R²=0.12, p=0.0184
R²=0.05, p=0.4009
sp [mbar]
(k)
R²=0.01, p=0.4596
R²=0.21, p=0.0629
t2m [degC]
(l)
R²=0.04, p=0.1824
R²=0.1, p=0.2269
tclw [kg/m2]
Δ'17O

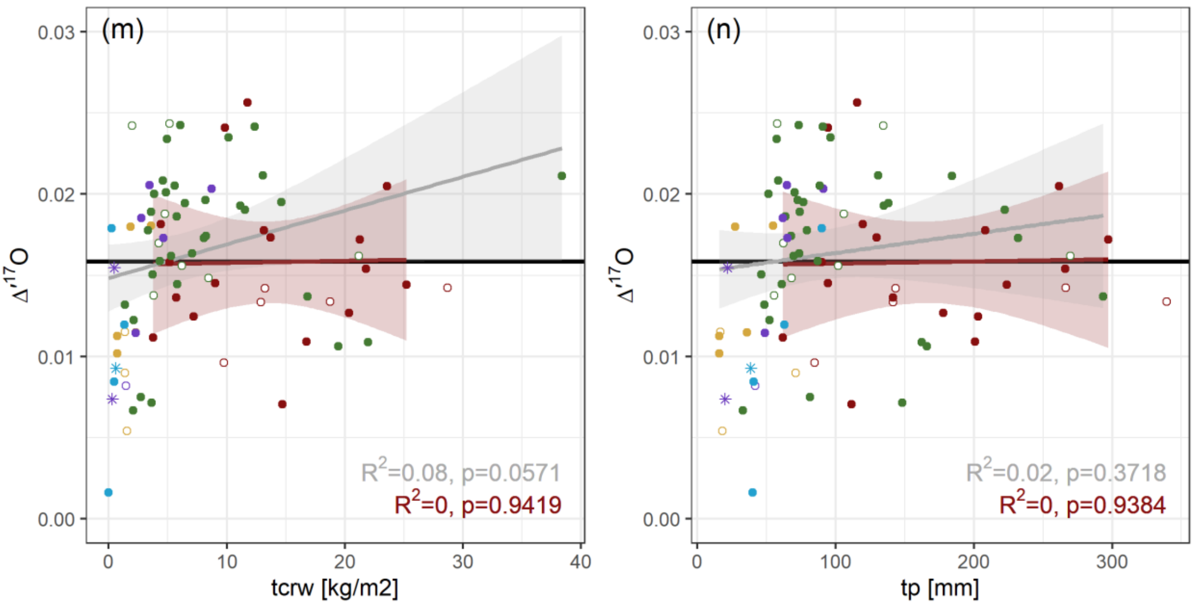


*Figure S5: Relationship of Δ'17O with meteorological parameters. Red line is the regression of A climates with the 95% confidence interval shaded, the grey line for extratropical sites. Full circles are stations included in the GMWL calculation and correlation testing; empty circles were omitted. Asterisks denote stations in the Canadian Arctic.*

# S8 Seasonal/regional patterns

## S8.1 Mediterranean Basin

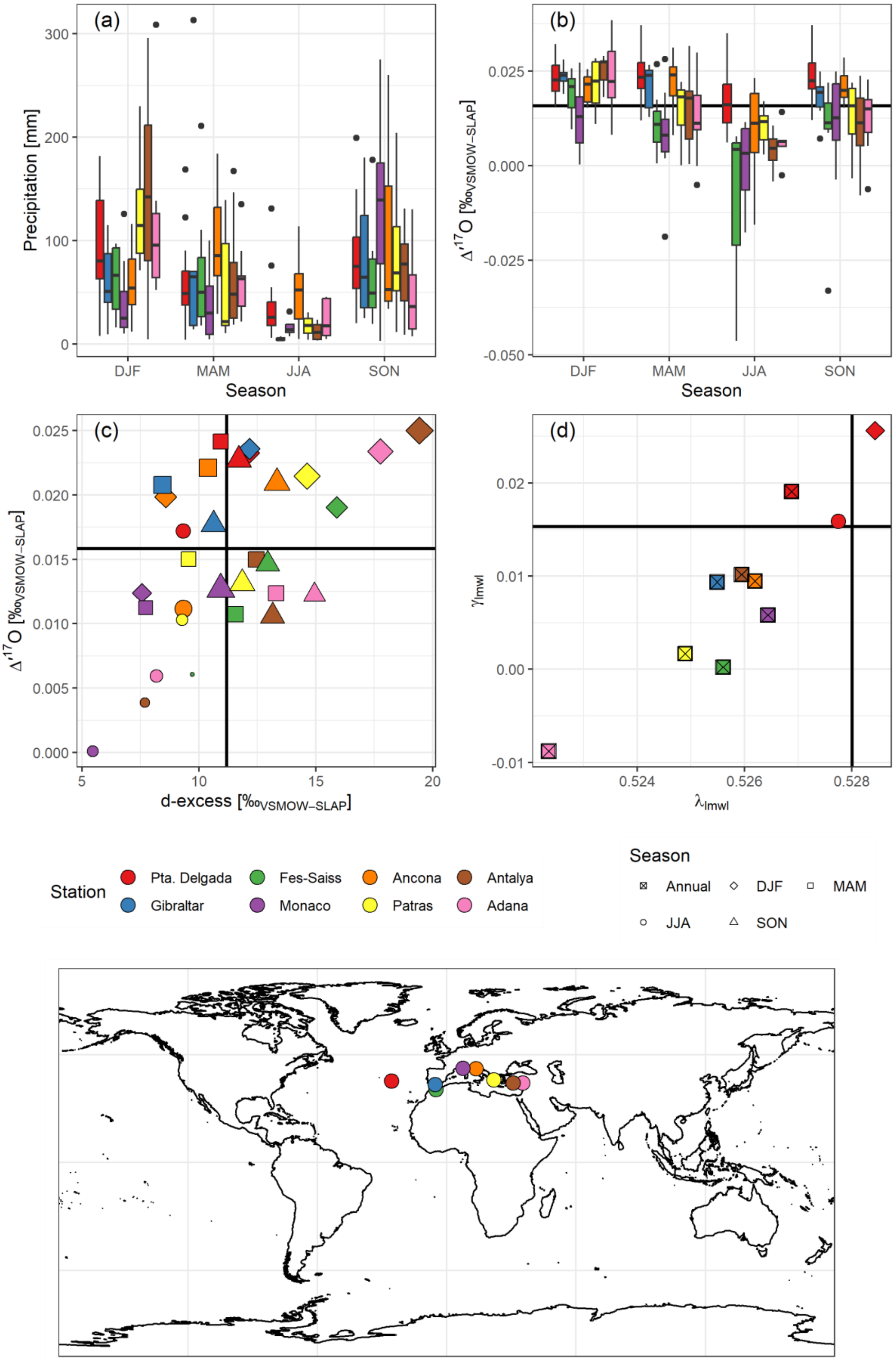


*Figure S6: Mediterranean basin. Seasonal distribution of precipitation depth (a), $\Delta'^{17}O$ (b), $\Delta'^{17}O$ vs. d-excess (c), $\lambda_{lmwl}$ vs. $\gamma_{lmwl}$ (d). Thick black lines indicate global mean $\Delta'^{17}O$ (b), $\Delta'^{17}O$ and d-excess (c), $\lambda_{gmwl}$ and $\gamma_{gmwl}$ (d).*

*Table S10: Correlation matrix of $\lambda_{mwl}$, $\gamma_{mwl}$ and $\Delta'^{17}O$ against spatial, isotopic and climatologic variables for the Mediterranean Basin. Spherical distance is measured in km from Ponta Delgada and overlaps largely with longitude. For explanations, please see main text. Asterisks (*, ** or ***) indicate significance of correlation at the 0.01, 0.001 and 0.0001 levels respectively.*

| Independent variable | Dependent variables | | | | | | |
|---|---|---|---|---|---|---|---|
| | $\lambda_{mwl}$ | $\gamma_{mwl}$ | $\Delta'^{17}O$ mean | $\Delta'^{17}O$ DJF | $\Delta'^{17}O$ MAM | $\Delta'^{17}O$ JJA | $\Delta'^{17}O$ SON |
| Slope | | **0.84**** | | | | | |
| Latitude | 0.03 | 0.01 | 0.01 | 0.19 | 0.04 | 0.24 | 0.33 |
| Longitude | **0.62*** | **0.59*** | 0.01 | 0.11 | 0.13 | 0.09 | 0.14 |
| Distance | **0.62*** | **0.60*** | 0.01 | 0.11 | 0.14 | 0.11 | 0.16 |
| $\delta'^{18}O$ | 0.18 | 0.28 | 0.10 | 0.01 | 0.04 | 0.43 | 0.04 |
| d-excess | 0.33 | 0.15 | 0.06 | 0.44 | 0.01 | **0.84*** | 0.08 |
| Precipitation | 0.00 | 0.05 | 0.58 | 0.59 | 0.25 | 0.64 | 0.25 |
| Air temp. (ERA5) | 0.01 | 0.00 | 0.25 | 0.04 | 0.07 | 0.06 | 0.01 |
| RH (ERA5) | 0.24 | 0.46 | 0.44 | 0.11 | 0.25 | 0.29 | 0.69 |

*Table S11: Seasonal median values of precipitation depth and median/IQR values of $\delta^{18}O$*

| Station | Median precipitation [mm mo$^{-1}$] | | | | Median $\delta^{18}O$ | | | | IQR $\delta^{18}O$ | | | |
|---|---|---|---|---|---|---|---|---|---|---|---|---|
| | DJF | MAM | JJA | SON | DJF | MAM | JJA | SON | DJF | MAM | JJA | SON |
| Pta. Delgada | 80.3 | 48.8 | 25.6 | 74.9 | -3.83 | -2.91 | -1.78 | -2.80 | 1.72 | 0.69 | 1.30 | 1.45 |
| Gibraltar | 50.5 | 65.0 | --- | 64.4 | -5.10 | -2.94 | --- | -4.05 | 1.73 | 0.28 | --- | 1.50 |
| Fes-Saïss | 66.3 | 50.0 | 4.2 | 49.0 | -4.06 | -3.44 | -1.50 | -4.35 | 3.51 | 3.32 | 6.82 | 3.01 |
| Monaco | 24.8 | 29.8 | 13.8 | 139 | -4.67 | -4.09 | -3.39 | -5.54 | 2.09 | 2.74 | 1.32 | 0.68 |
| Ancona | 54.1 | 85.4 | 52.0 | 52.5 | -4.89 | -5.38 | -3.92 | -6.41 | 2.17 | 3.16 | 1.80 | 1.94 |
| Patras | 114.6 | 21.7 | 18.0 | 68.6 | -6.15 | -5.12 | -3.58 | -5.28 | 2.48 | 2.21 | 0.94 | 3.08 |
| Antalya | 142.1 | 48.1 | 11.0 | 77.3 | -5.32 | -3.46 | -3.03 | -3.40 | 1.76 | 2.89 | 2.75 | 1.94 |
| Adana | 95.3 | 62.9 | 17.3 | 36 | -5.73 | -3.98 | -3.34 | -3.91 | 0.76 | 2.20 | 0.90 | 1.41 |

*Table S812: Seasonal median values of d-excess and median/IQR values $\Delta'^{17}O$*

| Station | Median *d*-excess | | | | Median $\Delta'^{17}O$ | | | | IQR $\Delta'^{17}O$ | | | |
|---|---|---|---|---|---|---|---|---|---|---|---|---|
| | DJF | MAM | JJA | SON | DJF | MAM | JJA | SON | DJF | MAM | JJA | SON |
| Pta. Delgada | 11.9 | 11.1 | 9.5 | 11.5 | 0.023 | 0.023 | 0.016 | 0.022 | 0.007 | 0.007 | 0.010 | 0.007 |
| Gibraltar | 12.3 | 9.0 | --- | 9.6 | 0.024 | 0.024 | --- | 0.019 | 0.002 | 0.010 | --- | 0.005 |
| Fes-Saïss | 16.6 | 10.8 | 7.4 | 11.8 | 0.021 | 0.011 | 0.004 | 0.011 | 0.008 | 0.008 | 0.027 | 0.007 |
| Monaco | 6.7 | 6.9 | 4.9 | 11.4 | 0.013 | 0.008 | 0.003 | 0.013 | 0.012 | 0.008 | 0.016 | 0.015 |
| Ancona | 6.6 | 10.6 | 9.4 | 14.1 | 0.022 | 0.024 | 0.011 | 0.020 | 0.007 | 0.008 | 0.016 | 0.006 |
| Patras | 15.6 | 10.4 | 9.3 | 11.7 | 0.022 | 0.018 | 0.012 | 0.016 | 0.011 | 0.009 | 0.006 | 0.012 |
| Antalya | 20.4 | 13.3 | 8.0 | 11.6 | 0.027 | 0.018 | 0.005 | 0.011 | 0.005 | 0.013 | 0.006 | 0.013 |
| Adana | 18.2 | 13.8 | 8.3 | 15.4 | 0.022 | 0.011 | 0.006 | 0.015 | 0.012 | 0.009 | 0.002 | 0.009 |

Comments see main manuscript text.

## S8.2 Southeast and East Asia

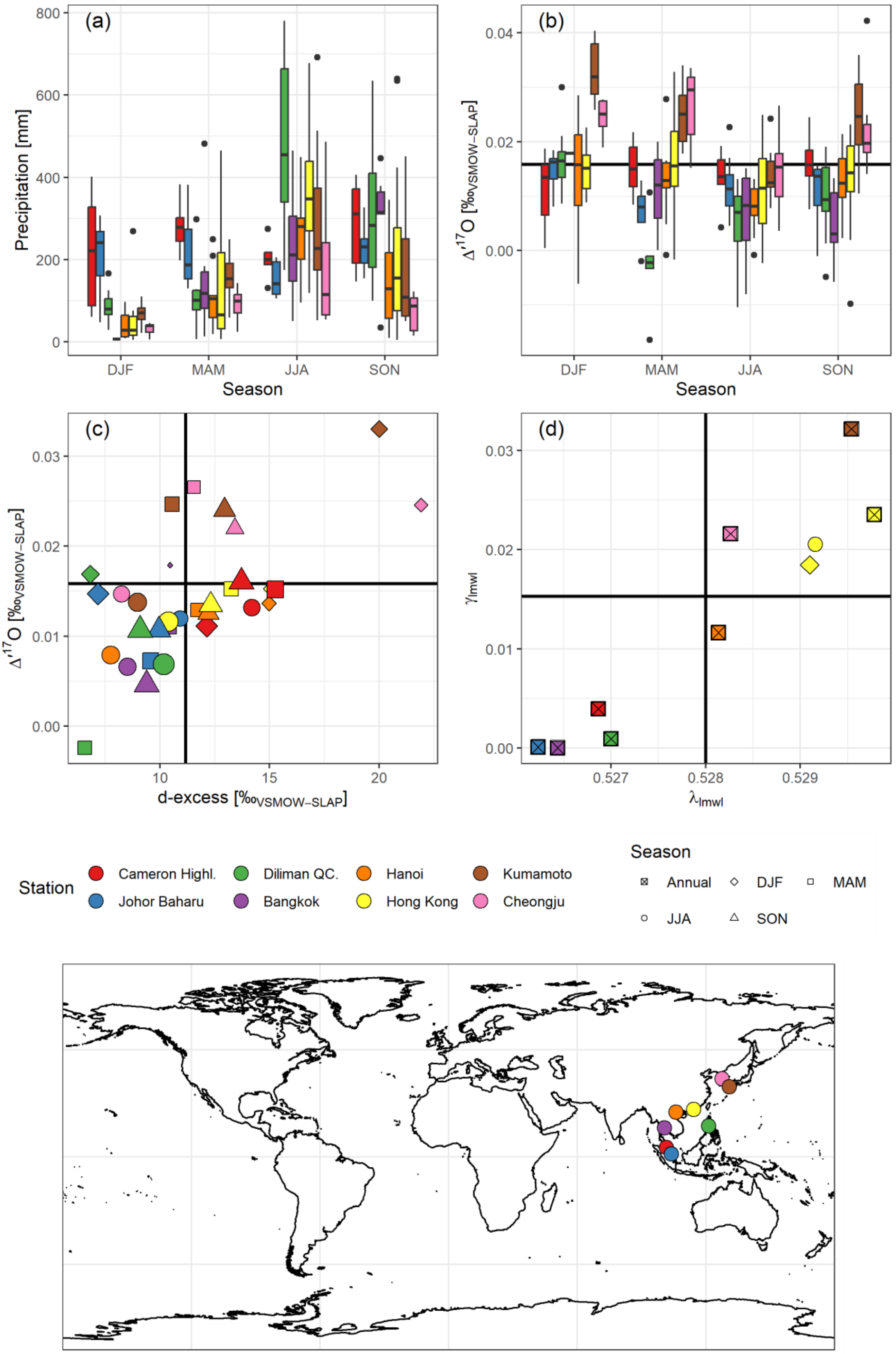


*Figure S7: (South)East Asia. Seasonal distribution of precipitation depth (a), $\Delta'^{17}O$ (b), $\Delta'^{17}O$ vs. d-excess (c), $\lambda_{lmwl}$ vs. $\gamma_{lmwl}$ (d). Thick black lines indicate global mean $\Delta'^{17}O$ (b), $\Delta'^{17}O$ and d-excess (c), $\lambda_{gmwl}$ and $\gamma_{gmwl}$ (d).*

*Table S13: Correlation matrix of $\lambda_{mwl}$, $\gamma_{mwl}$ and $\Delta'^{17}O$ against spatial, isotopic and climatologic variables for the (South-)East Asia region. Spherical distance is measured in km from Johor Bahru. For explanations, please see main text. Asterisks (*, ** or ***) indicate significance of correlation at the 0.01, 0.001 and 0.0001 levels respectively.*

| Independent variable | Dependent variables | | | | | | |
|---|---|---|---|---|---|---|---|
| | $\lambda_{mwl}$ | $\gamma_{mwl}$ | $\Delta'^{17}O$ mean | $\Delta'^{17}O$ DJF | $\Delta'^{17}O$ MAM | $\Delta'^{17}O$ JJA | $\Delta'^{17}O$ SON |
| Slope | | **0.88*** ** | | | | | |
| Latitude | 0.56 | **0.71*** | 0.44 | 0.6 | 0.53 | 0.13 | 0.41 |
| Longitude | 0.35 | 0.51 | 0.42 | 0.6 | 0.2 | 0.16 | 0.44 |
| Distance | 0.49 | **0.65*** | 0.44 | 0.66 | 0.42 | 0.12 | 0.42 |
| $\delta'^{18}O$ | 0.00 | 0.04 | 0.45 | 0.01 | 0.26 | 0.26 | 0.56 |
| d-excess | 0.17 | 0.16 | 0.38 | 0.46 | 0.34 | 0.18 | 0.43 |
| Precipitation | 0.05 | 0.08 | 0.39 | 0.21 | 0.04 | 0.25 | 0.55 |
| Air temp. (ERA5) | 0.26 | 0.45 | **0.91**** | 0.53 | **0.76*** | 0.45 | **0.90**** |
| RH (ERA5) | 0.05 | 0.17 | 0.33 | 0.51 | 0.33 | 0.02 | 0.30 |

*Table S14: Seasonal median values of precipitation depth and median/IQR values of $\delta^{18}O$*

| Station | Median precipitation [mm $mo^{-1}$] | | | | Median $\delta^{18}O$ | | | | IQR $\delta^{18}O$ | | | |
|---|---|---|---|---|---|---|---|---|---|---|---|---|
| | DJF | MAM | JJA | SON | DJF | MAM | JJA | SON | DJF | MAM | JJA | SON |
| Cameron H. | 221.2 | 278.7 | 200.0 | 311.3 | -7.35 | -7.70 | -8.29 | -9.65 | 2.75 | 2.43 | 2.64 | 1.02 |
| Johor Baharu | 241.2 | 187.2 | 140.9 | 230.4 | -6.78 | -5.29 | -5.72 | -6.64 | 2.18 | 2.13 | 1.07 | 1.36 |
| Diliman QC. | 79.7 | 100.9 | 454.4 | 283.0 | -4.33 | -1.41 | -5.87 | -6.47 | 2.07 | 1.25 | 1.58 | 3.15 |
| Bangkok | 6.8 | 117.4 | 211.4 | 315.0 | -1.09 | -4.91 | -4.24 | -4.54 | 0.00 | 3.96 | 1.41 | 3.31 |
| Hanoi | 28.6 | 104.5 | 280.4 | 128.8 | -2.98 | -2.42 | -8.32 | -7.68 | 2.04 | 3.26 | 2.91 | 2.95 |
| Hong Kong | 28.5 | 65.9 | 347.4 | 155.4 | -3.00 | -3.13 | -6.92 | -5.44 | 1.47 | 1.31 | 1.82 | 2.47 |
| Kumamoto | 69.9 | 152.8 | 227.1 | 108.3 | -6.11 | -5.57 | -8.01 | -6.74 | 2.35 | 1.58 | 1.76 | 2.43 |
| Cheongju | 39.3 | 99.5 | 115.3 | 87.0 | -7.45 | -4.98 | -7.82 | -8.59 | 3.09 | 1.17 | 3.3 | 2.25 |

*Table S15: Seasonal median values of d-excess and median/IQR values $\Delta'^{17}O$*

| Station | Median *d*-excess | | | | Median $\Delta'^{17}O$ | | | | IQR $\Delta'^{17}O$ | | | |
|---|---|---|---|---|---|---|---|---|---|---|---|---|
| | DJF | MAM | JJA | SON | DJF | MAM | JJA | SON | DJF | MAM | JJA | SON |
| Cameron H. | 13.1 | 15.3 | 14.4 | 13.7 | 0.013 | 0.015 | 0.014 | 0.016 | 0.009 | 0.007 | 0.005 | 0.005 |
| Johor Baharu | 8.2 | 8.6 | 11.0 | 10.0 | 0.016 | 0.008 | 0.011 | 0.014 | 0.004 | 0.005 | 0.006 | 0.007 |
| Diliman QC. | 7.4 | 7.0 | 9.9 | 9.5 | 0.016 | -0.002 | 0.007 | 0.009 | 0.005 | 0.002 | 0.008 | 0.008 |
| Bangkok | 10.5 | 10.1 | 7.8 | 11.1 | 0.018 | 0.012 | 0.008 | 0.003 | 0.000 | 0.011 | 0.011 | 0.009 |
| Hanoi | 16.8 | 12.8 | 8.8 | 12.5 | 0.016 | 0.013 | 0.008 | 0.012 | 0.013 | 0.005 | 0.005 | 0.007 |
| Hong Kong | 15.2 | 13.1 | 10.3 | 12.6 | 0.015 | 0.016 | 0.011 | 0.014 | 0.006 | 0.010 | 0.012 | 0.008 |
| Kumamoto | 19.7 | 10.3 | 8.9 | 10.6 | 0.032 | 0.025 | 0.012 | 0.025 | 0.009 | 0.008 | 0.005 | 0.011 |
| Cheongju | 23.3 | 11.3 | 8.7 | 11.3 | 0.025 | 0.030 | 0.015 | 0.020 | 0.005 | 0.011 | 0.008 | 0.005 |

Comments see main manuscript text.

## S8.3 Oceanic islands

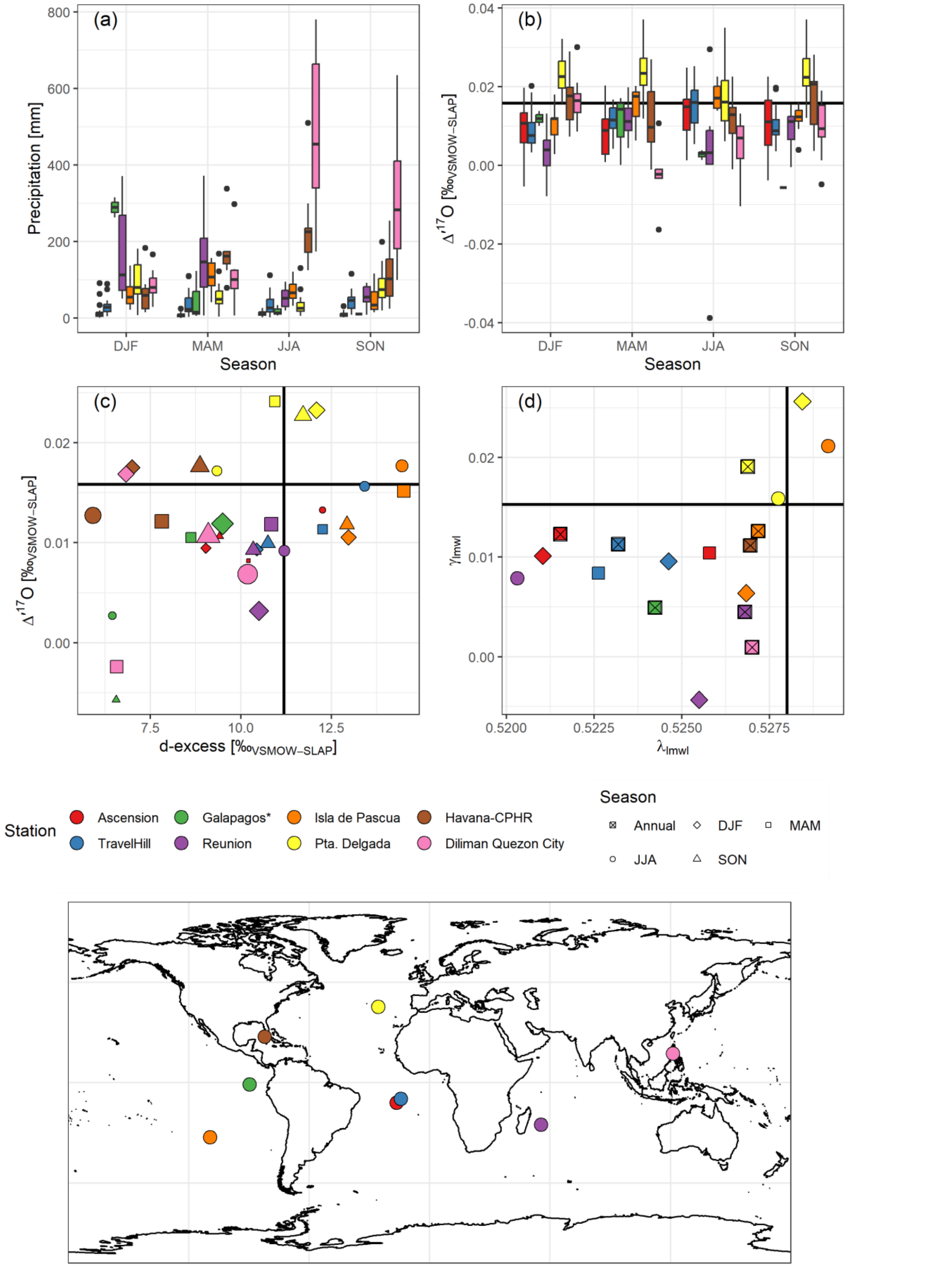


*Figure S8: Oceanic Islands. Seasonal distribution of precipitation depth (a), $\Delta'^{17}O$ (b), $\Delta'^{17}O$ vs. d-excess (c), $\lambda_{lmwl}$ vs. $\gamma_{lmwl}$ (d). Thick black lines indicate global mean $\Delta'^{17}O$ (b), $\Delta'^{17}O$ and d-excess (c), $\lambda_{gmwl}$ and $\gamma_{gmwl}$ (d). Stations marked with an asterisk failed to cover >50% of the precipitation with reproducible isotopic information and were discarded from the global analysis.*

*Table S16: Correlation matrix of $\lambda_{mwl}$, $\gamma_{mwl}$ and $\Delta'^{17}O$ against spatial, isotopic and climatologic variables for the small islands domain. Longitude and distance were not evaluated. Caution is advised in the interpretation as the timing of seasons varies (northern and southern hemispheres). For explanations, please see main text. Asterisks (*, ** or ***) indicate significance of correlation at the 0.01, 0.001 and 0.0001 levels respectively.*

| Independent variable | Dependent variables | | | | | | |
|---|---|---|---|---|---|---|---|
| | $\lambda_{mwl}$ | $\gamma_{mwl}$ | $\Delta'^{17}O$ mean | $\Delta'^{17}O$ DJF | $\Delta'^{17}O$ MAM | $\Delta'^{17}O$ JJA | $\Delta'^{17}O$ SON |
| Slope | | 0.07 | | | | | |
| Latitude | 0.27 | **0.39*** | 0.14 | **0.72*** | 0.03 | 0.00 | 0.13 |
| Longitude | | | | | | | |
| Distance | | | | | | | |
| $\delta'^{18}O$ | **0.53**** | 0.01 | 0.00 | 0.11 | 0.01 | 0.00 | 0.03 |
| d-excess | 0.03 | 0.07 | 0.30 | 0.01 | 0.24 | 0.5 | 0.37 |
| Precipitation | 0.23 | 0.18 | 0.16 | 0.16 | 0.06 | 0.08 | 0.00 |
| Air temp. (ERA5) | 0.08 | 0.23 | 0.46 | 0.56 | 0.65 | 0.01 | 0.05 |
| RH (ERA5) | 0.00 | 0.14 | 0.39 | 0.13 | 0.00 | **0.71*** | 0.59 |

*Table S17: Seasonal median values of precipitation depth and median/IQR values of $\delta^{18}O$*

| Station | Median precipitation [mm $mo^{-1}$] | | | | Median $\delta^{18}O$ | | | | IQR $\delta^{18}O$ | | | |
|---|---|---|---|---|---|---|---|---|---|---|---|---|
| | DJF | MAM | JJA | SON | DJF | MAM | JJA | SON | DJF | MAM | JJA | SON |
| Ascension | 11.0 | 9.1 | 12.9 | 8.8 | +0.06 | -0.30 | +0.05 | +0.29 | 0.74 | 0.72 | 0.41 | 0.42 |
| Travellers Hill | 28.3 | 23.3 | 26.2 | 46.2 | -0.12 | -0.40 | -0.27 | -0.08 | 0.34 | 0.67 | 0.29 | 0.29 |
| Galapagos* | 289.2 | 15.1 | 13.6 | 11.0 | -1.62 | -1.62 | -0.19 | -0.27 | 0.38 | 2.78 | 0.61 | 0.00 |
| Reunion | 113.0 | 146.8 | 51.8 | 54.6 | -3.63 | -1.97 | -0.48 | -0.91 | 1.53 | 1.01 | 0.59 | 0.52 |
| Isla de Pascua | 55.1 | 107.4 | 65.8 | 33.7 | -2.05 | -2.66 | -2.52 | -1.71 | 0.98 | 0.80 | 1.04 | 1.12 |
| Pta. Delgada | 80.3 | 48.8 | 25.6 | 74.9 | -3.83 | -2.91 | -1.78 | -2.80 | 1.72 | 0.69 | 1.30 | 1.45 |
| Havana-CPHR | 59.0 | 161.7 | 225.4 | 102.5 | +0.40 | -2.45 | -1.99 | -1.43 | 2.35 | 2.38 | 2.93 | 2.35 |
| Diliman QC. | 79.7 | 100.9 | 454.4 | 283.0 | -4.33 | -1.41 | -5.87 | -6.47 | 2.07 | 1.25 | 1.58 | 3.15 |

*Table S18: Seasonal median values of d-excess and median/IQR values $\Delta'^{17}O$*

| Station | Median *d*-excess | | | | Median $\Delta'^{17}O$ | | | | IQR $\Delta'^{17}O$ | | | |
|---|---|---|---|---|---|---|---|---|---|---|---|---|
| | DJF | MAM | JJA | SON | DJF | MAM | JJA | SON | DJF | MAM | JJA | SON |
| Ascension | 10.3 | 10.6 | 12.1 | 9.1 | 0.011 | 0.009 | 0.015 | 0.011 | 0.008 | 0.009 | 0.008 | 0.012 |
| Travellers Hill | 10.4 | 12.0 | 13.6 | 10.8 | 0.008 | 0.011 | 0.016 | 0.009 | 0.005 | 0.005 | 0.008 | 0.004 |
| Galapagos* | 9.5 | 11.4 | 7.5 | 6.6 | 0.012 | 0.014 | 0.003 | -0.006 | 0.002 | 0.008 | 0.001 | 0.000 |
| Reunion | 10.4 | 11.8 | 11.6 | 10.7 | 0.004 | 0.011 | 0.003 | 0.011 | 0.006 | 0.006 | 0.009 | 0.006 |
| Isla de Pascua | 13.1 | 14.3 | 14.9 | 13.5 | 0.012 | 0.018 | 0.017 | 0.012 | 0.004 | 0.006 | 0.006 | 0.003 |
| Pta. Delgada | 11.9 | 11.1 | 9.5 | 11.5 | 0.023 | 0.023 | 0.016 | 0.022 | 0.007 | 0.007 | 0.01 | 0.007 |
| Havana-CPHR | 4.4 | 8.5 | 6.0 | 6.6 | 0.018 | 0.01 | 0.013 | 0.021 | 0.008 | 0.013 | 0.007 | 0.011 |
| Diliman QC. | 7.4 | 7.0 | 9.9 | 9.5 | 0.016 | -0.002 | 0.007 | 0.009 | 0.005 | 0.002 | 0.008 | 0.008 |

Comments see main manuscript text.

## S8.4 South America

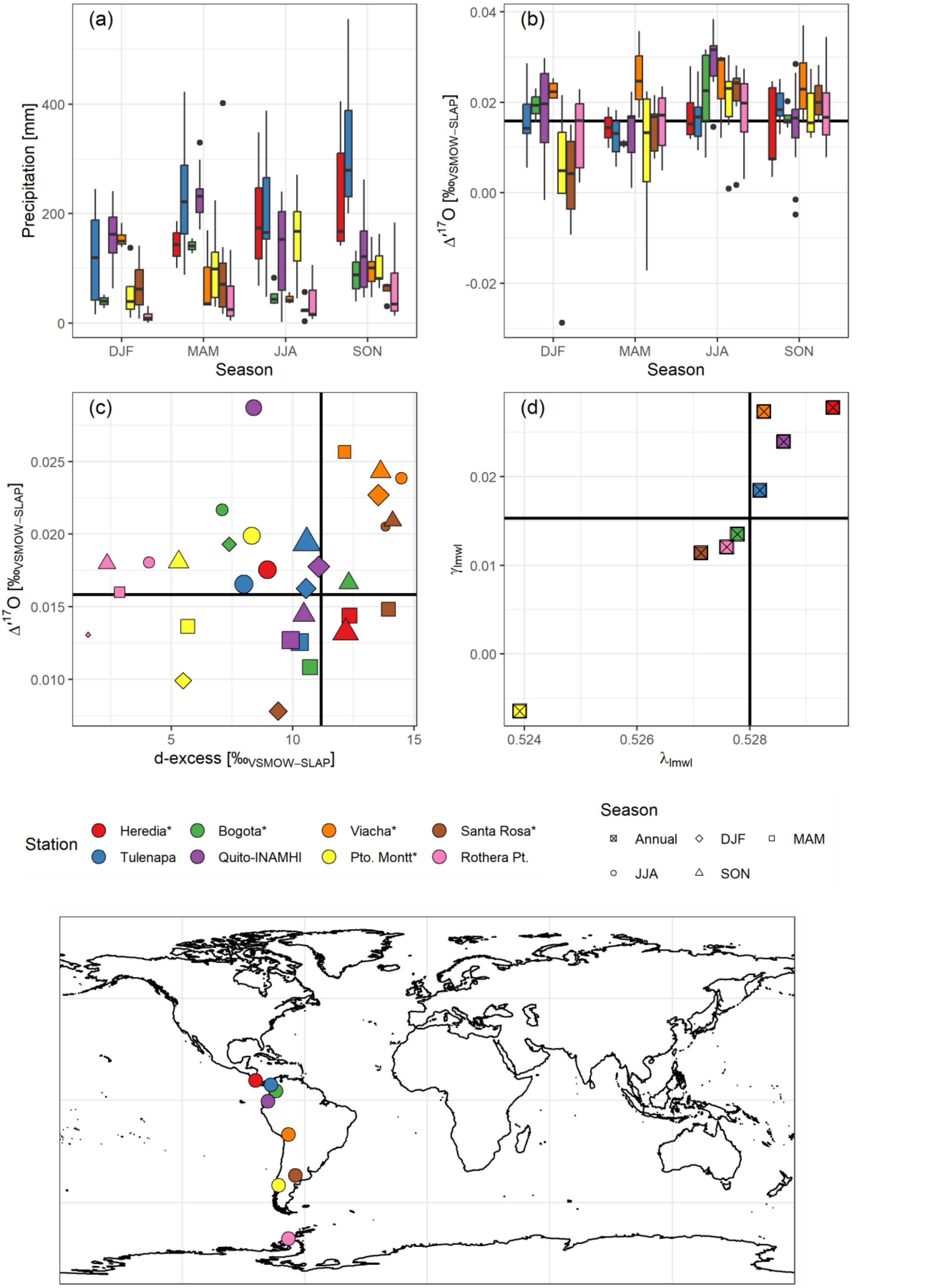


*Figure S9: South American transect. Seasonal distribution of precipitation depth (a), $\Delta'^{17}O$ (b), $\Delta'^{17}O$ vs. d-excess (c), $\lambda_{lmwl}$ vs. $\gamma_{lmwl}$ (d). Thick black lines indicate global mean $\Delta'^{17}O$ (b), $\Delta'^{17}O$ and d-excess (c), $\lambda_{gmwl}$ and $\gamma_{gmwl}$ (d). Stations marked with an asterisk failed to cover >50% of the precipitation with reproducible isotopic information and were discarded from the global analysis.*

*Table S19: Correlation matrix of $\lambda_{mwl}$, $\gamma_{mwl}$ and $\Delta'^{17}O$ against spatial, isotopic and climatologic variables for the South America transect. Spherical distance is measured in km from Heredia and overlaps largely with latitude. Asterisks (*, ** or ***) indicate significance of correlation at the 0.01, 0.001 and 0.0001 levels respectively.*

| **Independent variable** | **Dependent variables** | | | | | | |
|---|---|---|---|---|---|---|---|
| | $\lambda_{mwl}$ | $\gamma_{mwl}$ | **$\Delta'^{17}$O mean** | **$\Delta'^{17}$O DJF** | **$\Delta'^{17}$O MAM** | **$\Delta'^{17}$O JJA** | **$\Delta'^{17}$O SON** |
| Slope | | **0.90**** | | | | | |
| Latitude | 0.32 | 0.34 | 0.04 | 0.34 | 0.05 | 0.00 | 0.26 |
| Longitude | 0.16 | 0.13 | 0.28 | 0.03 | 0.29 | 0.06 | **0.71*** |
| Distance | 0.33 | 0.35 | 0.05 | 0.32 | 0.05 | 0.00 | 0.31 |
| $\delta'^{18}$O | 0.26 | 0.28 | 0.25 | 0.42 | 0.43 | 0.01 | 0.00 |
| d-excess | 0.11 | 0.21 | 0.05 | 0.29 | 0.07 | 0.11 | 0.00 |
| Precipitation | 0.15 | 0.18 | 0.05 | 0.25 | 0.19 | 0.04 | 0.15 |
| Air temp. (ERA5) | 0.09 | 0.10 | 0.08 | 0.00 | 0.14 | 0.01 | 0.15 |
| RH (ERA5) | 0.04 | 0.03 | 0.17 | 0.1 | 0.46 | 0.06 | 0.39 |

*Table S20: Seasonal median values of precipitation depth and median/IQR values of $\delta^{18}O$*

| **Station** | **Median precipitation [mm mo$^{-1}$]** | | | | **Median $\delta^{18}$O** | | | | **IQR $\delta^{18}$O** | | | |
|---|---|---|---|---|---|---|---|---|---|---|---|---|
| | **DJF** | **MAM** | **JJA** | **SON** | **DJF** | **MAM** | **JJA** | **SON** | **DJF** | **MAM** | **JJA** | **SON** |
| Heredia* | --- | 143.3 | 173.4 | 167.3 | --- | -6.66 | -10.12 | -8.20 | --- | 3.59 | 1.72 | 5.52 |
| Tulenapa | 119.4 | 221.9 | 165.8 | 278.9 | -3.68 | -6.93 | -8.19 | -7.37 | 3.56 | 5.55 | 1.05 | 2.28 |
| Bogota* | 39.8 | 141.2 | 43.6 | 87.9 | -10.32 | -5.73 | -11.18 | -9.70 | 0.22 | 0.35 | 3.22 | 4.38 |
| Quito INAMHI | 185.8 | 320.8 | 237.1 | 156.8 | -8.29 | -15.38 | -12.51 | -8.54 | 2.51 | 1.69 | 2.74 | 2.14 |
| Viacha* | 151.8 | 36.7 | 42.9 | 103.8 | -15.85 | -16.56 | -4.21 | -10.85 | 3.12 | 3.49 | 4.03 | 4.05 |
| Pto. Montt* | 39.4 | 99.0 | 167.9 | 81.6 | -4.43 | -5.12 | -6.50 | -5.18 | 2.83 | 0.75 | 1.29 | 2.98 |
| Santa Rosa* | 62.2 | 70.4 | 22.4 | 68.4 | -2.63 | -7.97 | -4.98 | -4.79 | 1.21 | 3.31 | 1.15 | 1.17 |
| Rothera Pt. | 8.8 | 24.9 | 16.2 | 34.9 | -13.29 | -13.75 | -15.98 | -16.09 | 3.09 | 1.73 | 0.93 | 1.75 |

*Table S21: Seasonal median values of d-excess and median/IQR values $\Delta'^{17}O$*

| **Station** | **Median *d*-excess** | | | | **Median *$\Delta'^{17}$O*** | | | | **IQR *$\Delta'^{17}$O*** | | | |
|---|---|---|---|---|---|---|---|---|---|---|---|---|
| | **DJF** | **MAM** | **JJA** | **SON** | **DJF** | **MAM** | **JJA** | **SON** | **DJF** | **MAM** | **JJA** | **SON** |
| Heredia* | --- | 12.3 | 9.6 | 11.6 | --- | 0.014 | 0.015 | 0.007 | --- | 0.005 | 0.007 | 0.016 |
| Tulenapa | 10.6 | 9.9 | 8.5 | 10.7 | 0.014 | 0.013 | 0.017 | 0.018 | 0.006 | 0.007 | 0.006 | 0.005 |
| Bogota* | 7.4 | 10.7 | 6.1 | 12.5 | 0.019 | 0.011 | 0.023 | 0.016 | 0.004 | 0.001 | 0.014 | 0.002 |
| Quito INAMHI | 10.9 | 10.8 | 9.4 | 10.7 | 0.020 | 0.017 | 0.032 | 0.016 | 0.015 | 0.008 | 0.007 | 0.006 |
| Viacha* | 13.3 | 12.1 | 12.3 | 13.2 | 0.022 | 0.025 | 0.029 | 0.023 | 0.003 | 0.010 | 0.009 | 0.010 |
| Pto. Montt* | 4.2 | 4.4 | 9.6 | 6.9 | 0.005 | 0.013 | 0.023 | 0.015 | 0.014 | 0.018 | 0.008 | 0.009 |
| Santa Rosa* | 7.7 | 15 | 14.8 | 13.3 | 0.004 | 0.017 | 0.024 | 0.020 | 0.015 | 0.008 | 0.005 | 0.006 |
| Rothera Pt. | -2.6 | 1.6 | 3.6 | 2.3 | 0.016 | 0.017 | 0.020 | 0.017 | 0.014 | 0.010 | 0.011 | 0.009 |

The South American domain does hardly yield quantifiable relationships between the variables investigated. The outlier point Puerto Montt is probably due to sample curation issues (even though the established detection metric using the d-excess does not really flag this). The significant (at the 0.01 level) correlation between $\Delta'^{17}$O and longitude during SON is probably due to the longitudinal RH gradient (end of the drier austral winter in Sta. Rosa and Rothera Pt. which are the easternmost stations), a pattern mirrored in MAM.

## S8.5 Eastern Mediterranean and Black Sea Basins

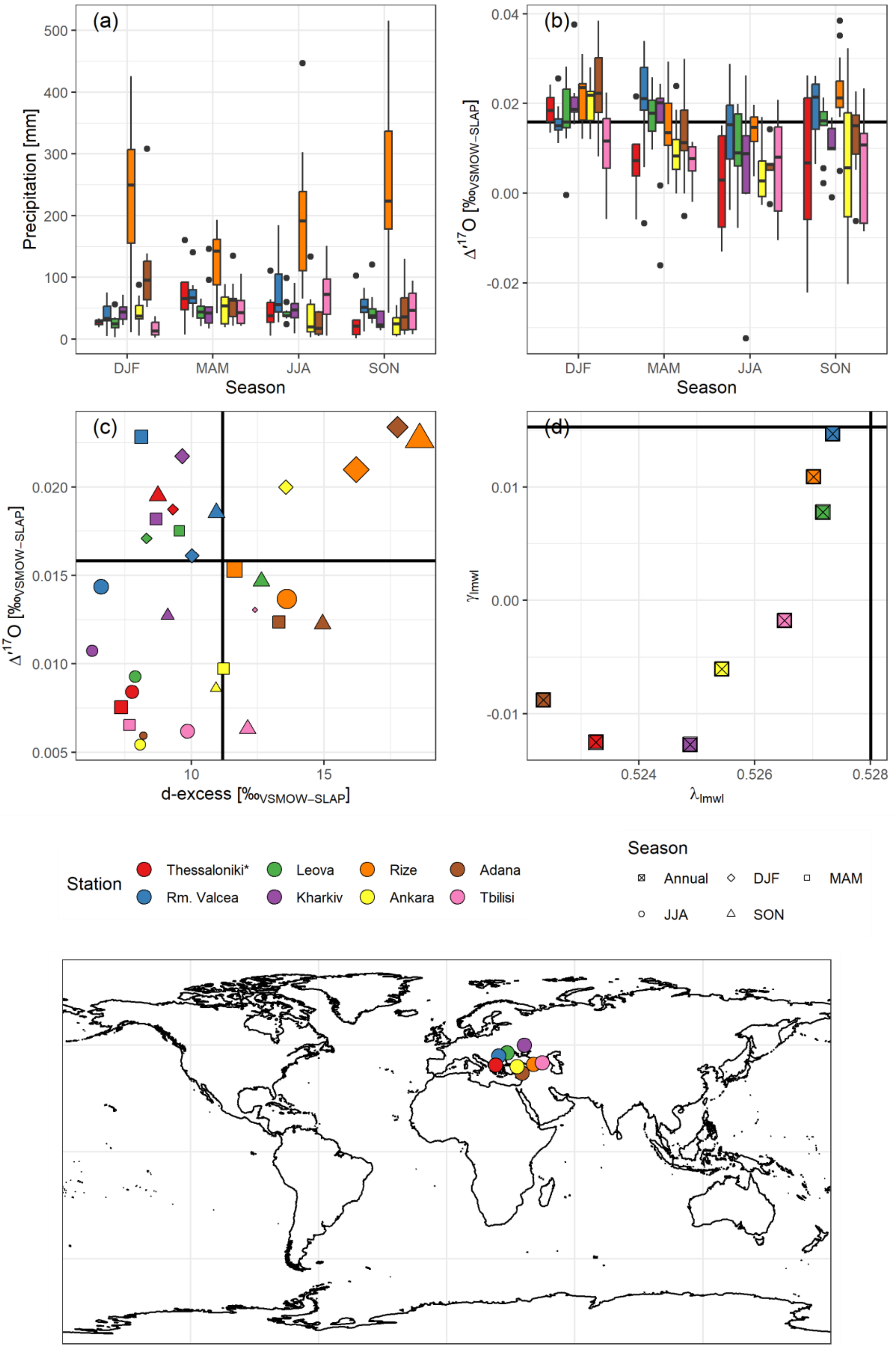


*Figure S10: Black Sea region. Seasonal distribution of precipitation depth (a), $\Delta'^{17}O$ (b), $\Delta'^{17}O$ vs. d-excess (c), $\lambda_{lmwl}$ vs. $\gamma_{lmwl}$ (d). Thick black lines indicate global mean $\Delta'^{17}O$ (b), $\Delta'^{17}O$ and d-excess (c), $\lambda_{gmwl}$ and $\gamma_{gmwl}$ (d). Stations marked with an asterisk failed to cover >50% of the precipitation with reproducible isotopic information and were discarded from the global analysis.*

*Table S22: Correlation matrix of $\lambda_{mwl}$, $\gamma_{mwl}$ and $\Delta'^{17}O$ against spatial, isotopic and climatologic variables for the Black Sea region. Spherical distance is measured in km from Thessaloniki. Asterisks (*, ** or ***) indicate significance of correlation at the 0.01, 0.001 and 0.0001 levels respectively.*

| **Independent variable** | **Dependent variables** | | | | | | |
|---|---|---|---|---|---|---|---|
| | $\lambda_{mwl}$ | $\gamma_{mwl}$ | $\Delta'^{17}O$ **mean** | $\Delta'^{17}O$ **DJF** | $\Delta'^{17}O$ **MAM** | $\Delta'^{17}O$ **JJA** | $\Delta'^{17}O$ **SON** |
| Slope | | 0.69 | | | | | |
| Latitude | 0.22 | 0.02 | 0.03 | 0.18 | 0.34 | 0.01 | 0.00 |
| Longitude | 0.02 | 0.00 | 0.20 | 0.01 | 0.38 | 0.17 | 0.24 |
| Distance | 0.05 | 0.00 | 0.16 | 0.02 | 0.20 | 0.17 | 0.24 |
| $\delta'^{18}O$ | 0.62 | 0.42 | 0.09 | 0.40 | 0.20 | 0.53 | 0.19 |
| d-excess | 0.00 | 0.09 | 0.21 | 0.39 | 0.18 | 0.43 | 0.11 |
| Precipitation | 0.08 | 0.23 | 0.24 | 0.26 | 0.06 | 0.22 | 0.36 |
| Air temp. (ERA5) | **0.70*** | 0.30 | 0.00 | 0.23 | 0.12 | 0.23 | 0.04 |
| RH (ERA5) | 0.03 | 0.29 | 0.61 | 0.07 | 0.25 | 0.07 | 0.65 |

*Table S23: Seasonal median values of precipitation depth and median/IQR values of $\delta^{18}O$*

| **Station** | **Median precipitation [mm mo$^{-1}$]** | | | | **Median $\delta^{18}O$** | | | | **IQR $\delta^{18}O$** | | | |
|---|---|---|---|---|---|---|---|---|---|---|---|---|
| | **DJF** | **MAM** | **JJA** | **SON** | **DJF** | **MAM** | **JJA** | **SON** | **DJF** | **MAM** | **JJA** | **SON** |
| Thessaloniki* | 27.9 | 65.3 | 37.6 | 20.8 | -5.87 | -5.17 | -3.73 | -3.32 | 1.93 | 1.17 | 3.16 | 5.33 |
| Rm. Valcea | 33.3 | 66.8 | 55.6 | 51.5 | -12.44 | -5.69 | -5.05 | -9.29 | 3.68 | 4.61 | 1.82 | 2.97 |
| Leova | 25.0 | 43.8 | 37.6 | 37.5 | -13.84 | -7.74 | -5.52 | -10.09 | 5.94 | 4.70 | 1.08 | 2.74 |
| Kharkiv | 43.7 | 41.9 | 47.1 | 22.6 | -12.53 | -9.18 | -4.86 | -8.85 | 0.84 | 5.92 | 2.03 | 2.63 |
| Rize | 249.6 | 142.2 | 191.5 | 223.6 | -10.24 | -7.85 | -4.49 | -8.32 | 2.11 | 3.23 | 1.02 | 1.95 |
| Ankara | 37.4 | 53.8 | 19.6 | 24.8 | -10.35 | -6.43 | -3.33 | -6.49 | 2.35 | 2.40 | 2.14 | 3.20 |
| Adana | 95.3 | 62.9 | 17.3 | 36 | -5.73 | -3.98 | -3.34 | -3.91 | 0.76 | 2.20 | 0.90 | 1.41 |
| Tbilisi | 12.7 | 42.7 | 72.4 | 46.5 | -12.08 | -6.82 | -3.68 | -6.56 | 2.18 | 2.91 | 2.56 | 4.79 |

*Table S24: Seasonal median values of d-excess and median/IQR values $\Delta'^{17}O$*

| **Station** | **Median *d*-excess** | | | | **Median $\Delta'^{17}O$** | | | | **IQR $\Delta'^{17}O$** | | | |
|---|---|---|---|---|---|---|---|---|---|---|---|---|
| | **DJF** | **MAM** | **JJA** | **SON** | **DJF** | **MAM** | **JJA** | **SON** | **DJF** | **MAM** | **JJA** | **SON** |
| Thessaloniki* | 10.6 | 9.5 | 4.5 | 2.1 | 0.018 | 0.007 | 0.003 | 0.007 | 0.005 | 0.007 | 0.020 | 0.027 |
| Rm. Valcea | 10.6 | 7.7 | 6.1 | 11.8 | 0.015 | 0.021 | 0.015 | 0.021 | 0.002 | 0.009 | 0.012 | 0.010 |
| Leova | 8.6 | 9.3 | 8.9 | 13.3 | 0.016 | 0.018 | 0.009 | 0.016 | 0.009 | 0.007 | 0.012 | 0.003 |
| Kharkiv | 9.7 | 7.8 | 4.9 | 8.0 | 0.019 | 0.020 | 0.009 | 0.010 | 0.003 | 0.005 | 0.013 | 0.005 |
| Rize | 16.4 | 11.5 | 12.9 | 19.1 | 0.024 | 0.013 | 0.015 | 0.021 | 0.008 | 0.009 | 0.005 | 0.006 |
| Ankara | 13.9 | 12 | 8.7 | 10.8 | 0.022 | 0.008 | 0.003 | 0.006 | 0.006 | 0.007 | 0.008 | 0.023 |
| Adana | 18.2 | 13.8 | 8.3 | 15.4 | 0.022 | 0.011 | 0.006 | 0.015 | 0.012 | 0.009 | 0.002 | 0.009 |
| Tbilisi | 12.1 | 8.1 | 9.8 | 12.3 | 0.012 | 0.008 | 0.008 | 0.011 | 0.011 | 0.005 | 0.019 | 0.020 |

In the Eastern Mediterranean and Black Sea region, the only common denominator was a significant (0.01 level) correlation between air temperature and the MWL slope $\lambda_{mwl}$. Relative humidity was important especially during the autumn season but not significant. Similarly, the $\Delta'^{17}O$ correlated to some extent with DJF and JJA $\delta'^{18}O$ and *d*-excess (the two seasons are the climatic end members in the region). As for the $\lambda_{mwl}/\gamma_{mwl}$ relationship, Adana (Eastern Mediterranean) and Thessaloniki (short record in addition to that) stood out, for reasons yet to be investigated.

## S8.6 Central Africa

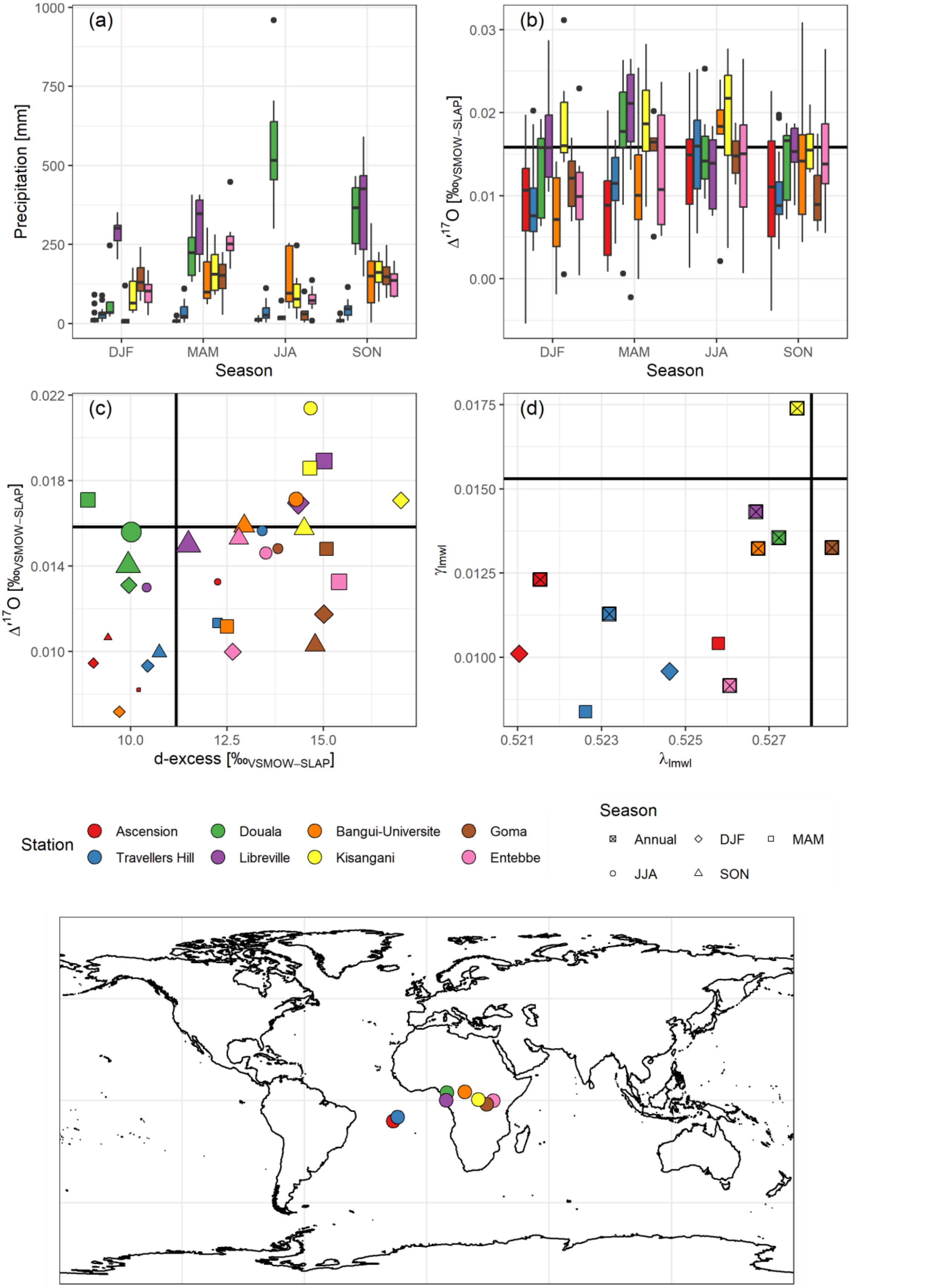


*Figure S11: Central African transect. Seasonal distribution of precipitation depth (a), $\Delta'^{17}O$ (b), $\Delta'^{17}O$ vs. d-excess (c), $\lambda_{lmwl}$ vs. $\gamma_{lmwl}$ (d). Thick black lines indicate global mean $\Delta'^{17}O$ (b), $\Delta'^{17}O$ and d-excess (c), $\lambda_{gmwl}$ and $\gamma_{gmwl}$ (d).*

*Table S25: Correlation matrix of $\lambda_{mwl}$, $\gamma_{mwl}$ and $\Delta'^{17}O$ against spatial, isotopic and climatologic variables for the Central African transect. Spherical distance is measured in km from Ascension Is. and overlaps largely with longitude. Asterisks (*, ** or ***) indicate significance of correlation at the 0.01, 0.001 and 0.0001 levels respectively.*

| Independent variable | Dependent variables | | | | | | |
|---|---|---|---|---|---|---|---|
| | $\lambda_{mwl}$ | $\gamma_{mwl}$ | $\Delta'^{17}O$ mean | $\Delta'^{17}O$ DJF | $\Delta'^{17}O$ MAM | $\Delta'^{17}O$ JJA | $\Delta'^{17}O$ SON |
| Slope | | 0.34 | | | | | |
| Latitude | **0.62*** | 0.35 | 0.46 | 0.25 | 0.42 | 0.22 | 0.24 |
| Longitude | **0.65*** | 0.26 | 0.15 | 0.03 | 0.38 | 0.25 | 0.05 |
| Distance | **0.64*** | 0.28 | 0.15 | 0.03 | 0.37 | 0.23 | 0.06 |
| $\delta'^{18}O$ | **0.66*** | 0.37 | 0.58 | 0.31 | **0.71*** | 0.04 | 0.15 |
| d-excess | 0.43 | 0.22 | 0.12 | 0.33 | 0.17 | 0.02 | 0.01 |
| Precipitation | 0.42 | 0.27 | 0.35 | 0.32 | 0.49 | 0.00 | 0.08 |
| Air temp. (ERA5) | 0.06 | 0.00 | 0.34 | 0.17 | 0.01 | 0.10 | 0.56 |
| RH (ERA5) | 0.12 | 0.37 | 0.42 | 0.21 | **0.74*** | 0.25 | 0.13 |

*Table S26: Seasonal median values of precipitation depth and median/IQR values of $\delta^{18}O$*

| Station | Median precipitation [mm $mo^{-1}$] | | | | Median $\delta^{18}O$ | | | | IQR $\delta^{18}O$ | | | |
|---|---|---|---|---|---|---|---|---|---|---|---|---|
| | DJF | MAM | JJA | SON | DJF | MAM | JJA | SON | DJF | MAM | JJA | SON |
| Ascension | 11.0 | 9.1 | 12.9 | 8.8 | +0.06 | -0.30 | +0.05 | +0.29 | 0.74 | 0.72 | 0.41 | 0.42 |
| Travellers Hill | 28.3 | 23.3 | 26.2 | 46.2 | -0.12 | -0.40 | -0.27 | -0.08 | 0.34 | 0.67 | 0.29 | 0.29 |
| Douala | 34.2 | 224.1 | 516.4 | 365.6 | -1.57 | -1.58 | -2.20 | -3.13 | 1.56 | 1.38 | 1.20 | 1.33 |
| Libreville | 293.7 | 341.6 | 19.4 | 428.2 | -1.42 | -2.81 | -0.17 | -3.04 | 1.05 | 0.77 | 1.11 | 1.79 |
| Bangui-Univ. | 7.0 | 99.0 | 96.0 | 149.8 | +3.25 | -1.26 | -2.85 | -0.01 | 5.01 | 4.58 | 2.57 | 3.90 |
| Kisangani | 64.9 | 156.2 | 76.9 | 161.2 | +0.15 | -3.34 | +0.05 | -2.53 | 1.46 | 2.44 | 1.57 | 2.12 |
| Goma | 130.1 | 151.8 | 30.3 | 147.8 | -0.11 | -2.75 | -1.54 | -1.87 | 1.43 | 2.42 | 0.35 | 1.15 |
| Entebbe | 102.0 | 252.0 | 73.0 | 136.1 | -1.34 | -2.80 | -1.21 | -2.10 | 2.82 | 1.17 | 2.22 | 2.44 |

*Table S27: Seasonal median values of d-excess and median/IQR values $\Delta'^{17}O$*

| Station | Median *d*-excess | | | | Median $\Delta'^{17}O$ | | | | IQR $\Delta'^{17}O$ | | | |
|---|---|---|---|---|---|---|---|---|---|---|---|---|
| | DJF | MAM | JJA | SON | DJF | MAM | JJA | SON | DJF | MAM | JJA | SON |
| Ascension | 10.3 | 10.6 | 12.1 | 9.1 | 0.011 | 0.009 | 0.015 | 0.011 | 0.008 | 0.009 | 0.008 | 0.012 |
| Travellers Hill | 10.4 | 12 | 13.6 | 10.8 | 0.008 | 0.011 | 0.016 | 0.009 | 0.005 | 0.005 | 0.008 | 0.004 |
| Douala | 11.1 | 8.4 | 10.2 | 10.2 | 0.016 | 0.018 | 0.014 | 0.017 | 0.010 | 0.007 | 0.005 | 0.008 |
| Libreville | 15.6 | 15.4 | 10.9 | 10.7 | 0.016 | 0.021 | 0.014 | 0.015 | 0.007 | 0.008 | 0.008 | 0.004 |
| Bangui-Univ. | 8.4 | 14.1 | 16.2 | 12.9 | 0.007 | 0.010 | 0.018 | 0.014 | 0.008 | 0.008 | 0.003 | 0.010 |
| Kisangani | 16.9 | 15.6 | 16.2 | 15.3 | 0.016 | 0.019 | 0.022 | 0.015 | 0.006 | 0.007 | 0.010 | 0.004 |
| Goma | 15.6 | 15.3 | 14.1 | 15.0 | 0.012 | 0.016 | 0.015 | 0.009 | 0.005 | 0.002 | 0.004 | 0.005 |
| Entebbe | 14.1 | 15.4 | 13.4 | 13.6 | 0.010 | 0.011 | 0.015 | 0.014 | 0.006 | 0.013 | 0.010 | 0.007 |

The Central African transect includes year-round wet sites but also such under the influences of northerly (Bangui, Douala; peak rain JJA) and southerly (Libreville, Ascension; peak rain DJF or MAM) movement of the ITCZ. This is visible most prominently in the response of $\Delta'^{17}O$ to $\delta'^{18}O$, precipitation depth and RH during MAM (onset or tail of rainy seasons). Also, this region is one of the few to show a quantifiable response of $\Delta'^{17}O$ (albeit not significant) to precipitation depth rather than temperature.

## S8.7 Europe

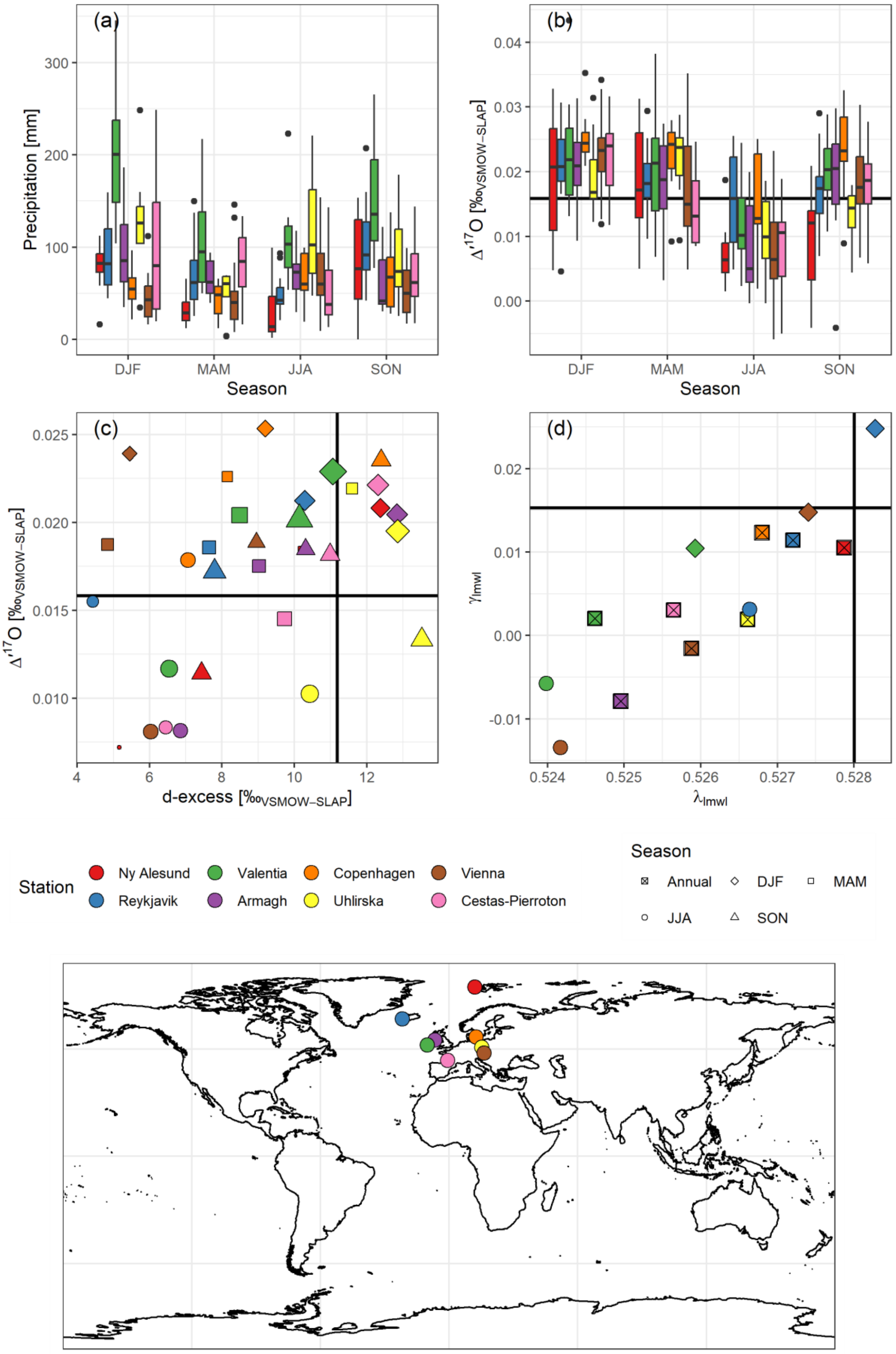


*Figure S12: Europe. Seasonal distribution of precipitation depth (a), $\Delta'^{17}O$ (b), $\Delta'^{17}O$ vs. d-excess (c), $\lambda_{lmwl}$ vs. $\gamma_{lmwl}$ (d). Thick black lines indicate global mean $\Delta'^{17}O$ (b), $\Delta'^{17}O$ and d-excess (c), $\lambda_{gmwl}$ and $\gamma_{gmwl}$ (d).*

*Table S28: Correlation matrix of $\lambda_{mwl}$, $\gamma_{mwl}$ and $\Delta'^{17}O$ against spatial, isotopic and climatologic variables for Europe. Spherical distance is measured in km from Cestas-Pierroton. Asterisks (*, ** or ***) indicate significance of correlation at the 0.01, 0.001 and 0.0001 levels respectively.*

| Independent variable | Dependent variables | | | | | | |
|---|---|---|---|---|---|---|---|
| | $\lambda_{mwl}$ | $\gamma_{mwl}$ | $\Delta'^{17}O$ mean | $\Delta'^{17}O$ DJF | $\Delta'^{17}O$ MAM | $\Delta'^{17}O$ JJA | $\Delta'^{17}O$ SON |
| Slope | | **0.75*** ** | | | | | |
| Latitude | 0.34 | 0.18 | 0.15 | 0.34 | 0.00 | 0.00 | 0.47 |
| Longitude | 0.00 | 0.04 | 0.04 | 0.00 | 0.05 | 0.05 | 0.02 |
| Distance | 0.40 | 0.17 | 0.14 | 0.29 | 0.02 | 0.00 | 0.44 |
| $\delta'^{18}O$ | **0.72**** | 0.34 | 0.05 | 0.00 | 0.15 | 0.00 | 0.13 |
| d-excess | 0.00 | 0.02 | 0.05 | 0.08 | 0.04 | 0.02 | 0.42 |
| Precipitation | 0.12 | 0.00 | 0.05 | 0.00 | 0.04 | 0.01 | 0.00 |
| Air temp. (ERA5) | **0.70**** | **0.64**** | 0.22 | 0.40 | 0.00 | 0.00 | 0.67 |
| RH (ERA5) | 0.01 | 0.14 | 0.11 | 0.16 | 0.09 | 0.02 | 0.01 |

*Table S29: Seasonal median values of precipitation depth and median/IQR values of $\delta^{18}O$*

| Station | Median precipitation [mm mo$^{-1}$] | | | | Median $\delta^{18}O$ | | | | IQR $\delta^{18}O$ | | | |
|---|---|---|---|---|---|---|---|---|---|---|---|---|
| | DJF | MAM | JJA | SON | DJF | MAM | JJA | SON | DJF | MAM | JJA | SON |
| Ny Ålesund | 82.2 | 28.5 | 13.6 | 76.7 | -10.05 | -9.32 | -10.99 | -10.44 | 0.94 | 3.1 | 4.42 | 2.40 |
| Reykjavik | 82.1 | 61.4 | 42.6 | 91.5 | -9.2 | -8.04 | -8.39 | -9.26 | 1.49 | 2.35 | 1.98 | 2.05 |
| Valentia | 200.3 | 94.8 | 103 | 135.4 | -5.63 | -4.89 | -4.19 | -4.92 | 1.52 | 1.25 | 0.95 | 0.56 |
| Armagh | 85.2 | 61.9 | 72.9 | 41.8 | -8.17 | -8.07 | -5.48 | -7.60 | 1.75 | 1.10 | 2.30 | 2.31 |
| Copenhagen | 54.7 | 48.5 | 58.7 | 67.1 | -10.5 | -9.30 | -6.30 | -8.92 | 2.14 | 5.35 | 0.92 | 2.76 |
| Uhlirška | 125.9 | 60.1 | 102.3 | 73.4 | -12.09 | -10.19 | -7.74 | -9.61 | 1.69 | 2.36 | 2.08 | 2.91 |
| Vienna | 43.0 | 40.0 | 60.0 | 50.0 | -11.91 | -7.95 | -5.43 | -9.13 | 3.31 | 3.55 | 0.82 | 4.23 |
| Cestas-P. | 80.0 | 84.5 | 38 | 61.5 | -5.90 | -5.10 | -4.21 | -5.30 | 1.2 | 1.80 | 1.60 | 1.44 |

*Table S30: Seasonal median values of d-excess and median/IQR values $\Delta'^{17}O$*

| Station | Median *d*-excess | | | | Median $\Delta'^{17}O$ | | | | IQR $\Delta'^{17}O$ | | | |
|---|---|---|---|---|---|---|---|---|---|---|---|---|
| | DJF | MAM | JJA | SON | DJF | MAM | JJA | SON | DJF | MAM | JJA | SON |
| Ny Ålesund | 9.6 | 9.7 | 4.3 | 6.7 | 0.021 | 0.017 | 0.006 | 0.012 | 0.016 | 0.013 | 0.005 | 0.011 |
| Reykjavik | 9.8 | 7.6 | 4.7 | 8.0 | 0.021 | 0.018 | 0.016 | 0.017 | 0.006 | 0.005 | 0.013 | 0.006 |
| Valentia | 10.9 | 7.2 | 6.5 | 9.8 | 0.022 | 0.021 | 0.010 | 0.020 | 0.010 | 0.011 | 0.008 | 0.006 |
| Armagh | 12.0 | 8.8 | 6.6 | 9.9 | 0.021 | 0.019 | 0.005 | 0.020 | 0.007 | 0.010 | 0.012 | 0.009 |
| Copenhagen | 9.2 | 8.1 | 8.1 | 12.2 | 0.024 | 0.024 | 0.013 | 0.023 | 0.003 | 0.006 | 0.011 | 0.007 |
| Uhlirška | 12.6 | 12.6 | 10.4 | 13.5 | 0.017 | 0.024 | 0.010 | 0.014 | 0.006 | 0.006 | 0.009 | 0.005 |
| Vienna | 4.9 | 4.1 | 5.6 | 9.3 | 0.023 | 0.015 | 0.006 | 0.018 | 0.005 | 0.012 | 0.009 | 0.007 |
| Cestas-P. | 9.6 | 9.7 | 4.3 | 6.7 | 0.021 | 0.017 | 0.006 | 0.012 | 0.016 | 0.013 | 0.005 | 0.011 |

The European domain sees a significant correlation between $\lambda_{mwl}$ and $\gamma_{mwl}$, and both correlated with temperature. Correlation between $\lambda_{mwl}$ and $\delta'^{18}O$ was significant at the 0.001 level. $\Delta'^{17}O$ correlated with temperature and latitude (which are auto-correlated) and d-excess mainly during fall, and to a lesser degree in winter. We attribute this behavior to the interplay of subtropical, Atlantic and polar air masses in different seasons. Precipitation depth (unsurprisingly) did not exert a relevant control over $\Delta'^{17}O$ but neither did RH.

## S8.8 Gulf Stream Region

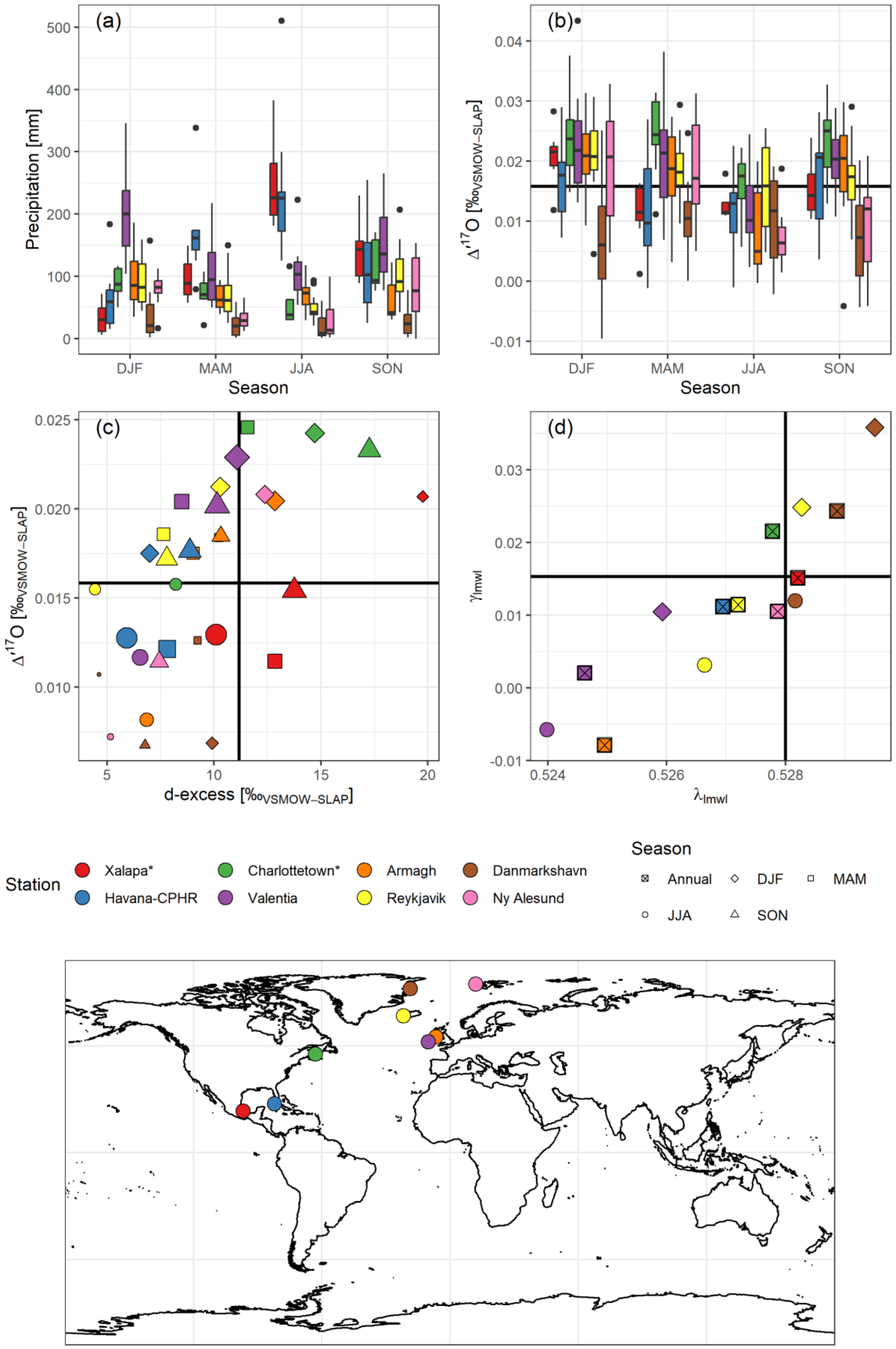


*Figure S13: Gulf Stream domain. Seasonal distribution of precipitation depth (a), $\Delta'^{17}O$ (b), $\Delta'^{17}O$ vs. d-excess (c), $\lambda_{lmwl}$ vs. $\gamma_{lmwl}$ (d). Thick black lines indicate global mean $\Delta'^{17}O$ (b), $\Delta'^{17}O$ and d-excess (c), $\lambda_{gmwl}$ and $\gamma_{gmwl}$ (d). Stations marked with an asterisk failed to cover >50% of the precipitation with reproducible isotopic information and were discarded from the global analysis.*

*Table S31: Correlation matrix of $\lambda_{mwl}$, $\gamma_{mwl}$ and $\Delta'^{17}O$ against spatial, isotopic and climatologic variables for the Gulf Stream domain. Spherical distance is measured in km from Xalapa. Asterisks (*, ** or ***) indicate significance of correlation at the 0.01, 0.001 and 0.0001 levels respectively.*

| Independent variable | Dependent variables | | | | | | |
|---|---|---|---|---|---|---|---|
| | $\lambda_{mwl}$ | $\gamma_{mwl}$ | $\Delta'^{17}O$ mean | $\Delta'^{17}O$ DJF | $\Delta'^{17}O$ MAM | $\Delta'^{17}O$ JJA | $\Delta'^{17}O$ SON |
| Slope | | **0.8***** | | | | | |
| Latitude | 0.09 | 0.06 | 0.04 | 0.14 | 0.04 | 0.15 | 0.20 |
| Longitude | 0.07 | 0.06 | 0.01 | 0.04 | 0.06 | 0.26 | 0.07 |
| Distance | 0.07 | 0.04 | 0.00 | 0.02 | 0.13 | 0.13 | 0.02 |
| $\delta'^{18}O$ | **0.44*** | 0.35 | 0.09 | 0.16 | 0.00 | 0.09 | 0.30 |
| d-excess | 0.02 | 0.12 | 0.38 | 0.26 | 0.00 | 0.38 | 0.58 |
| Precipitation | 0.28 | 0.08 | 0.11 | 0.23 | 0.01 | 0.01 | 0.39 |
| Air temp. (ERA5) | 0.33 | 0.40 | 0.04 | 0.17 | 0.04 | 0.24 | 0.23 |
| RH (ERA5) | 0.35 | 0.41 | 0.24 | 0.62 | 0.02 | 0.29 | 0.28 |

*Table S32: Seasonal median values of precipitation depth and median/IQR values of $\delta^{18}O$*

| Station | Median precipitation [mm $mo^{-1}$] | | | | Median $\delta^{18}O$ | | | | IQR $\delta^{18}O$ | | | |
|---|---|---|---|---|---|---|---|---|---|---|---|---|
| | DJF | MAM | JJA | SON | DJF | MAM | JJA | SON | DJF | MAM | JJA | SON |
| Xalapa* | 29.8 | 88.4 | 225.8 | 143.1 | -2.70 | -1.66 | -5.02 | -5.47 | 0.88 | 1.31 | 0.83 | 3.07 |
| Havana-CPHR | 59.0 | 161.7 | 225.4 | 102.5 | +0.40 | -2.45 | -1.99 | -1.43 | 2.35 | 2.38 | 2.93 | 2.35 |
| Charlottetown* | 87.2 | 70.3 | 37.8 | 93.2 | -10.95 | -9.18 | -8.33 | -8.61 | 2.85 | 1.93 | 1.14 | 2.19 |
| Valentia | 200.3 | 94.8 | 103.0 | 135.4 | -5.63 | -4.89 | -4.19 | -4.92 | 1.52 | 1.25 | 0.95 | 0.56 |
| Armagh | 85.2 | 61.9 | 72.9 | 41.8 | -8.17 | -8.07 | -5.48 | -7.60 | 1.75 | 1.10 | 2.3 | 2.31 |
| Reykjavik | 82.1 | 61.4 | 42.6 | 91.5 | -9.20 | -8.04 | -8.39 | -9.26 | 1.49 | 2.35 | 1.98 | 2.05 |
| Danmarkshavn | 21.0 | 20.5 | 8.4 | 23.9 | -17.59 | -17.24 | -15.2 | -17.49 | 3.87 | 5.01 | 4.16 | 3.71 |
| Ny Ålesund | 82.2 | 28.5 | 13.6 | 76.7 | -10.05 | -9.32 | -10.99 | -10.44 | 0.94 | 3.10 | 4.42 | 2.40 |

*Table S33: Seasonal median values of d-excess and median/IQR values $\Delta'^{17}O$*

| Station | Median *d*-excess | | | | Median $\Delta'^{17}O$ | | | | IQR $\Delta'^{17}O$ | | | |
|---|---|---|---|---|---|---|---|---|---|---|---|---|
| | DJF | MAM | JJA | SON | DJF | MAM | JJA | SON | DJF | MAM | JJA | SON |
| Xalapa* | 21.3 | 12.9 | 11.1 | 14.3 | 0.022 | 0.011 | 0.011 | 0.014 | 0.003 | 0.005 | 0.002 | 0.006 |
| Havana-CPHR | 4.4 | 8.5 | 6.0 | 6.6 | 0.018 | 0.010 | 0.013 | 0.021 | 0.008 | 0.013 | 0.007 | 0.011 |
| Charlottetown* | 14.2 | 10.3 | 9.8 | 18 | 0.024 | 0.024 | 0.018 | 0.025 | 0.008 | 0.007 | 0.006 | 0.008 |
| Valentia | 10.9 | 7.2 | 6.5 | 9.8 | 0.022 | 0.021 | 0.010 | 0.020 | 0.010 | 0.011 | 0.008 | 0.006 |
| Armagh | 12.0 | 8.8 | 6.6 | 9.9 | 0.021 | 0.019 | 0.005 | 0.020 | 0.007 | 0.010 | 0.012 | 0.009 |
| Reykjavik | 9.8 | 7.6 | 4.7 | 8.0 | 0.021 | 0.018 | 0.016 | 0.017 | 0.006 | 0.005 | 0.013 | 0.006 |
| Danmarkshavn | 9.0 | 7.6 | 4.3 | 6.7 | 0.006 | 0.01 | 0.012 | 0.007 | 0.012 | 0.006 | 0.013 | 0.012 |
| Ny Ålesund | 9.6 | 9.7 | 4.3 | 6.7 | 0.021 | 0.017 | 0.006 | 0.012 | 0.016 | 0.013 | 0.005 | 0.011 |

We quantitatively evaluated relationships of MWLs and $\Delta'^{17}O$ for the Gulf Stream domain with moderate success, probably due to the size of the region and complexity of its atmospheric circulations and drivers thereof during the different seasons (ranging from Caribbean hurricane seasons to the moments of the polar vortex). This is most obvious during MAM where no correlation could be found (this marks the beginning of the Caribbean rainy season, spring in the temperate latitudes and end of winter in the polar regions). There was a moderate correlation (not significant) between d-excess and $\Delta'^{17}O$ for most of the year, and for precipitation depth and temperature during SON. RH was most important during DJF.

## S8.9 Polar and Subpolar Domains

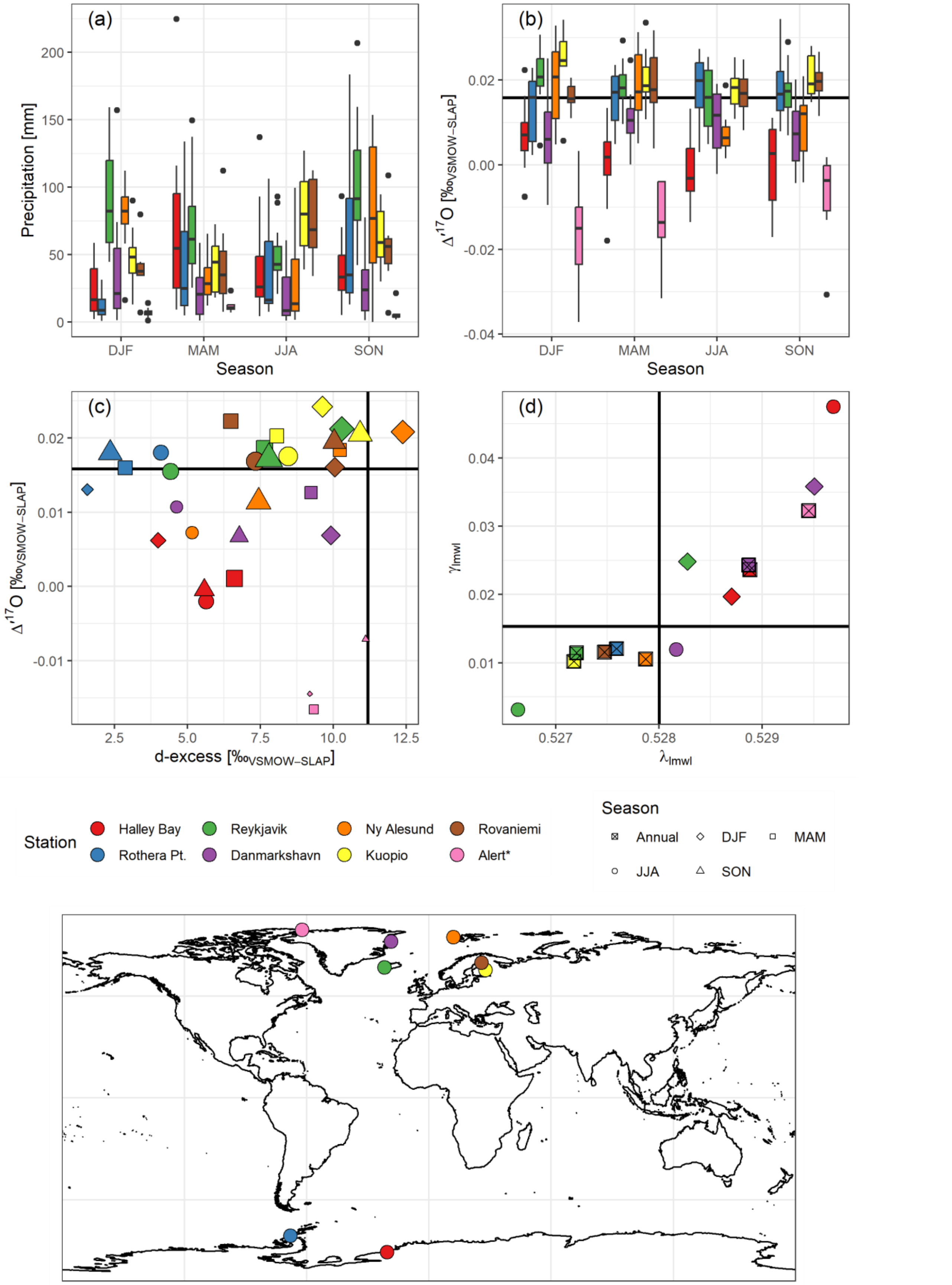


*Figure S14: Polar and subpolar domain (>60° N/S). Seasonal distribution of precipitation depth (a), $\Delta'^{17}O$ (b), $\Delta'^{17}O$ vs. d-excess (c), $\lambda_{lmwl}$ vs. $\gamma_{lmwl}$ (d). Thick black lines indicate global mean $\Delta'^{17}O$ (b), $\Delta'^{17}O$ and d-excess (c), $\lambda_{gmwl}$ and $\gamma_{gmwl}$ (d). Stations marked with an asterisk failed to cover >50% of the precipitation with reproducible isotopic information and were discarded from the global analysis.*

*Table S34: Correlation matrix of $\lambda_{mwl}$, $\gamma_{mwl}$ and $\Delta'^{17}O$ against spatial, isotopic and climatologic variables for the polar domains. Spatial variables were not evaluated due to non-contiguous domains. Caution is advised in the interpretation as the timing of seasons varies (northern and southern hemispheres). Asterisks (*, ** or ***) indicate significance of correlation at the 0.01, 0.001 and 0.0001 levels respectively. + indicates results when excluding the southern hemisphere sites.*

| Independent variable | Dependent variables | | | | | | |
|---|---|---|---|---|---|---|---|
| | $\lambda_{mwl}$ | $\gamma_{mwl}$ | $\Delta'^{17}O$ mean | $\Delta'^{17}O$ DJF | $\Delta'^{17}O$ MAM | $\Delta'^{17}O$ JJA | $\Delta'^{17}O$ SON |
| Slope | | **0.85*** ** | | | | | |
| $\delta'^{18}O$ | **0.66** ** | **0.59*** | **0.86** ** | **0.74*** | **0.89** ** | 0.53 | 0.6 |
| d-excess | 0.02 | 0.08 | 0.02 | 0 | 0 | 0.15 | 0.01 |
| Precipitation | 0.27 | 0.08 | 0.63 | 0.46 | 0.15 | 0.3 | 0.5 |
| Air temp. (ERA5) | **0.89*** ** | **0.78*** ** | **0.89** ** | **0.71*** | **0.91** ** | 0.37 ($0.95^+$) | 0.66 |
| RH (ERA5) | **0.47*** | **0.56*** | 0.4 | 0.38 | 0.26 | 0.11 ($0.70^+$) | 0.32 |

*Table S35: Seasonal median values of precipitation depth and median/IQR values of $\delta^{18}O$*

| Station | Median precipitation [mm $mo^{-1}$] | | | | Median $\delta^{18}O$ | | | | IQR $\delta^{18}O$ | | | |
|---|---|---|---|---|---|---|---|---|---|---|---|---|
| | DJF | MAM | JJA | SON | DJF | MAM | JJA | SON | DJF | MAM | JJA | SON |
| Halley Bay | 16.4 | 54.5 | 25.9 | 33.3 | -16.77 | -23.34 | -28.55 | -24.79 | 2.17 | 3.94 | 2.2 | 5.39 |
| Rothera Pt. | 8.8 | 24.9 | 16.2 | 34.9 | -13.29 | -13.75 | -15.98 | -16.09 | 3.09 | 1.73 | 0.93 | 1.75 |
| Reykjavik | 82.1 | 61.4 | 42.6 | 91.5 | -9.20 | -8.04 | -8.39 | -9.26 | 1.49 | 2.35 | 1.98 | 2.05 |
| Danmarkshavn | 21.0 | 20.5 | 8.4 | 23.9 | -17.59 | -17.24 | -15.20 | -17.49 | 3.87 | 5.01 | 4.16 | 3.71 |
| Ny Ålesund | 82.2 | 28.5 | 13.6 | 76.7 | -10.05 | -9.32 | -10.99 | -10.44 | 0.94 | 3.10 | 4.42 | 2.40 |
| Kuopio | 48.1 | 44.3 | 80.0 | 59.0 | -16.25 | -13.66 | -9.93 | -11.61 | 1.50 | 3.65 | 0.91 | 4.02 |
| Rovaniemi | 37.5 | 34.8 | 68.4 | 56.0 | -17.28 | -12.16 | -11.71 | -12.52 | 2.75 | 2.61 | 0.86 | 2.07 |
| Alert* | 6.8 | 10.5 | --- | 4.0 | -32.43 | -31.80 | --- | -30.00 | 3.49 | 0.73 | --- | 9.72 |

*Table S36: Seasonal median values of d-excess and median/IQR values $\Delta'^{17}O$*

| Station | Median *d*-excess | | | | Median $\Delta'^{17}O$ | | | | IQR $\Delta'^{17}O$ | | | |
|---|---|---|---|---|---|---|---|---|---|---|---|---|
| | DJF | MAM | JJA | SON | DJF | MAM | JJA | SON | DJF | MAM | JJA | SON |
| Halley Bay | 2.9 | 6.5 | 5.7 | 5.8 | 0.007 | 0.002 | -0.003 | 0.003 | 0.007 | 0.008 | 0.010 | 0.017 |
| Rothera Pt. | -2.6 | 1.6 | 3.6 | 2.3 | 0.016 | 0.017 | 0.020 | 0.017 | 0.014 | 0.010 | 0.011 | 0.009 |
| Reykjavik | 9.8 | 7.6 | 4.7 | 8.0 | 0.021 | 0.018 | 0.016 | 0.017 | 0.006 | 0.005 | 0.013 | 0.006 |
| Danmarkshavn | 9.0 | 7.6 | 4.3 | 6.7 | 0.006 | 0.010 | 0.012 | 0.007 | 0.012 | 0.006 | 0.013 | 0.012 |
| Ny Ålesund | 9.6 | 9.7 | 4.3 | 6.7 | 0.021 | 0.017 | 0.006 | 0.012 | 0.016 | 0.013 | 0.005 | 0.011 |
| Kuopio | 9.8 | 8.5 | 8.7 | 10.9 | 0.025 | 0.019 | 0.018 | 0.019 | 0.006 | 0.006 | 0.006 | 0.009 |
| Rovaniemi | 10.3 | 4.8 | 7.2 | 9.5 | 0.016 | 0.018 | 0.017 | 0.020 | 0.004 | 0.011 | 0.006 | 0.004 |
| Alert* | 10.3 | 7.4 | --- | 9.9 | -0.015 | -0.014 | --- | -0.004 | 0.014 | 0.013 | --- | 0.011 |

For the polar domain (caveat: Two stations were located in the southern hemisphere), the role of temperature was significant when looking at the distribution of $\lambda_{mwl}$ and $\gamma_{mwl}$. From Figure S14, it seems there is further discrimination between oceanic-influenced stations (Reykjavik, Kuopio, Rovaniemi) and continental ones (Halley Bay, Danmarkshavn, Alert); however, this observation could not be quantified due to the small number of data points. Air temperature was of similar relevance for $\Delta'^{17}O$. Further to that, RH was a significant driver of $\Delta'^{17}O$ except during JJA. We concede that for JJA, the outliers in both the temperature and humidity correlations were the sites in Halley Bay and Rothera Pt.; the $R^2$ of regressions markedly improved when omitting the southern hemisphere sites.

# S9 Line-conditioned $^{17}$O-Excess

We calculated the *lc*-excess and the $\Delta'^{17}O_{lc}$ based on the following LMWLs for the Vienna GNIP station (both weighted and based on the 2015-21 record):

$$lc = \delta^2H - 8.05 * \delta^{18}O - 6.65 \quad \text{(Equation 19)}$$

$$\Delta'^{17}O_{lc} = \delta'^{17}O - 0.5259 * \delta'^{18}O - 0.0015 \quad \text{(Equation 20)}$$

We re-analyzed available archived samples from within a range of ca. 100 km around the Vienna GNIP station in Vienna, Lower Austria and Burgenland states including standing surface waters and air moisture samples. We wish to emphasize that the samples we chose for this table and Figure 6 in the main manuscript resulted from opportunistic sampling efforts or comprised leftovers from measurement campaigns not initially related to this study. We acknowledge our colleagues A. Harjung for making the Danube samples available, and D. Brummer for the air moisture sampling. GNIP-Vienna samples were collected by the Geosphere Austria (formerly Central Agency for Meteorology and Geodynamics). All other samples were collected by the authors.

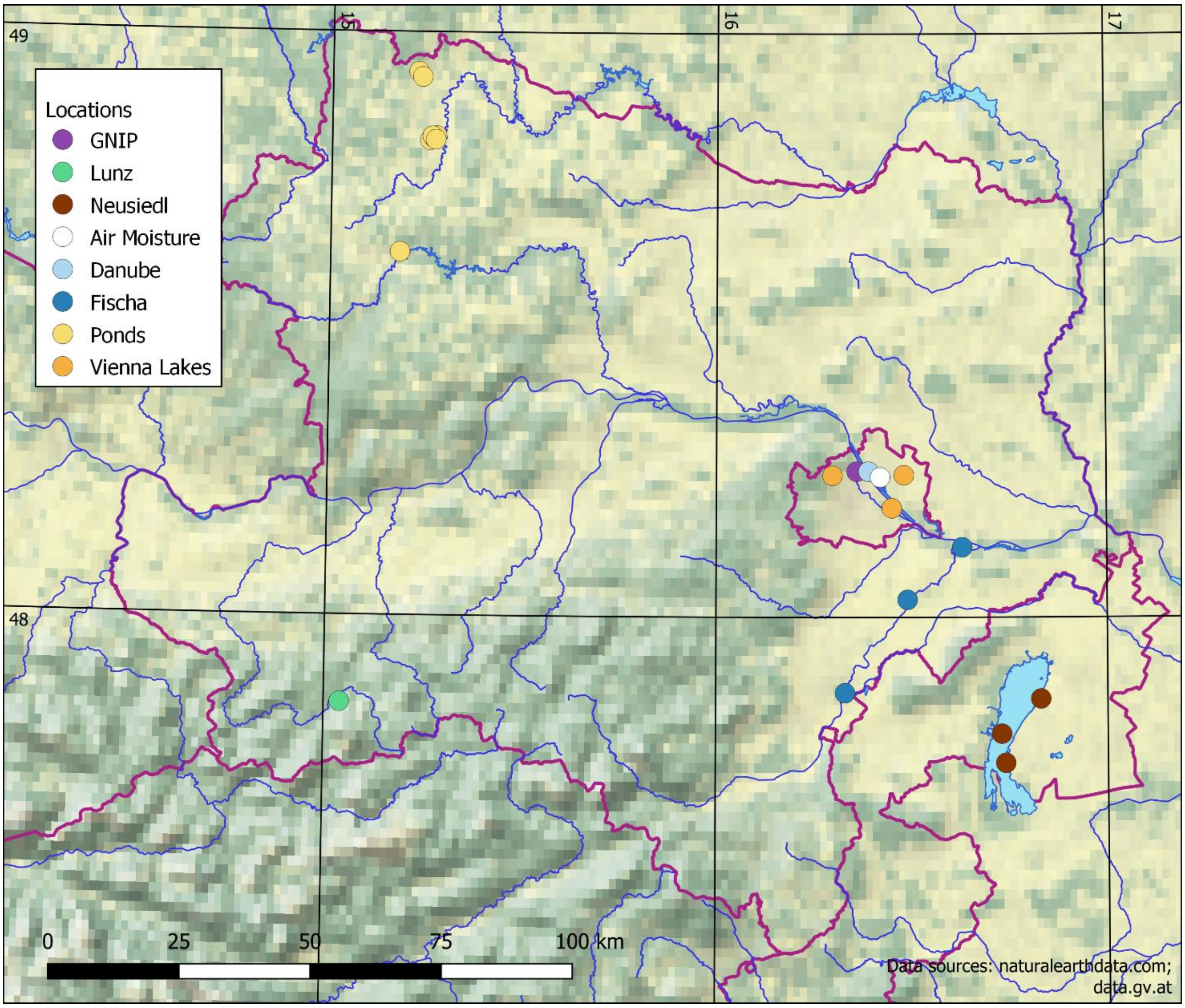


*Figure S15: Location map of the samples in Vienna, Lower Austria and Burgenland. Figure created with QGIS 3.4 (QGIS Association, http://www.qgis.org)*

*Table S37: Samples presented in Fig. 6 of the manuscript.*

| Site | Description | Latitude | Longitude | Type | n | Dates | $\delta^{18}O$ | d-exc. | *lc* | $\Delta'^{17}O$ | $\Delta'^{17}O_{lc}$ |
|---|---|---|---|---|---|---|---|---|---|---|---|
| GNIP | Vienna Hohe Warte | 48.25 | 16.36 | Precipitation | 82 | Jan 2015-Dec 2021 | -8.67 ± 3.26 | 5.2 ± 4.7 | -1.1 ± 4.7 | 0.016 ± 0.009 | -0.001 ± 0.007 |
| IAEA-IHL | Air moisture (chiller condensate) | 48.24 | 16.42 | Air moisture | 12 | Jul-Sep 2022 | -7.28 ± 2.00 | 15.6 ± 4.2 | 9.3 ± 4.3 | 0.023 ± 0.010 | 0.009 ± 0.007 |
| Neusiedler See | Terminal lake, shallow | 47.75 – 47.86 | 16.73 – 16.83 | Lake water | 3 | Aug 2020 Aug 2021 Aug 2022 | +1.58 ± 0.94 | -19.1 ± 3.7 | -25.8 ± 3.8 | -0.029 ± 0.006 | -0.024 ± 0.006 |
| Lunzer See | Small alpine lake, fast turnover | 47.85 | 15.04 | Lake water | 10 | Feb 2022 Apr 2022 | -10.96 ± 0.35 | 11.5 ± 0.5 | 5.3 ± 0.5 | 0.010 ± 0.005 | -0.012 ± 0.005 |
| Waldviertel | Small fishing ponds | 48.4 – 48.9 | 14.9 – 15.4 | Lake Water | 27 | Aug-Oct 2020 | -5.48 ± 1.52 | -3.0 ± 4.8 | -9.4 ± 4.9 | 0.004 ± 0.008 | -0.006 ± 0.006 |
| Lakes in Vienna | Panozzalacke probably hydraulically connected to Danube; others not | 48.18 – 48.24 | 16.33 – 16.50 | Lake Water | 4 | Jun 2023 | -8.25 ± 1.55 | 2.2 ± 4.7 | -4.1 ± 4.8 | 0.002 ± 0.008 | -0.014 ± 0.005 |
| Danube river | Danube @ BVS school, Vienna | 48.24 | 16.39 | River water | 8 | Oct 2022-May 2023 | -10.66 ± 0.56 | 9.6 ± 0.2 | 3.4 ± 0.2 | 0.015 ± 0.004 | -0.006 ± 0.004 |
| Fischa river | Source to mouth (snapshot) | 47.87 – 48.11 | 16.32 – 16.61 | River water | 5 | July 2020 | -10.26 ± 0.07 | 9.2 ± 0.5 | 3.0 ± 0.5 | 0.013 ± 0.006 | -0.008 ± 0.006 |

# S10 Canadian Arctic Dataset

We performed a separate evaluation for the $\lambda_{ref}$, $\gamma_{ref}$, and $\Delta'^{17}O$ for five additional stations in the Canadian Arctic to augment the coverage in cold climates. These samples originated from an opportunistic acquisition and were not initially slated for inclusion in this study because of concerns regarding the compatibility of sampling, and sample curation and analysis which partially followed different pathways:

- The main aim of the sampling regime were radionuclides; Health Canada made aliquots available for $\delta^{18}O$, $\delta^{2}H$ and $^{3}H$ analysis. On occasion, $^{3}H$ samples were further aliquoted for $\delta^{18}O$ and $\delta^{2}H$ analysis. Sample storage was in HDPE bottles; an aspect already discussed in S4 and S5.
- The sampling period available was from cold season 2018/19 to cold season 2021/22, while most other samples in this study cover a period of 2015-18 or 2015-21 (extended period stations).
- We chose the sites in Alert, Resolute, Iqaluit, Inuvik and Yellowknife to append additional measurements from arctic and subarctic climates. Given the sampling facilities, we excluded the period from May to August by default (because of temperatures reaching > 0°C, fostering post-depositional evaporation from the sampling device). This forced to perform the data analysis on as little as 39, 52, 45, 52 and 47% of the total 2018-22 precipitation, respectively.
- Sample analysis (initial as well as for the $\delta^{17}O$ re-measurement project) showed that a substantial fraction of samples had a lighter $\delta^{18}O$, $\delta^{17}O$ and $\delta^{2}H$ than our depleted RM (USGS46). We chose to not alter the analytical protocol (which had not aimed for analyzing samples from the high Arctic); however, this has to be kept in mind for the interpretation and is one of the reasons. These fractions were as high as 61% for Alert and 33% in Resolute; for the other three it was between 13-15%.

***We urge the readers to take these aspects in mind when interpreting the patterns observed.*** We are aware that cold-climate locations are important for understanding the role of $\delta^{17}O$ and $\Delta'^{17}O$ in the water cycle, but if artifacts resulting from the constraints above are considered, ambiguous explanations are possible. Considering that often, a $\lambda_{obs}$ for a dataset that includes waters having undergone evaporative processes, is lower than the $\lambda_{ref}$ or our $\lambda_{gmwl}$, it must be taken into consideration that some of the patterns observed, especially the good correlation between $\lambda_{lmwl}$ and MAT for temperatures <8 °C (an arbitrary threshold), can be beset by the artifacts above. Therefore, the five stations are shown, with the mean meteorological values calculated for September to April only, are shown in the figures but are not included into any of the calculations (GMWL, correlations etc.) unless expressly stated. The tables S38 to S40 provide the field and isotopic information akin to tables S5 and S6.

*Table S38: List of Canadian Arctic stations. The Köppen-Geiger climate classification is based on data from Environment Canada (https://climate.weather.gc.ca/climate_normals/index_e.html)*

| Station [1)] | Altitude | Latitude | Longitude | Precipitation | Mean AT | Mean RH | Climate |
|---|---|---|---|---|---|---|---|
| | m.a.s.l. | Decimal Degrees | | mm/mo. [2)] | °C [2)] | % [2)] | Köppen |
| *Alert Sept-Apr* | *62* | *82.500* | *-62.367* | *10.1* | *-23.1* | *75.1* | *E* |
| *Inuvik Sept-Apr* | *103* | *68.361* | *-133.730* | *19.0* | *-13.1* | *77.4* | *D* |
| *Iqaluit Sept-Apr* | *33.5* | *63.750* | *-68.522* | *8.6* | *-18.3* | *79.2* | *E* |
| *Resolute Sept-Apr* | *30* | *74.704* | *-94.829* | *13.9* | *-14.5* | *77.5* | *E* |
| *Yellowknife Sept-Apr* | *205.7* | *67.450* | *-114.367* | *12.4* | *-11.6* | *77.0* | *D* |

*Table S39: List of Canadian Arctic GNIP stations analyzed in this study*

| Station [1)] | PPT Src. | Sampling Period | n | Storage | analyzed | useful |
|---|---|---|---|---|---|---|
| | [3)] | | | [4)] | % of precipitation | |
| *Alert Sept-Apr* | *OBS* | *2018-22* | *22* | *Plastic** | *45.2%* | *43.0%* |
| *Inuvik Sept-Apr* | *OBS* | *2018-22* | *22* | *Plastic* | *57.8%* | *52.2%* |
| *Iqaluit Sept-Apr* | *OBS* | *2018-22* | *27* | *Plastic* | *76.1%* | *61.6%* |
| *Resolute Sept-Apr* | *OBS* | *2018-22* | *22* | *Plastic* | *64.6%* | *62.0%* |
| *Yellowknife Sept-Apr* | *OBS* | *2018-22* | *26* | *Plastic* | *62.6%* | *55.8%* |

*Table S40: Summary of the isotopic characteristics of the GNIP stations chosen.*

| Station [1)] | $\delta^{17}O$ | $\delta^{18}O$ | $\delta^{2}H$ | d-excess | $\Delta'^{17}O$ | $\lambda_{lmwl}$ | $\gamma_{lmwl}$ | $\lambda_{lmwl}$ |
|---|---|---|---|---|---|---|---|---|
| | Weighted mean in ‰$_{VSMOW-SLAP}$ | | | | | weighted | | Wt., $\gamma$=0 |
| *Alert Sept-Apr* | *-15.978* | *-30.018* | *-231.3* | *8.8* | *-0.012* | *0.5295* | *0.0323* | *0.5284* |
| *Inuvik Sept-Apr* | *-11.789* | *-22.217* | *-171.2* | *6.5* | *0.007* | *0.5285* | *0.0185* | *0.5277* |
| *Iqaluit Sept-Apr* | *-10.755* | *-20.283* | *-155.3* | *6.9* | *0.009* | *0.5289* | *0.0272* | *0.5276* |
| *Resolute Sept-Apr* | *-12.667* | *-23.844* | *-182.7* | *8.1* | *-0.002* | *0.5297* | *0.0378* | *0.5282* |
| *Yellowknife Sept-Apr* | *-12.229* | *-23.059* | *-180.1* | *4.4* | *0.015* | *0.5289* | *0.0364* | *0.5274* |

1) Italics = not included in global calculations.
2) Mean values refer to the periods January-April and September-December combined of the sampling period
3) Precipitation data source: OBS – Observed, ERA5 – ERA-5 reanalysis, GPCC – Global Precipitation Climatology Center, GLOB – ERA-5/GPCC globally weighted, ENS – Gaps in observed data filled by one of the above models.
4) Stations marked with * had aliquots taken from HDPE bottles initially submitted for tritium analysis

## References


1 Brand, W.A., Coplen, T.B., Vogl, J., Rosner, M. & Prohaska T. Assessment of international reference materials for isotope-ratio analysis (IUPAC Technical Report). *Pure and Applied Geochemistry* **86** (3), 425-467

2 Rozanski, K., Araguás-Araguás, L., & Gonfiantini, R. Isotopic patterns in modern global precipitation. Climate change in continental isotopic records. *Geophysical Monograph* **78**, 1–36 (1993).

3 Craig H. Isotopic variations in meteoric waters. *Science* **133**, 1702–1703 (1961).

4 Dansgaard W. Stable isotopes in precipitation. *Tellus* **16** (4), 436-468 (1964).

5 Landwehr, J. M., & Coplen, T. B. Line-conditioned excess: a new method for characterizing stable hydrogen and oxygen isotope ratios in hydrologic systems. *International conference on isotopes in environmental studies*, 132-135 (2006).

6 Luz, B., & Barkan, E. Variations of $^{17}O/^{16}O$ and $^{18}O/^{16}O$ in meteoric waters. *Geochimica et Cosmochimica Acta*, **74**(22), 6276-6286 (2010).

7 Aron, P. G. et al. Triple oxygen isotopes in the water cycle. *Chemical Geology*, **565**, 120026 (2021).

8 Angert, A., Cappa, C. D., & DePaolo, D. J. Kinetic 17O effects in the hydrologic cycle: Indirect evidence and implications. *Geochimica et Cosmochimica Acta*, **68**(17), 3487-3495 (2004).

9 Pierchala, A. et al. High-precision measurements of $\delta^2H$, $\delta^{18}O$ and $\delta^{17}O$ in water with the aid of cavity ring-down laser spectroscopy. *Isotopes in Environmental and Health studies*, **55**(3), 290-307 (2019).

10 Gröning, M. Improved water $\delta^2H$ and $\delta^{18}O$ calibration and calculation of measurement uncertainty using a simple software tool. *Rapid Communications in Mass Spectrometry*, **25**(19), 2711-2720 (2011).

11 Gröning, M. TEL Technical Note No. 01: SICalib User Manual (Stable Isotope Calibration for routine δ-scale measurements) Ver2.16j, available online: https://nucleus.iaea.org/sites/ReferenceMaterials/Shared%20Documents/Publications/TechnicalNotes/TELTechNote01_SICalibUserManual.pdf (accessed 2023-07-23)

12 Pierchala A. et al. Triple-isotope calibration of in-house water standards supplemented by determination of $^{17}O$ content of USGS49-50 reference materials using cavity ring-down laser spectrometry. *Isotopes in Environmental and Health Studies*, **57**(3), 254-261 (2021).

13 Berman, E. S., Levin, N. E., Landais, A., Li, S., & Owano, T. Measurement of $\delta^{18}O$, $\delta^{17}O$, and $^{17}O$-excess in water by off-axis integrated cavity output spectroscopy and isotope ratio mass spectrometry. *Analytical Chemistry*, **85**(21), 10392-10398 (2013).

14 Terzer-Wassmuth, S., Wassenaar, L. I., Araguás-Araguás, L. J., & Stumpp, C. Balancing precision and throughput of $\delta^{17}O$ and $\Delta'^{17}O$ analysis of natural waters by Cavity Ringdown Spectroscopy. *MethodsX*, 10, 102150 (2023).

15 Stephan T. & Trappitsch R. Reliable uncertainties: Error correlation, rotated error bars, and linear regressions in three-isotope plots and beyond. *International Journal of Mass Spectrometry*, **491**, 117053 (2023).

16 Tian, C., Wang, L., Kaseke, K. F., & Bird, B. W. Stable isotope compositions ($\delta^2H$, $\delta^{18}O$ and $\delta^{17}O$) of rainfall and snowfall in the central United States. *Scientific Reports*, **8**(1), 1-15 (2018).

17 Kaseke, K. F. et al. Precipitation origins and key drivers of precipitation isotope ($^{18}O$, $^2H$, and $^{17}O$) compositions over Windhoek. *Journal of Geophysical Research: Atmospheres*, **123**(14), 7311-7330 (2018).

18 Uechi, Y., & Uemura, R. Dominant influence of the humidity in the moisture source region on the 17O-excess in precipitation on a subtropical island. *Earth and Planetary Science Letters*, **513**, 20-28 (2019).

19 Tian, C., Wang, L., Tian, F., Zhao, S., & Jiao, W. Spatial and temporal variations of tap water $^{17}$O-excess in China. *Geochimica et Cosmochimica Acta*, **260**, 1-14 (2019).

20 He, S. et al. Understanding tropical convection through triple oxygen isotopes of precipitation from the maritime continent. *Journal of Geophysical Research: Atmospheres*, **126**(4), e2020JD033418 (2021).

21 Lin, Y., Clayton, R.N., Huang, L., Nakamura, N. and Lyons, J.R. Oxygen isotope anomaly observed in water vapor from Alert, Canada and the implication for the stratosphere. *Proceedings of the National Academy of Sciences*, **110** (39), 15608-15613

22 Bhattacharya, S., Pal, M., Panda, B., & Pradhan, M. Spectroscopic investigation of hydrogen and triple-oxygen isotopes in atmospheric water vapor and precipitation during Indian monsoon season. *Isotopes in Environmental and Health Studies*, **57**(4), 368-385 (2021).

23 Leuenberger, M. C., & Ranjan, S. Disentangle kinetic from equilibrium fractionation using primary ($\delta^{17}$O, $\delta^{18}$O, $\delta$D) and secondary ($\Delta^{17}$O, *dex*) stable isotope parameters on samples from the Swiss precipitation network. *Frontiers in Earth Science*, **9**, 598061 (2021).

24 Landais, A., Ekaykin, A., Barkan, E., Winkler, R. & Luz, B. Seasonal variations of $^{17}$O-excess and d-excess in snow precipitation at Vostok station, East Antarctica. *Journal of Glaciology*, **58**, 725-733 (2012).

25 Affolter, S., Häuselmann, A. D., Fleitmann, D., Häuselmann, P., & Leuenberger, M. (2015). Triple isotope ($\delta$D, $\delta^{17}$O, $\delta^{18}$O) study on precipitation, drip water and speleothem fluid inclusions for a Western Central European cave (NW Switzerland). *Quaternary Science Reviews*, **127**, 73-89 (2015).

26 Giménez, R., Bartolomé, M., Gázquez, F., Iglesias, M., & Moreno, A. Underlying Climate Controls in Triple Oxygen ($^{16}$O, $^{17}$O, $^{18}$O) and Hydrogen ($^{1}$H, $^{2}$H) Isotopes Composition of Rainfall (Central Pyrenees). *Frontiers in Earth Science*, **9**, 633698 (2021).

27 Putman, A. L., Fiorella, R. P., Bowen, G. J., & Cai, Z. A global perspective on local meteoric water lines: Meta-analytic insight into fundamental controls and practical constraints. *Water Resources Research*, **55** (8), 6896-6910 (2019).
